# Chemically Fueled Active Transport Cascades in Large Unilamellar Vesicles

**DOI:** 10.1021/jacs.6c11418

**Published:** 2026-08-03

**Authors:** Willow Baxter, Laura E. Bickerton, Kaiyuan Liang, Emanuele Penocchio, Juan A. Aguilar, Giulio Ragazzon, Stefan Borsley

**Affiliations:** † Department of Chemistry, 3057 Durham University, Stockton Road, Durham DH1 3LE, U.K.; ‡ Institut de Science et d’Ingénierie Supramoléculaires (ISIS), CNRS & icFRC, UMR 7006, 129648 University of Strasbourg, 8 Allée Gaspard Monge, Strasbourg 67000, France; § Strasbourg Institute for Advanced Studies (USIAS), University of Strasbourg, 5 allée du Général Rouvillois, Strasbourg 67000, France

## Abstract

Biology routinely transduces energy stored in chemical bonds to create transmembrane gradients, which are themselves a crucial on-demand energy store to drive life’s vital processes. We report a strategy for the chemically fueled active transport of molecular cargo in large unilamellar vesicles. We outline the features required for driving active transport across phospholipid bilayers. Through a series of experiments in both hybrid lipid–liquid supported membranes and biomimetic vesicles, we demonstrate the transmembrane transport of our diacid cargo and quantify key performance indicators of our system. The active transport mechanism gives rise to an associated generation of transmembrane pH gradients of up to ∼0.3 pH units, comparable to the gradient required to drive ATP synthase. We harness this gradient as an energy source to drive a secondary active transport process through symport/antiport mechanisms. These chemically fueled active transport cascades demonstrate the successive transduction of free energy from chemical bonds to transmembrane proton gradients to transmembrane Na^+^ or Cl^–^ gradients. Our work establishes the first example of chemically fueled active transport in unilamellar vesicles and demonstrates how transmembrane gradients can both absorb energy and transfer it as part of a greater systems-level, life-like, compartmentalized nanotechnology.

## Graphical abstract

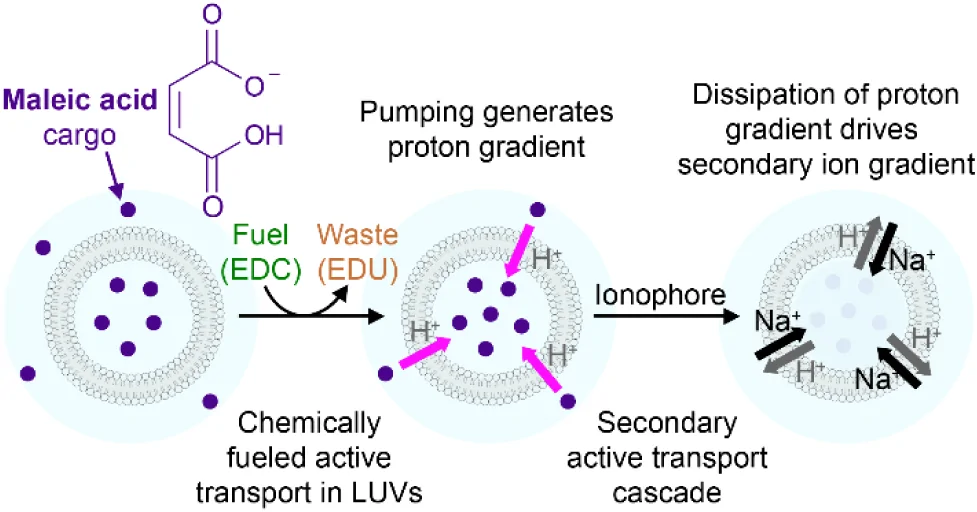

## Introduction

Transmembrane gradients provide a universal energy source in biological systems,^1–3^ underpinning virtually every example of sophisticated task performance.^4^ Some bacteria directly harness gradients around volcanic vents,^5^ while the formation of transmembrane proton gradients is the ultimate result of both photosynthesis^3,6^ and respiration,^1^ where energy stored in chemical bonds (e.g., in sugars)^7^ is converted to a gradient through the process of oxidative phosphorylation.^8^ Transmembrane gradients provide a convenient store of free energy that can be released on demand in a controlled and precise manner.^1,9,10^ Biology employs phospholipid bilayers for compartmentalization, and has evolved exquisite active transport mechanisms to pump protons, electrons, ions, and small molecules across these lipid bilayer membranes.^1–4^

The scarcity of primary active transport systems capable of driving and sustaining gradients across lipid bilayers in artificial nanotechnology, stands in stark contrast to the ubiquitous role of such processes in biology.^11,12^ While a few examples of both light-driven^13–19^ and chemically fueled^20–22^ active transport have been reported, these have predominantly demonstrated transport across a bulk liquid phase (organic solvent), often in U-tubes (Figure 1A, left).^15–21^ Translating such approaches to lipid bilayer membranes remains a substantial and, as yet, unrealized challenge (Figure 1A). Indeed, most examples of light-driven active transport rely on illuminating opposite sides of the aqueous–organic interfaces with orthogonal wavelengths to selectively change the conformation of a molecule in a spatially defined manner.^16–19^ These approaches cannot be translated to lipid bilayers, where such spatial separation of different wavelengths of light is not possible. Instead, most transport across lipid membranes in large unilamellar vesicles (LUVs) has focused on the passive transport of ions/molecules down their concentration gradients, facilitated by synthetic channels and carriers.^23–26^ While stimuli can sometimes regulate the activity of synthetic carriers and channels, the transport that they mediate generally remains passive, i.e., movement down a concentration gradient.^27,28^ Despite extensive interest in controlling ion transport and the increased understanding brought about by small-molecule mechanically interlocked pumps,^12,29–39^ the field of membrane-bound molecular machines remains in its infancy.^40–42^ Only a single system capable of artificial active transport across biomimetic phospholipid bilayers has been reported, where light-driven radical charge separation and recombination was exploited for directional uphill transport of H^+^ or Ca^2+^.^13,14^

**Figure 1:**
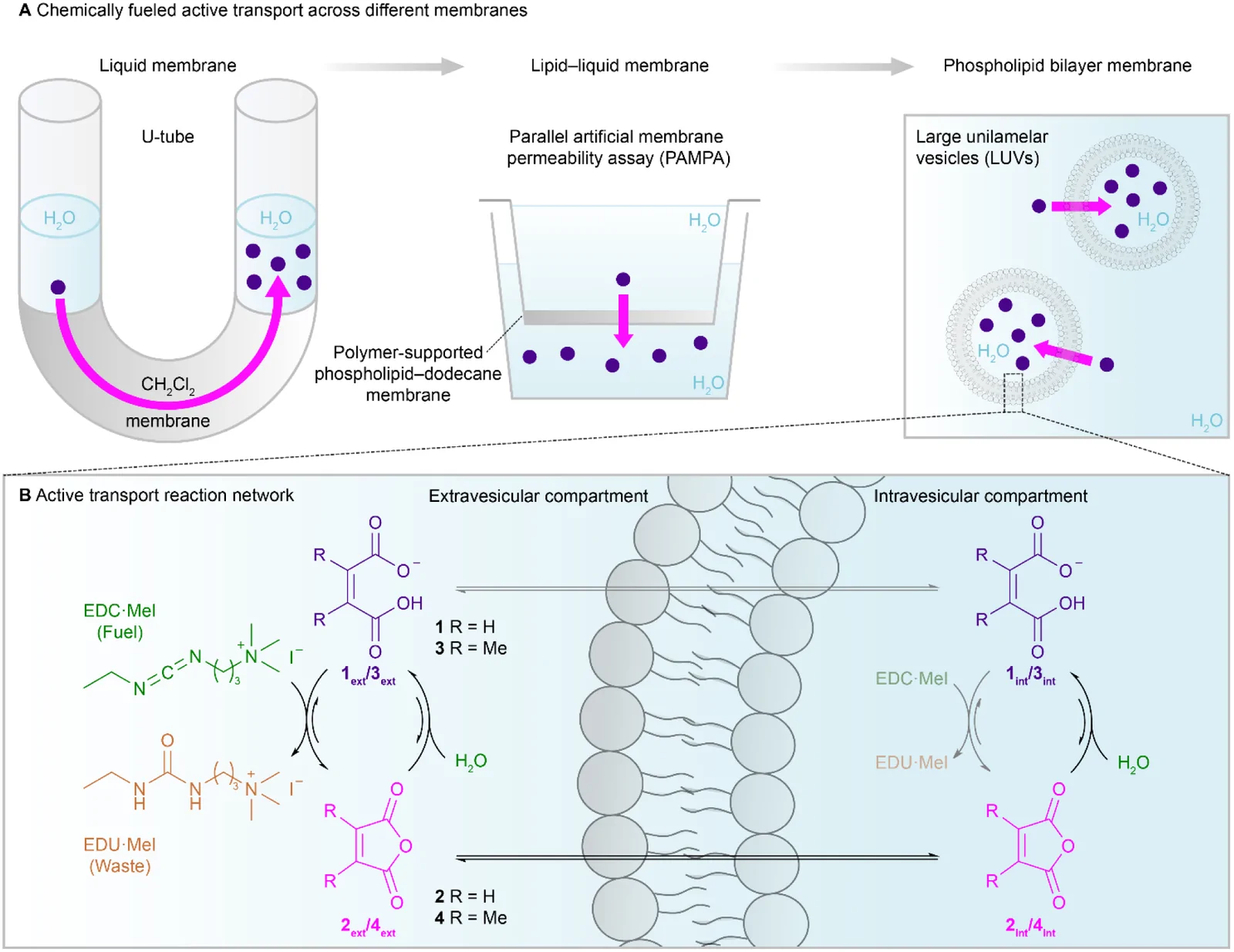
Chemically fueled active transport. (A) Hydrophobic membrane models for active transport. U-tube setup (left) representing the current state of the art^20,21^ with a liquid membrane separating aqueous compartments. The parallel artificial membrane permeability assay (PAMPA) set up (middle) with a polymer-supported lipid–liquid membrane composed of phospholipids in dodecane. The hybrid lipid–liquid membrane separates aqueous compartments. Large unilamellar vesicles (right) provide a biomimetic phospholipid bilayer, with extravesicular and intravesicular aqueous compartments. (B) The transmembrane reaction network for the chemically fueled active transport of a diacid cargo across a lipid bilayer. The diacid cargo (purple) catalyzes the hydration of a carbodiimide fuel (green), generating urea waste (orange), and transiently forming an anhydride (pink). By localizing the fuel to the extravesicular solution, kinetic asymmetry is introduced, pumping the cargo into the intravesicular solution through a chemically fueled active transport process. The faded arrows indicate slow processes. All transitions are shown as reversible in line with the principle of microscopic reversibility,^9^ although under the experimental conditions the position of equilibrium of the fuel-to-waste reaction heavily favors carbodiimide hydration.

Here, we report the catalysis-driven^20,38,39,43^ active transport of a molecular cargo across biologically derived phospholipid bilayer membranes, transducing free energy from a chemical fuel^10,44,45^ to pump a transmembrane gradient of a diacid cargo (Figure 1). We discuss the integration of such an active transport system with phospholipid bilayer membranes, detailing the experimental considerations of moving from fully liquid membranes (U-tube experiments),^20^ to hybrid lipid–liquid membranes^46^ (Figure 2), to lipid-bilayer LUVs (Figures 3–5). Quantitative ^1^H nuclear magnetic resonance (NMR) spectroscopy measurements of LUV suspensions reveal the generation of substantial transmembrane molecular gradients upon chemical fueling. Kinetic quantification of the underlying transmembrane reaction network that drives active transport provides excellent agreement with the observed experimental data, allowing us to establish a range of key performance indicators that describe molecular pumps. Kinetic fluorescence assays^47,48^ corroborate the NMR spectroscopy observations and demonstrate that, as well as performing active transport of the molecular cargo, the system also generates a transmembrane H^+^ gradient (Figure 4). Finally, we harness this H^+^ gradient for the secondary active transport^49^ of cationic and anionic species through symport/antiport mechanisms^50–52^ (Figure 5), thus establishing a cascade whereby chemical energy is transduced to generate a transmembrane gradient, which subsequently drives other nonequilibrium transport processes.

**Figure 2:**
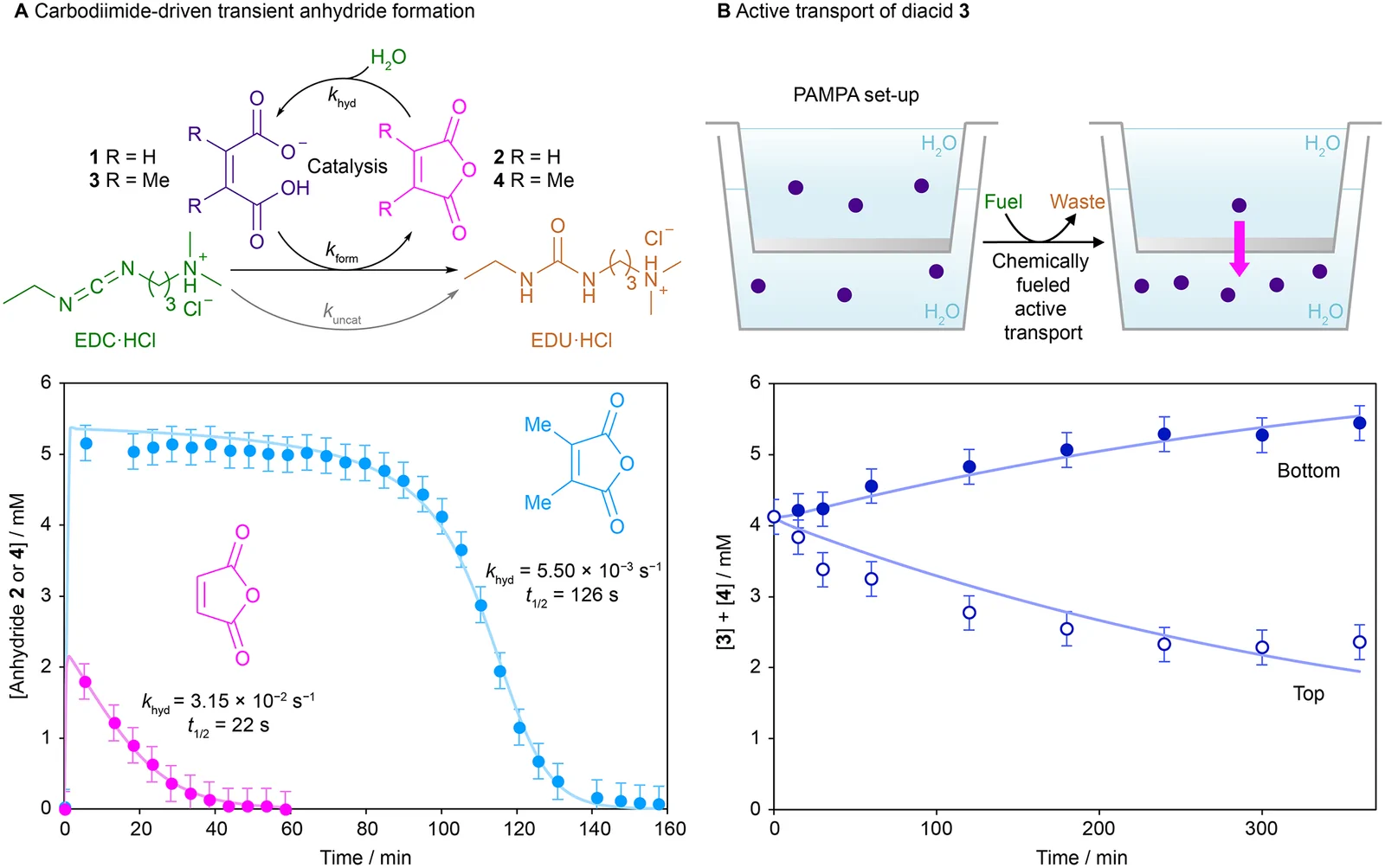
Active transport in lipid–liquid membranes. (A) Transient concentrations of maleic anhydride **2** (pink) and 2,3-dimethylmaleic anhydride **4** (blue) monitored by ^1^H NMR spectroscopy (D_2_O/H_2_O 1:9, *v*/*v*, 298 K, 400 MHz) upon treating maleic acid **1** or 2,3-dimethylmaleic acid **3** with EDC·HCl ([**1** or **3**]_0_ = 5 mM, [EDC·HCl]_0_ = 100 mM, [MES] = 250 mM, p­(H,D)_obs_ 6.0). Solid lines correspond to kinetic modeling fitted to the experimental data (Supporting Information, Section S2.3). Error bars represent standard 4% integral errors. (B) Active transport of **3** in PAMPA. Change in concentration of diacid **3** (and/or its anhydride form **4**) in the PAMPA setup monitored by HPLC ([**3_top_**]_0_ = [**3_bottom_**]_0_ = 4.12 mM, [MES] = 250 mM pH_obs_ 6.0; volume_top_ = 200 μL; volume_bottom_ = 300 μL), upon fueling the top compartment with EDC·HCl (400 mM) at *t* = 0; error bars represent the standard deviation of two independent experiments. We note that hydrolysis of **4** upon HPLC analysis prevents distinguishing separate concentrations for **3** and **4**. Solid lines correspond to kinetic modeling fitted to the experimental data (Supporting Information, Section S2.4).

## Results and Discussion

### From Liquid to Lipid Membranes

Demonstrating active transport across biomimetic LUVs requires the fulfillment of several conditions. (1) The compatibility of the fueling system with phospholipid vesicles should be demonstrated. (2) A means of detecting and quantifying cargo molecules on either side of the membrane should be established. (3) Any background (passive) transport should be assessed and accounted for. (4) Quantitative measurements should be made to rationalize the reaction network.

We previously demonstrated catalysis-driven active transport of a maleic acid cargo **1** across a liquid (CH_2_Cl_2_) membrane in a U-tube set up (Figure 1A).^20^ Considering the general transmembrane reaction network (Figure 1B), carbodiimide-driven anhydride formation^53–55^ converts charged diacid **1** to corresponding maleic anhydride **2** (**1** + carbodiimide → **2** + urea), which can diffuse through the hydrophobic membrane (**2**_**ext** _⇋ **2**_**int**_). Hydrolysis completes the catalytic cycle to regenerate diacid **1** (**2** + H_2_O → **1**), completing the active transport pathway (**1**_**ext** _→ **2**_**ext** _⇋ **2**_**int**_ → **1**_**int**_). In the previous U-tube experiments,^20^ by localizing the carbodiimide fuel to a single side of the liquid membrane, maleic acid cargo **1** was pumped almost quantitatively to the opposite side. We reasoned that, in principle, this catalysis-driven active transport might be amenable to direct translation to a phospholipid bilayer (Figure 1A), allowing for chemically fueled active transport between the intra- and extravesicular compartments (Figure 1B). However, while both a layer of chlorinated solvent and a phospholipid bilayer are hydrophobic membranes that separate aqueous compartments, there are several key differences between the two. For example, the nature of the phospholipid–water interface is governed by the structure of the charged phosphatidylcholine head groups.^56,57^ Reactivity of, or binding to, the head groups may occur,^58^ while the high-density of charged residues at the phase boundary presents a complex barrier to mass transport, not present in simple liquid–liquid phase boundaries.^57^ The hydrophobic region of the membrane is also complex. Factors such as degree of unsaturation or cholesterol content will affect membrane fluidity and viscosity,^59^ and thus might substantially impact transmembrane transport. Passive leakage of the cargo across the narrow lipid bilayer membrane, absent from the bulk liquid membranes in U-tubes, is also more likely to occur.^60^ The roles of convection and diffusion will differ, with the rate of stirring in U-tubes having been crucial to achieving efficient transport.^20,21^ A final practical consideration relates to detection. Vesicle-based experiments present an interior compartment that cannot be easily interrogated by ex situ techniques (e.g., high-performance liquid chromatography (HPLC) or mass spectrometry) without prior purification (e.g., size exclusion chromatography), additionally, techniques for in situ analysis must be sufficiently sensitive to overcome the large difference in volume between the intra- and extravesicular solutions.

### Active Transport with Hybrid Lipid–Liquid Membranes

We chose to employ hybrid lipid–liquid artificial membranes^46^ as a stepping-stone moving from fully liquid, to lipid vesicles (Figure 1B, middle). The parallel artificial membrane permeability assay (PAMPA)^61,62^ is designed to create a membrane between two aqueous compartments, allowing for direct sampling and subsequent analysis by HPLC. The membrane comprises a mixture of dodecane and phospholipids adsorbed onto a polymer support (see Supporting Information, Section S2.1). Thus, while still fundamentally a liquid (dodecane) membrane, the experimental setup introduces a lipid interface,^56,57^ allowing evaluation of the influence of charged and potentially reactive phosphatidylcholine lipid headgroups on chemically fueled active transport. A membrane of 1,2-dioleoyl-*sn*-glycero-3-phosphocholine (DOPC) was created in the PAMPA setup.^61,62^ DOPC was dissolved in dodecane (40 mg mL^–1^) and an aliquot (5 μL) was used to coat the membrane support (see Supporting Information, Section S2.1). Aqueous (2-(*N*-morpholino)­ethanesulfonic acid) (MES) buffer (250 mM, pH 6.00) was used in both top and bottom compartments. Direct sampling of both compartments thus allowed quantification of the concentrations of the diacid cargo by HPLC. To assess the passive transport (leakage) of the diacid cargo and the carbodiimide fuel, maleic acid **1** (5 mM) or carbodiimide fuel 1-(3-(dimethylamino)­propyl)-3-ethylcarbodiimide hydrochloride (EDC·HCl, 100 mM) were dissolved in the top compartment (see Supporting Information, Section S2.2.3). After 18 h, the bottom compartment showed negligible signs of passive transport of either species (<1%), consistent with the compounds’ lack of solubility in the hybrid membrane.

Active transport experiments were investigated starting from equimolar concentrations of maleic acid **1** on either side of the lipid–liquid membrane (membrane = 5 μL aliquot of 40 mg mL^–1^ DOPC in dodecane; top compartment = bottom compartment, [**1**]_0_ = 5 mM, [MES buffer] = 250 mM, pH_obs_ 6.0). Fueling the top compartment with EDC·HCl (100 mM) resulted in successful active transport of maleic acid **1** from the top to the bottom compartment, producing a modest gradient of [**1**_**top**_] – [**1**_**bottom**_] = 0.52 mM (see Supporting Information, Section S2.2.4). The relatively small gradient observed is likely due to fast hydrolysis of **2** (half-life for hydrolysis of **2** = 22 s at pH 6.0, Figure 2A) occurring before the anhydride reaches the membrane, i.e., a futile cycle.^38,39^ This was confirmed by adding maleic anhydride **2** (8 mM) to only the top compartment. A negligible amount of maleic acid **1** appeared in the bottom compartment (<0.1 mM) indicating hydrolysis of **2** is faster than its passive diffusion across the membrane (see Supporting Information, Section S2.3.3). Increasing the fuel concentration, [EDC·HCl]_0_ = 400 mM, results in a larger gradient [**1**_**top**_] – [**1**_**bottom**_] = 1.01 mM (see Supporting Information, Section S2.2.4) but does not solve the fundamental futile cycle issue. Thus, we screened alternative diacids to identify longer-lived anhydrides, i.e., with smaller rate constants for hydrolysis (see Supporting Information, Section S2.3.1). 2,3-Dimethylmaleic acid **3** formed 2,3-dimethylmaleic anhydride **4** upon fueling, which had a lifetime around six-times longer than **2** (half-life for hydrolysis of **4** = 126 s, Figure 2A). Accordingly, anhydride **4** (8 mM) was added only to the top compartment. The appearance of ∼0.2 mM acid **3** in the bottom compartment indicates that some passive diffusion of anhydride **4** occurs prior to hydrolysis, indicating its increased suitability for active transport (see Supporting Information, Section S2.3.3). An equimolar concentration of diacid **3** was placed either side of the membrane in the PAMPA setup (membrane = 5 μL aliquot of 40 mg mL^–1^ DOPC in dodecane); concentration in top compartment (volume = 200 μL) = bottom compartment (volume = 300 μL) = [**3**_**top**_]_0_ = [**3**_**bottom**_]_0_ = 4.12 mM, [MES buffer] = 250 mM, pH_obs_ 6.0). Fueling either the top or bottom compartment with EDC·HCl (400 mM) resulted in generation of a transmembrane gradient of ∼4.0 mM (monitored by HPLC, Figure 2B, Supporting Information, Section S2.2.4). Since the volume of the top compartment (200 μL) is smaller than the volume of the bottom compartment (300 μL), the concentration changes for the cargo are not specular across the two compartments, i.e., the larger decrease in the top compartment is associated with a smaller increase in the bottom compartment. Kinetic modeling (Figure 2B, solid lines, Supporting Information, Section S2.4) describes the experimentally observed active transport with a normalized root-mean-square error of 5% (see Supporting Information, Section S2.4) once a factor was introduced to account for the slow diffusion (relative to hydrolysis). Notably, only anhydrides formed within ∼0.5 mm from the membrane can diffuse into the hydrophobic domain before hydrolyzing (see Supporting Information, Section S2.4). Such a limitation might help explain why biology has evolved to rely on complex membrane-bound pumps that activate cargos directly at the membrane.^4,8^ The nonequilibrium molecular concentrations achieved in this active transport experiment correspond to a stored free energy of 1.08 J L^–1^ (see Supporting Information, Section S2.5).^20,63^

These results convincingly demonstrate active transport across hybrid lipid–liquid membranes and show no degradation of the diacid cargo, confirming that phospholipids neither react with the cargo, nor hinder the diffusion of the anhydride between the phases. The lower degree of active transport with faster hydrolyzing anhydride **2** indicates that slow diffusion of the anhydride toward the membrane relative to its hydrolysis can introduce futile cycles,^38,39^ limiting efficiency. Decreasing the average distance from any point in solution to the membrane, e.g., by having well-dispersed LUVs, should mitigate this issue.

### Chemically Fueled Active Transport in LUVs

A well-dispersed suspension containing LUVs offers the potential for in situ measurement of both the extra- and intravesicular solutions by quantitative ^1^H NMR spectroscopy (Figure 3A, Supporting Information, Section S3), provided resonances for internal and external species can be differentiated.^64^ The chemical shift of maleic acid **1** is highly pH sensitive, especially in between the p*K*_a_ values of both acids (p*K*_a_ = 1.9 and 6.2). The ^1^H NMR chemical shift of **1** was measured at a range of different pH values relative to sodium trimethylsilylpropanesulfonate (DSS) as a reference standard (D_2_O, 500 MHz, 298 K, [**1**] = 5 mM, [NaCl] = 200 mM, [MES buffer] = 100 mM, [DSS] = 2.5 mM, see Supporting Information, Section S3.2). This resulted in a sigmoidal pH–chemical shift profile, demonstrating that pH differences of ∼0.3 units should be sufficient for differentiating extravesicular (**1_ext_**) and intravesicular (**1**_**int**_) cargo species.^64^

We initially assessed the passive membrane permeability (i.e., the leakage rate) of maleic acid **1**.^60,64^ DOPC vesicles containing **1**, MES buffer, and 2,6-naphthalenedisulfonic acid disodium salt (NDA) as an ^1^H NMR standard for the intravesicular solution, were prepared by extrusion through a 200 nm polycarbonate membrane. Purification of the vesicles was achieved by size exclusion chromatography (Supporting Information Section S3.2), eluting with an MES buffered solution containing DSS, intended for use as a standard for the extravesicular solution. The resultant vesicles had the following composition: DOPC = 12 mM, intravesicular solution: D_2_O, [**1**_**int**_]_0_ = 38 mM, [NDA] = 19 mM, [NaCl] = 200 mM, [MES buffer] = 100 mM, pH_obs_ 6.0; extravesicular solution: D_2_O, [**1**_**ext**_]_0_ = 0 mM, [DSS] = 2.5 mM, [NaCl] = 200 mM, [MES buffer] = 100 mM, pH_obs_ 5.6 (Supporting Information, Section S3.2). Immediately following size exclusion chromatography, the suspensions of LUVs were monitored by ^1^H NMR spectroscopy (Supporting Information, Section S3.2.3). The peak corresponding to internal maleic acid **1** was clearly visible, and integration by peak fitting against the NDA interior standard indicated a concentration of ∼31 mM in the first spectrum, obtained ∼19 min after purification (Supporting Information, Section S3.2.3). A second resonance corresponding to **1** at pH 5.6, the pH of the extravesicular solution, was also observed at a concentration of 0.2 mM relative to the extravesicular DSS standard. We note that the relative integrals of the intravesicular NDA standard and the extravesicular DSS standard indicate that the intra- and extravesicular volumes correspond to 4% and 96% of the total solution respectively, in good agreement with first-principles calculations (Supporting Information, Section S3.6). Due to this 24× difference in volumes, the ^1^H NMR integrals of species in the intravesicular solution are relatively small despite being at a higher concentration. Over time, the integral of the peak of **1_ext_** increased, while **1**_**int**_ decreased, indicating leakage of **1**, reaching a pseudo-equilibrium after ∼4 h. We note that due to the pH difference this pseudo-equilibrium ratio of maleic acid was 14 mM inside and 0.8 mM outside the vesicles. Transmembrane diffusion of **1** nonetheless continues at pseudo-equilibrium, slowly equilibrating the pH, and thus the concentration of **1** (i.e., once the extra- and intravesicular pH equilibrate, [**1_ext_**] will be equal to [**1**_**int**_]). The change in concentrations of **1**_**ext**_ and **1**_**int**_ over time were fitted to give first order rate constants for leakage (Supporting Information, Section S3.7.4). As expected, the results show a lower rate of leakage at higher pH, due to greater proportion of **1** in its dianionic form.

Having reached a pseudo-equilibrium concentration of **1**, we next fueled the extravesicular solution of the vesicles with EDC·MeI ([EDC·MeI]_0_ = 100 mM added to DOPC LUVs = 12 mM; intravesicular solution: [**1**_**int**_]_0_ = 11 mM, [NDA] = 19 mM, [NaCl] = 200 mM, [MES buffer] = 100 mM, pH_obs_ 6.0; extravesicular solution: [**1_ext_**]_0_ = 1.0 mM, [DSS] = 2.5 mM, [NaCl] = 200 mM, [MES buffer] = 100 mM, pH_obs_ 5.6, Figure 3B, see Supporting Information, Section S3.5). The EDC·MeI tertiary ammonium fuel shows negligible leakage compared to EDC·HCl due to both the permanent ammonium charge and its inability to form the cyclic guanidinium.^65^ Following fueling, immediate formation of maleic anhydride **2** was observed (Figure 3B and C, resonance at 7.24 ppm). The concentration of extravesicular maleic acid **1_ext_** decreased, while the intravesicular concentration (**1**_**int**_) increased, reaching a maximum of ∼18 mM after ∼35 min. At this stage, the rate of leakage surpasses the rate of active transport, resulting in dissipation of the transmembrane gradient by equilibration of the intra- and extravesicular concentrations of **1**. We note that the observed increase of ∼7 mM of [**1**_**int**_] during active transport is associated with a corresponding decrease of only ∼0.6 mM in [**1_ext_**], with the unequal concentrations reflecting the large difference in intravesicular and extravesicular volumes. Repeat experiments showed consistent behavior (Supporting Information, Section S3.5).

We determined the kinetics for diacid **1**-catalyzed hydration of EDC·MeI in isolated ^1^H NMR spectroscopy experiments at the pH of both the intra- and extravesicular solutions separately ([**1**] = 5 mM, [EDC·MeI]_0_ = 50 mM, [DSS] = 2.5 mM, [NaCl] = 200 mM, [MES buffer] = 100 mM, pH_obs_ 5.6 or 6.0, Supporting Information Section S3.7.3). This allowed quantification of rate constants for all chemical (vertical) transformations in the transmembrane reaction network for active transport of **1** (Figure 1B). We had previously determined rate constants for leakage of **1** (Supporting Information, Section S3.7.4), and the diffusion of anhydride **2** was assumed to be fast, due to its relative hydrophobicity and the likelihood that it would always be formed close to a bilayer membrane, given the relatively small intervesicle distances in the experiment ([DOPC] = 12 mM, 200 nm vesicles, average intervesicle distance = 340 nm, Supporting Information, Section S3.6.3). Thus, we simulated the active transport of **1** under EDC·MeI-fueled conditions based on rate constants derived from these independent experiments. Overlaying the simulated time-course data with experimentally determined concentration showed general agreement, which could be further refined by refitting only the leakage rates (which show a large pH sensitivity, see Supporting Information Section S3.7.4) to account for minor pH differences under real experimental conditions (Figure 3C, Supporting Information, Section S3.7.6 c.f. Section S3.7.5). The excellent agreement of the model prediction with the experimental data (normalized root-mean-square error of 7%) indicates the validity of the model, thus confirming the mechanism of active transport.

**Figure 3:**
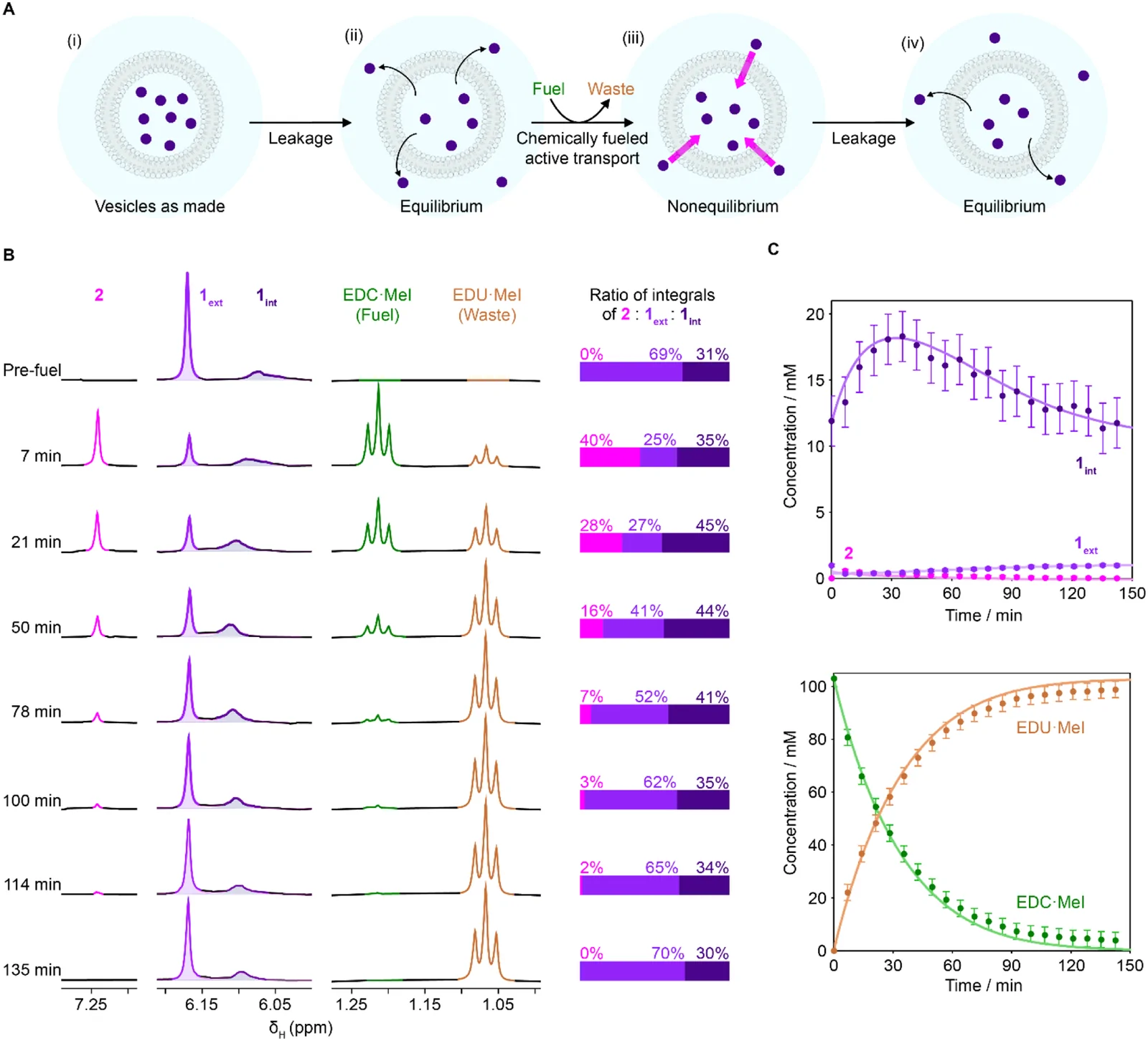
In situ monitoring of active transport in LUVs by ^1^H NMR spectroscopy. (A) Schematic representation of experiments (Supporting Information, Section S3.5). (i) Vesicles (200 nm) containing intravesicular **1** are prepared by extrusion. (ii) Leakage of the acid occurs to reach a pseudo-equilibrium state. (iii) Chemically fueled active transport is initiated by addition of EDC·MeI (100 mM) to the extravesicular solution, pumping cargo **1_ext_** into the vesicles. (iv) Once the fuel is depleted, **1_int_** slowly leaks out to re-equilibrate. (B) Partial ^1^H NMR (D_2_O, 500 MHz, 298 K, see Supporting Information, Section S3.1) showing the change in concentration of **1_ext_**, **1_int_**, **2** EDC·MeI and EDU·MeI upon fueling a suspension of pseudo-equilibrated vesicles (DOPC = 12 mM, [**1_int_**]_0_ = 11 mM, [NDA] = 19 mM, [NaCl] = 200 mM, [MES buffer] = 100 mM, pH_obs_ 6.0; extravesicular solution: [**1_ext_**]_0_ = 1.0 mM, [EDC·MeI]_0_ = 100 mM, [DSS] = 2.5 mM, [NaCl] = 200 mM, [MES buffer] = 100 mM, pH_obs_ 5.6). The ethyl −CH_3_ resonance is shown for the fuel and waste. The resonances for **1_ext_** and **1_int_** were fitted to a Lorentzian–Gaussian function (fitted peak shown by shading) to deconvolute any peak overlap (Supporting Information, Section S3.2.3). The signal for **1_int_** is smaller than **1_ext_** yet refers to a greater concentration due to the 24× difference between the extravesicular and intravesicular volumes. The region 7.25–6.05 ppm is scaled vertically 240× compared to the region 1.25–1.05 ppm. Bars (right) correspond to the relative integrals of **2**, **1_ext_** and **1_int_** over time. (C) Kinetic plots showing the concentration of **1_ext_**, **1**_**int**_, and **2** (top) and EDC·MeI and EDU·MeI (bottom) over time. Solid lines show an overlaid kinetic model based on independently measured rate constants (Supporting Information, Section S3.7). Error bars represent standard integral errors of 5% of the total concentration of **1_ext_**, **1_int_** and **2** (top graph) and for 3% of the total concentration of EDC·MeI and EDU·MeI (bottom graph).

### Key Performance Indicators of the Transmembrane Pump

Since EDC·MeI fuel is localized to the extravesicular solution, the rate of anhydride formation in the intravesicular solution (**1**_**int**_ → **2**_**int**_) is zero, and therefore the kinetic asymmetry (*q*,^43^ or F_C–H_ ^66^) of the cycle is effectively infinite. However, the leakage of **1** constitutes a slip pathway for the pump mechanism,^43,66,67^ inherently limiting the gradient that the pump can achieve. Application of the nonequilibrium pumping equation^68–70^ indicates a theoretical maximum amplification of the [**1**_**int**_]/[**1_ext_**] ratio of 7.0× ([**1**_**int**_]:[**1_ext_**] = 85:1) under the initial fueling conditions ([EDC·MeI] chemostatted at 103 mM, Supporting Information Section S4). Despite this, the experimentally observed transmembrane molecular gradient of **1** is more modest with a maximum concentration difference of [**1**_**int**_] – [**1_ext_**] = 18.2 mM (vs 10.1 mM at pseudo-equilibrium, Figure 3, values taken from kinetic model, Supplementary Section S3.7), constituting an amplification of the initial [**1**_**int**_]/[**1_ext_**] ratio of 3.2× ([**1**_**int**_]:[**1_ext_**] = 39:1, vs a theoretical maximum amplification of 7.0×). The difference arises from the fact that in the experiment, the fuel and waste concentrations are not chemostatted, and the system approaches the steady state slower than EDC is consumed (Supporting Information Section S4).

The performance of the pump can be characterized through several parameters (Supporting Information, Section S4). The pump reaches a maximum intravesicular concentration of [**1**_**int**_] = 18.2 mM after ∼32 min, (relative to an initial pseudo-equilibrium concentration of [**1**_**int**_]_0_ = 11.9 mM. Thus, the average rate of pumping is 4.87 nmol min^–1^ (the number of moles actively transported to the intravesicular solution per unit time). The catalytic efficiency of the pump is 27%, representing the proportion of fuel-to-waste reactions that proceed via the catalyzed pathway. The pumping efficiency is 1.4%, corresponding to the amount of active transport relative to the catalyzed fuel-to-waste reactions. The low efficiency reflects the high rate of leakage, i.e., the prevalence of slip cycles. These values combined give an overall fuel efficiency of 0.4% (coupling efficiency × pumping efficiency), comparable to many artificial small-molecule motors and pumps.^71^ Finally, the maximum stored energy of the transmembrane molecular gradient is 0.9 J L^–1^ (for diacid **1**, Supporting Information, Section S4), similar to the value obtained for the hybrid lipid–liquid membranes (1.08 J L^–1^, for diacid **3**, *vide supra*, the calculated free energy uses an ideal solution approximation and so is independent of the cargo species). While the vesicle-based experiments have reduced futile cycling as compared to the PAMPA experiments, this is offset by the increased slip cycles, resulting in the similar stored energies.

### Catalysis-Driven Generation of Transmembrane Proton Gradients

Maleic acid cargo **1** has two carboxylic acid groups. Consequently, as its concentration increases within a compartment, a corresponding decrease in the pH of that compartment is anticipated. The active transport process does not physically transport (labile) protons across the membrane; only anhydride **2** is physically transported (Figure 1). Hydrolysis of anhydride **2** generates diacid **1**, converting a neutral water molecule into two acidic groups with p*K*_a_ values of 1.9 and 6.2 respectively, and thus the pH of the intravesicular solution decreases, generating a transmembrane proton gradient. Despite the strongly buffered conditions employed above (Figure 3), a downfield shift in the ^1^H NMR resonance of **1**_**int**_ during pumping is clearly visible (Figure 3B), corresponding to a change in pH of up to 0.3 pH units (Supporting Information, Section S3.5.2). This pH change correlates well with the concentration of **1**_**int**_ and shows hysteresis as the concentration increases/decreases due to differences in the rate of leakage of the molecular cargo and the proton gradient (Supporting Information, Section S3.5.2). The generation of proton gradients without physical transport of H^+^ or HO^–^ is reminiscent of Matile’s photogenerated proton gradients,^15^ where photoinduced charge separation drives selective reduction within vesicles, forming a base, which abstracts a proton from the solvent.

To study this phenomenon further in our system, we employed 8-hydroxypyrene-1,3,6-trisulfonate (HPTS) as a ratiometric pH-sensitive fluorophore, allowing for quantification of intravesicular pH.^48^ The use of HPTS allows us to greatly expand the active transport experiments from the narrow range of conditions necessary for ^1^H NMR quantification. Vesicle composition could be altered and lipid concentrations decreased. For fluorescence experiments we predominantly used 1-palmitoyl-2-oleoyl-*sn*-glycero-3-phosphocholine (POPC) lipids rather than DOPC to decrease the fluidity of the bilayer, and thus potentially reduce passive cargo leakage, ensuring more-efficient proton gradient generation (Supporting Information, Section S5). Fluorescence experiments offer further advantages, with their greater sensitivity enabling the use of more dilute vesicle solutions. The concentration of cargo diacid **1** could be increased (50 mM) while maintaining buffer concentration (100 mM), effectively reducing the equivalents of buffer, and thus enabling greater pH changes to be observed. The buffer was changed to 3-(*N*-morpholino)­propanesulfonic acid (MOPS), to allow for efficient buffering at slightly higher pH values to better match the indicator range of HPTS (p*K*_a_ = 7.3, Supporting Information, Section S5.2). In a typical active transport experiment, lumen acidification was determined by recording the change in the HPTS emission (λ_em_ = 510 nm) over time (λ_ex_ = 405 and 460 nm). POPC LUVs (0.1 mM, 2.5 mL, 100 nm; intravesicular solution: [**1**_**int**_ or **3**_**int**_] = 50 mM, [HPTS] = 3 mM, [NaCl] = 200 mM, [MOPS] = 100 mM, pH 6.5) were suspended in extravesicular buffer (extravesicular solution: [**1_ext_** or **3_ext_**] = 50 mM, [NaCl] = 200 mM, [MOPS] = 100 mM, pH 6.5) and allowed to equilibrate in the cuvette for 1 min prior to fueling. EDC·MeI fuel was added as a stock solution (final [EDC·MeI] = 5–20 mM, Supporting Information Section S5.2). After 10 min, excess Triton X-100 detergent (50 μL, 11% in water/dimethyl sulfoxide 7:1 *v*/*v*) was added to lyse the vesicles and end the assay; each assay was repeated in triplicate.

Ratiometric analysis (*R* = 460 nm/405 nm) was performed and the changes in fluorescence intensity were correlated to the local pH upon calibration (Supporting Information, Section S5.2). Upon fueling, the raw fluorescence data shows a reduction in the emission intensity of the basic form of HPTS (λ_ex_ = 460 nm) and simultaneous increase of the emission intensity of the protonated form (λ_ex_ = 405 nm, Supporting Information Section S5.2), thus the normalized fluorescence intensity (*R*/*R*_0_), and intravesicular pH decrease (Figure 4A and B, respectively).

Control experiments substituting EDC·MeI fuel with EDU·MeI waste did not produce any change in fluorescence, indicating that the urea waste does not mediate active transport or passive ion transport, and is thus not involved in lumen acidification (Supporting Information, Section S5.3.1). Further control experiments lacking diacid **1** showed no change in fluorescence (Supporting Information, Section S5.3.1), likewise confirming the negligible leakage of EDC·MeI, in good agreement with ^1^H NMR data (Figure 3). Dynamic light scattering (DLS) measurements taken before, and 10 min after, fuel addition showed the mean LUV diameter remains unchanged over the course of the experiment (Supporting Information, Section S5.3.3). In combination with ^1^H NMR spectroscopy studies and the stable HPTS readings, we can infer that vesicle integrity is maintained during active transport. Varying the lipid composition from POPC to DOPC or adding cholesterol did not affect the rate of active transport (Supporting Information, Section S5.6.1), indicating that diffusion of the anhydride is not rate limiting, in good agreement with the ^1^H NMR data. Together, the experiments demonstrate that the change in fluorescence arises as a direct consequence of the pH gradient generated due to the chemically fueled active transport of diacid cargo **1**. Furthermore, the data show that transport is not dependent on a preapplied pH gradient (as was employed in the NMR experiments, Figure 3).

Active transport experiments with dimethyl maleic acid **3** resulted in generation of smaller proton gradients and faster leakage (Figure 4A, Supporting Information, Section S5.3.4). This behavior is consistent with the increase in lipophilicity of **3** (versus **1**) in opposition to the results obtained in PAMPA (Figure 2B). This difference stems from what feature limits active transport in each experiment. In LUVs, the increased lipophilicity of the cargo drives faster leakage of acid **3**, limiting the extent of the gradient generated, i.e., active transport is limited by slip pathways. In PAMPA, the anhydride must diffuse over large distances to reach the membrane, and thus hydrolysis of shorter-lived of anhydride **2** may occur prior to diffusion, i.e., active transport is limited by futile cycles.

The extent of vesicle lumen acidification can be modulated based on the amount of EDC·MeI fuel added (Figure 4B). Upon fueling with 20 mM EDC·MeI (Figure 4B, purple), the pH ratio reaches a minimum after ∼10 min, with a decrease of ∼0.32 pH units. This change is consistent with an increase in the intravesicular [**1**_**int**_] of 12 mM (Supporting Information, Section S5.4.3). Factoring in the total intravesicular volume (0.72 μL, Supporting Information, Section S5.4.1), this equates to 8.8 nmol of **1** transported. The total amount of fuel present in the external solution is much greater (∼50 μmol), giving a fuel efficiency of ∼0.018%. In comparison to the ^1^H NMR experiments, for fluorescence measurements the vesicles are much less concentrated, and thus the total intravesicular volume is smaller (0.029% vs 3.6%), resulting in negligible concentration changes of the external solution, and thus extremely low overall energy storage densities (<10^–3^ J L^–1^). However, considering solely the intravesicular volume the maximum stored energy of the transmembrane Δ­[**1**] gradient in this experiment is 3.4 J L^–1^ (Supplementary Information, Section S5.4.4) which is aligned with the value obtained from ^1^H NMR experiments Figure 3C). In addition to the free energy stored in the form of the molecular transmembrane gradient, since the active transport also generates a proton gradient, there is an associated transmembrane electrochemical H^+^ potential (Δ*E*_H_^+^), which can be evaluated using the Nernst equation as Δ*E*_H_^+^ = −18.9 mV (Supporting Information, Section S5.4.2). Notably, the associated transmembrane pH gradient of ∼0.32 units is comparable to the 0.4 pH units involved in mitochondrial ATP synthesis,^72^ which motivated us to exploit the gradient as a free energy source, mimicking biology’s ubiquitous use of transmembrane gradients to drive orthogonal processes.

**Figure 4:**
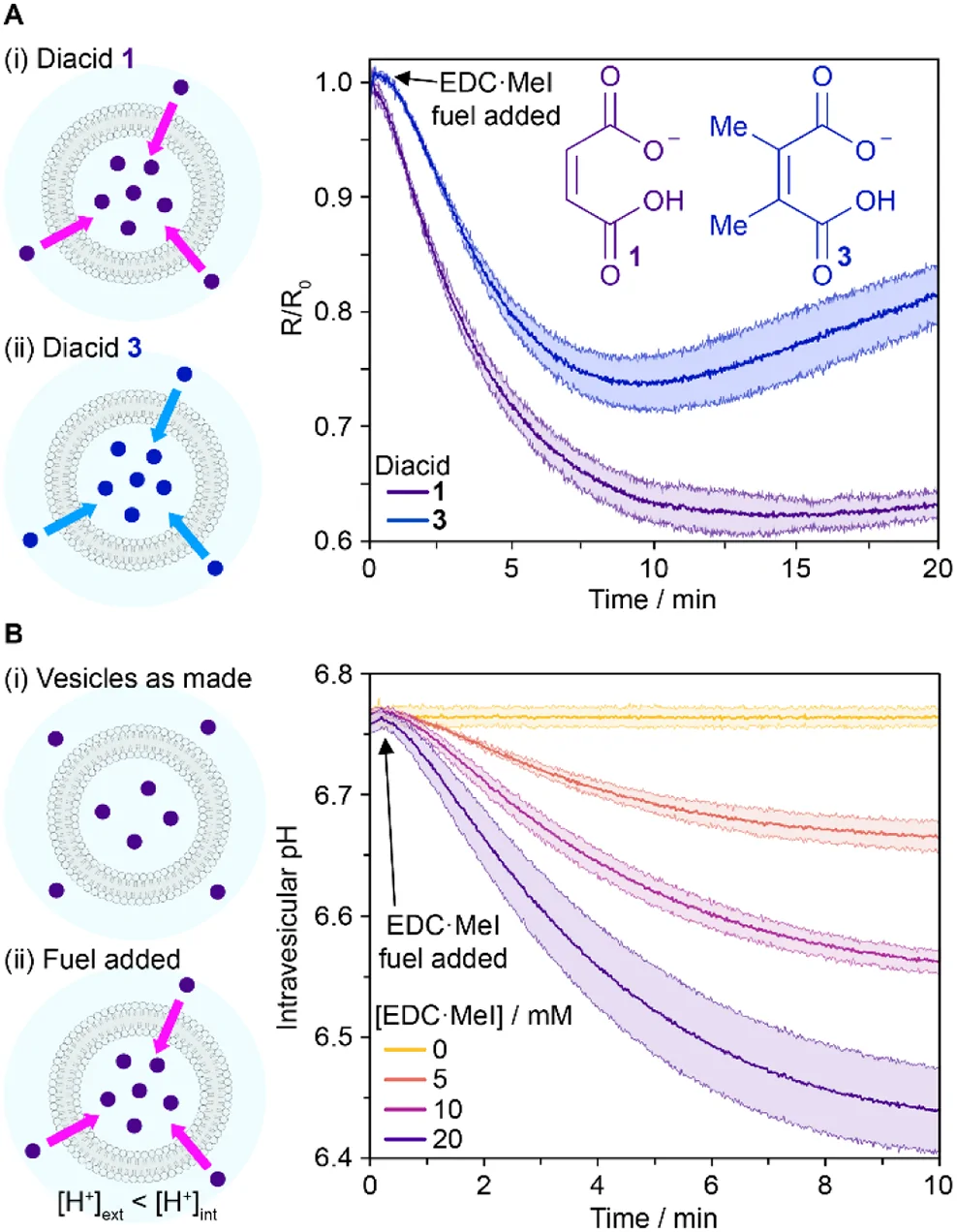
Quantifying active transport-induced acidification by HPTS fluorescence assays. (A) The ratiometric change in intravesicular HPTS emission (λ_em_ = 510 nm; λ_ex1_ = 405 nm, λ_ex2_ = 460 nm) upon addition of fuel ([EDC·MeI]_0_ = 10 mM) to vesicles prepared with (i) diacid **1** (purple) or (ii) **3** (blue) (POPC LUVs 0.1 mM, 2.5 mL, 100 nm, intravesicular solution: [HPTS] = 3 mM, [**1_int_** or **3_int_**]_0_ = 50 mM, [NaCl] = 200 mM, [MOPS buffer] = 100 mM, pH 6.5; extravesicular solution: [**1_ext_** or **3_ext_**]_0_ = 50 mM, [NaCl] = 200 mM, [MOPS buffer] = 100 mM, pH 6.5). (B) Change in intravesicular pH recorded over time upon addition of fuel ([EDC·MeI]_0_ = 0–20 mM) calculated from the ratiometric change in HPTS emission (λ_em_ = 510 nm; λ_ex1_ = 405 nm, λ_ex2_ = 460 nm). (POPC LUVs 0.1 mM, 2.5 mL, 100 nm, intravesicular solution: [HPTS] = 3 mM, [**1_int_**]_0_ = 50 mM, [NaCl] = 200 mM, [MOPS buffer] = 100 mM, pH 6.5; extravesicular solution: [**1_ext_**]_0_ = 50 mM, [NaCl] = 200 mM, [MOPS buffer] = 100 mM, pH 6.5). In both panels, shaded areas of the graph correspond to the standard deviation measured when repeating the assays in triplicate (see Supporting Information, Section S5.3).

### Active Transport Cascades

The proton gradient generated following active transport is dissipated via leakage through the membrane (Figures 3 and 4a). Ionophores bind ions to provide a kinetically accessible, low energy pathway for transmembrane ion passive transport;^24–28^ effectively ionophores catalyze dissipation of a transmembrane gradient.^39^ Just as catalysis of chemical reactions can be harnessed to drive nonequilibrium systems,^38,39,66^ catalysis of the dissipation of a transmembrane gradient can likewise transduce free energy to drive an orthogonal process. Symporting ionophores^22^ (Figure 5A) mediate the cotransport of ions across a membrane in the same direction (e.g., prodigiosin^73^ selectively symports H^+^/Cl^–^), while antiporting ionophores simultaneously transport ions in opposite directions (e.g., monensin^52^ selectively antiports H^+^/Na^+^). Thus, if a gradient of a particular ion is present (e.g., a H^+^ gradient), the symporter/antiporter will dissipate that gradient while driving a second gradient out of equilibrium through a secondary active transport process. Symport has previously been exploited to couple the fatty acid-induced formation of a transmembrane pH gradient to drive a Cl^–^ gradient through H^+^/Cl^–^ symport.^22^

We reasoned that employment of symporters/antiporters to dissipate the gradients formed through our chemically fueled active transport could lead to the formation of active transport cascades.^74^ Free energy can be transduced from the chemical fuel-to-waste reaction to drive primary active transport and form a H^+^ gradient, which might subsequently drive secondary active transport of an orthogonal gradient, in a manner reminiscent of biological transport cascades. To demonstrate this, we generated a H^+^ gradient through active transport of **1** into LUVs (Figure 5), monitoring the pH change by HPTS fluorescence (100 nm POPC vesicles, [POPC] = 0.1 mM, 2.5 mL; intravesicular solution: [**1**_**int**_]_0_ = 50 mM, [HPTS] = 3 mM, [NaCl] = 200 mM, [MOPS] = 100 mM, pH 6.5; extravesicular solution: [**1**_**ext**_]_0_ = 50 mM, [EDC·MeI]_0_ = 10 mM, [HPTS] = 3 mM, [NaCl] = 200 mM, [MOPS] = 100 mM, pH 6.5). Consistent with prior experiments (c.f. Figure 4), active transport drives a decrease in the intravesicular pH ([H^+^]_ext_ < [H^+^]_int_, in this case corresponding to a local (i.e., over the intravesicular volume) stored energy of 1.2 J L^–1^ (Supporting Information, Section S5.5.4). After 10 min, H^+^/Cl^–^ symporter prodigiosin (0.1 nM) was added (Figure 5B), resulting in an increase in intravesicular pH, resulting from the dissipation of the proton gradient ([H^+^]_ext_ < [H^+^]_int_ → [H^+^]_ext_ ≈ [H^+^]_int_) coupled to generation of a Cl^–^ gradient ([Cl^–^]_ext_ = [Cl^–^]_int_ → [Cl^–^]_ext_ > [Cl^–^]_int_); hence, the internal Cl^–^ concentration decreases from 200 mM to 188 mM (see Supporting Information, Section S5.5.4). The energy stored locally in the form of Δ­[Cl^–^] equals 0.91 J L^–1^, indicating an energy transduction efficiency of 58% for this secondary active transport step (see Supplementary Calculations). Similarly, following chemically fueled active transport of **1** into LUVs (in this case corresponding to a locally stored energy of 0.82 J L^–1^, Supporting Information, Section S5.5.4), H^+^/Na^+^ antiporter monensin (10 nM) was added (Figure 5C), resulting in almost immediate dissipation of the pH gradient and secondary active transport of Na^+^ ([H^+^]_ext_ < [H^+^]_int_ → [H^+^]_ext_ ≈ [H^+^]_int_; [Na^+^]_ext_ = [Na^+^]_int_ → [Na^+^]_ext_ < [Na^+^]_int_), corresponding to an increase in the internal Na^+^ concentration from 200 mM to 207 mM (Figure 5C, Supporting Information, Section S5.5.4). In this case, the energy stored locally as [Na^+^] was 0.64 J L^–1^, with an energy transduction efficiency of 57%, very much comparable with that of prodigiosin-facilitated symport. Control experiments performed by adding an ionophore that only facilitates H^+^ uniport (electrogenic protonophore carbonyl cyanide *m*-chlorophenyl hydrazone (CCCP)), resulted in no increased rate of gradient dissipation (Supporting Information, Section S5.5.3), confirming the secondary active transport processes driven by prodigiosin and monensin.^22^ Taken together, these experiments demonstrate how free energy can be absorbed through catalysis of a driving (fuel-to-waste) reaction, and how that stored free energy can subsequently be harnessed to drive further processes.

**Figure 5:**
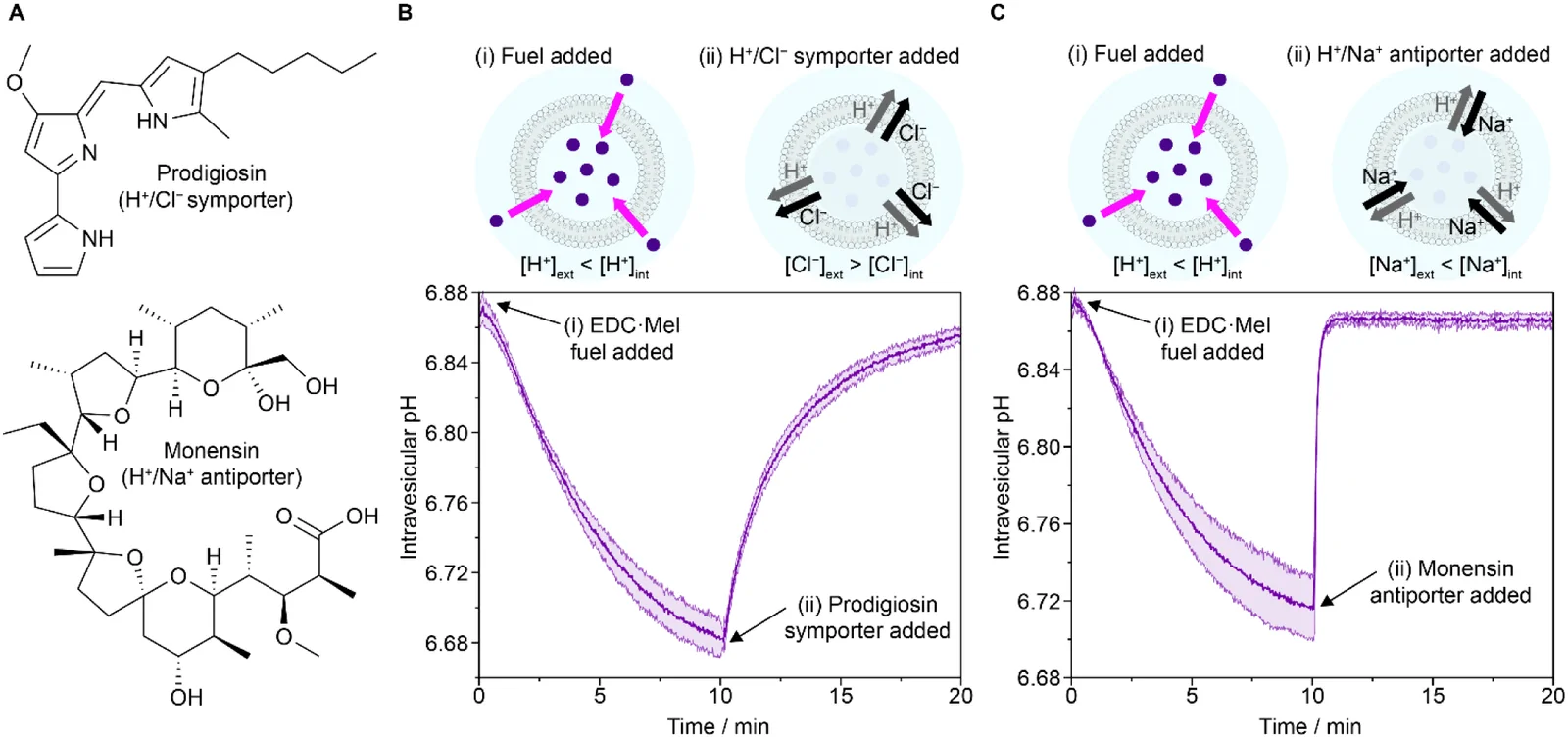
Harnessing pH gradients to drive secondary active transport in chemically fueled active transport cascades. (A) Structures of ionophores used for secondary active transport. (B) Change in intravesicular pH recorded over time upon (i) addition of fuel ([EDC·MeI]_0_ = 10 mM) at *t* = 0, then (ii) addition of H^+^/Cl^–^ symporter prodigiosin at *t* = 10 min (0.0001 mol % with respect to lipid). (C) Change in intravesicular pH recorded over time upon addition of (i) fuel ([EDC·MeI]_0_ = 10 mM) at *t* = 0, then (ii) addition of H^+^/Na^+^ antiporter monensin at *t* = 10 min (0.01 mol % with respect to lipid). In both (B) and (C), POPC LUVs (0.1 mM, 2.5 mL, 100 nm) were prepared with diacid **1** (intravesicular solution: [HPTS] = 3 mM, [**1_int_**]_0_ = 50 mM, [NaCl] = 200 mM, [MOPS buffer] = 100 mM, pH 6.5; extravesicular solution: [**1_ext_**]_0_ = 50 mM, [NaCl] = 200 mM, [MOPS buffer] = 100 mM, pH 6.5) and the local pH was calculated from the ratiometric change in HPTS emission (λ_em_ = 510 nm; λ_ex1_ = 405 nm, λ_ex2_ = 460 nm). In both panels (B) and (C), shaded areas of the graph correspond to the standard deviation measured when repeating the assays in triplicate (see Supporting Information, Section S5.5).

## Conclusions

In summary, we have demonstrated the generation of nonequilibrium transmembrane gradients of a diacid cargo across lipid bilayers through catalysis-driven chemically fueled active transport. Hybrid lipid–liquid membranes served as a stepping-stone to demonstrate the compatibility of the carbodiimide fueling chemistry^10,53–55^ with phospholipids. In situ ^1^H NMR spectroscopy allowed the kinetics of the active transport process in LUVs to be monitored, showing good agreement with modeling, enabling a quantitative description of the underlying mechanism. Application of the nonequilibrium pumping equality and assessment of key performance indicators provides further insight into the activity of the active transport, which compares favorably to a small-molecule chemically fueled pumps based on interlocked architectures.^71^ The transmembrane transport of the diacid cargo is associated with the generation of a transmembrane pH gradient of almost 0.4 pH units, as quantified through both ^1^H NMR spectroscopy and fluorimetry. Finally, we show that the free energy stored in the transmembrane pH gradient can be used to drive the secondary active transport of an orthogonal ion (either Cl^–^ or Na^+^) in an energy transduction cascade. Sequential energy transduction in catalysis-driven artificial machines has previously been limited to mechanical force generation.^75^ As such, our proof-of-concept study provides a fundamental demonstration of how the free energy stored in chemical bonds in a fuel can be harnessed to generate a transmembrane gradient, which can itself provide the free energy source to drive a secondary process. Such energy transduction cascades are ubiquitous to the most sophisticated known nanotechnology, i.e., life, and thus, our findings provide an important milestone as chemists seek to build the artificial nanotechnologies of the future based on this biological blueprint.

## Supplementary Material





## Notes

Vesicle dimension and stored-energy calculations are provided in the Supplementary Calculations spreadsheet. Relevant data reported in this paper will be shared by the corresponding authors upon request (PDF)
Supplementary calculations (XLSX)

## Abbreviations

EDC: 1-(3-(dimethylamino)­propyl)-3-ethylcarbodiimide
DOPC: 1,2-dioleoyl-*sn*-glycero-3-phosphocholine
DSS: sodium trimethylsilylpropanesulfonate
HPLC: high-performance liquid chromatography
HPTS: 8-hydroxypyrene-1,3,6-trisulfonate
LUV: large unilamellar vesicles
MES: 2-(*N*-morpholino)­ethanesulfonic acid
MOPS: 3-(*N*-morpholino)­propanesulfonic acid
NDA: 2,6-naphthalenedisulfonic acid disodium salt
NMR: nuclear magnetic resonance
PAMPA: parallel artificial membrane permeability assay
POPC: 1-palmitoyl-2-oleoyl-*sn*-glycero-3-phosphocholine
