# Supplementary material for: Chemically Fueled Active Transport Cascades in Large Unilamellar Vesicles
Source: J Am Chem Soc. 2026 Aug 3;148(37):39976–87. doi: 10.1021/jacs.6c11418 (PMC13614177; doi:10.1021/jacs.6c11418)
Supplement: Supplementary file 1 [file ja6c11418_si_001.pdf]

Supporting information for:

# Chemically Fueled Active Transport Cascades in Large Unilamellar Vesicles

Willow Baxter,[a,‡] Laura E. Bickerton,[b,‡] Kaiyuan Liang,[b,‡] Emanuele Penocchio,[c] Juan A. Aguilar,[a] Giulio Ragazzon [b,d,*] and Stefan Borsley [a,e,*]

[a]Department of Chemistry, Durham University, Stockton Road, Durham DH1 3LE, UK

[b]Institut de Science et d'Ingénierie Supramoléculaires (ISIS), University of Strasbourg, CNRS & icFRC, UMR 7006, 8 Allée Gaspard Monge, 67000 Strasbourg, France

[c]Department of Chemistry, Northwestern University, Evanston, IL, 60208 USA

[d]Strasbourg Institute for Advanced Studies (USIAS), University of Strasbourg, 5 allée du Général Rouvillois, 67000 Strasbourg, France

[‡]These authors contributed equally

[e]Lead contact

*Correspondence: ragazzon@unistra.fr

**Correspondence: stefan.h.borsley@durham.ac.uk

# Table of Contents


S1. Materials and Methods ..... 3

S1.1. Abbreviations ..... 4

S2. Parallel Artificial Membrane Permeation Assay (PAMPA) ..... 5

S2.1. PAMPA Set-up and Calibration ..... 5

S2.2. Transport of Cargo (PAMPA) ..... 8

S2.3. Isolated Reaction Kinetics ..... 11

S2.4. Full Kinetic Modeling of Multi-Phase Experiments ..... 16

S2.5. Thermodynamic Analysis of Active Transport with PAMPA ..... 19

S3. $^{1}$H NMR Spectroscopy Studies ..... 20

S3.1. Solvent Suppression Protocol for $^{1}$H NMR Studies of LUVs ..... 20

S3.2. Experimental Procedures ..... 21

S3.3. $^{1}$H NMR Quantitation and $T_1$ Relaxation Constants ..... 24

S3.4. pH Calibration Curve ..... 26

S3.5. Active Transport Experiments ..... 27

S3.6. Vesicle Calculations ..... 33

S3.7. Kinetic Modelling of Active Transport ..... 35

Kinetic and Thermodynamic Analysis of Active Transport ..... 45

S3.8. Nonequilibrium Pumping Equation ..... 45

S3.9. Pump Performance Calculations ..... 46

S3.10. Stored Energy Calculations ..... 48

S4. Fluorescence Studies ..... 49

S4.1. General Vesicle Synthesis ..... 49

S4.2. General HPTS Assay and Calibration ..... 50

S4.3. HPTS Active Transport Assays (EDC·MeI fuel) ..... 54

S4.4. Calculations ..... 61

S4.5. Cascade Reactions ..... 63

S4.6. Additional Control Experiments ..... 67

S4.7. HPTS Active Transport Assays (EDC·HCl fuel) ..... 69

S5. References ..... 73

## S1. Materials and Methods

Unless otherwise stated, chemicals including HPLC grade MeCN, 2-morpholinoethanesulfonic acid monohydrate (MES), PBS tablet, maleic acid **1**, maleic anhydride **2**, 2,3-dimethylmaleic anhydride **4**, EDC·MeI, EDC·HCl, formic acid (FA) for HPLC, $D_2O$, the nuclear magnetic resonance (NMR) internal standard sodium trimethylsilylpropanesulfonate (DSS), prodigiosin and monensin (sodium salt) were purchased from Sigma Aldrich, Fisher Scientific, or Fluorochem and used as received. 2,3-dimethylmaleic acid **3** was formed by dissolving **4** in water. Lipids (DOPC, POPC, EYPC) were purchased from Avanti Research either in powder form or as a stock solution in chloroform.

Analytical HPLC ThermoFisher Vanquish coupled to a Vanquish Binary Pump (VF-P10-A), Vanquish Autosampler (VF-A10-A), a Vanquish Column Compartment (VC-C10-A) and a Vanquish Diode Array Detector (VC-D11-A) was used to quantitatively analyze the reaction networks. An acclaim™ Phenyl-1 column (120 Å 3.0 × 150 mm, 3.0 µm pore size) with a guard cartridge was used for analyzing **1** and **3**. Temperature of the column was set to 25 °C and a sample injection volume of 5 µL was used.

Buffers of different pH were prepared using pH meter (fisherbrand, accumet AB315 (Strasbourg) (Mettler Toledo, FiveEasy Plus F20 (Durham)). All indicated p(H,D) values ($p(H,D)_{obs}$) were obtained directly from the pH meter electrode readings, which were taken by immersing the electrode into the aqueous solution.

In Strasbourg, Nuclear Magnetic Resonance (NMR, $^1H$ and $^{13}C$) spectra were collected using Bruker 500 MHz NMR (Magnet: Ascend; Console: Avance Neo; Probe: Cryo-Probe Prodigy X1H 5 mm with $^{31}P < X < ^{15}N$) or Bruker 400MHz NMR (Magnet: Bruker UltraShield Plus; Console: Avance III – 400 MHz; Probe: Cryo-Probe Prodigy X1H 5 mm with $^{31}P < X < ^{15}N$). Chemical shifts (*δ*) are expressed in ppm and were referenced to the residual solvent peak of $D_2O$ at 4.79 ppm. Quantitative NMR data were integrated using MestReNova or TopSpin, and integration was done manually relative to the NMR signal of DSS at 0.02 ppm (internal standard).

In Durham, NMR spectra were recorded on a Varian DD2-500 spectrometer with an operating frequency of 499.53 MHz for $^1H$. $^1H$ chemical shifts are reported in parts per million (ppm). NMR spectra were obtained at 25 °C. Chemical shifts (δ) are expressed in ppm and were referenced to the NMR signal of the internal standard, DSS, at 0.00 ppm. Quantitative NMR data was integrated using MestReNova v16.0.0 and, where specified integration was done using line fitting, and relative to the NMR signal of DSS. All kinetic plots originating from NMR data were obtained using MestReNova and plotted in Microsoft Excel Version 2510.

Large unilamellar vesicle (LUV) preparation was carried out using a LiposoFast Basic extruder from Avestin Inc. (Durham) or an Avanti Mini Extruder (Strasbourg). Size exclusion chromatography was performed using Cytivia PD-10 Columns pre-packed with SephadexTM G-25 M.

The Parallel Artificial Membrane Permeability Assay (PAMPA) kit, including multiwell plates and permeability control compounds, was purchased from BioAssay Systems. UV-vis experiments were carried out on a Cary 60 Spectrophotometer (Varian). Kinetic fluorescence assays were carried out on a Cary Eclipse Fluorescence Spectrophotometer. DLS measurements were conducted on a Zetasizer Lab, Malvern Panalytical instrument.

UV-vis experiments were performed at room temperature (typically 22 ± 2 °C), while NMR spectra were obtained at 25 °C.

Kinetic models and thermodynamic analysis were performed using Python 3.12, Copasi (4.44), and Microsoft Excel.

## S1.1. Abbreviations

**BBO** Broadband observe

**CCCP** Carbonyl cyanide *m*-chlorophenyl hydrazone

**DLS** Dynamic light scattering

**DMSO** Dimethyl sulfoxide

**DOPC** 1,2-Dioleoyl-sn-glycero-3 phosphocholine

**DSS** Sodium 3-(trimethylsilyl)propane-1-sulfonate

**EDC·HCl** 1-ethyl-3-(3-dimethylaminopropyl)carbodiimide hydrochloride

**EDC·MeI** 1-ethyl-3-(3-dimethylaminopropyl)carbodiimide methiodide

**EDU·HCl** 1-(3-(dimethylamino)propyl)-3-ethylurea hydrochloride

**EDU·MeI** 1-(3-(dimethylamino)propyl)-3-ethylurea methiodide

**EYPC** L-a-phosphatidylcholine from egg yolk

**FA** Formic acid

**HPLC** High-performance liquid chromatography

**HPTS** 8-Hydroxypyrene-1,3,6-trisulfonic acid

**LUV** Large unilamellar vesicle

**MeCN** Acetonitrile

**MES** 2-(*N*-morpholino)ethanesulfonic acid

**MOPS** 3-(*N*-morpholino)propanesulfonic acid

**NDA** 2,6-naphthalenedisulfonic acid

**NMR** Nuclear magnetic resonance

**PAMPA** Parallel Artificial Membrane Permeation Assay

**PBS** Phosphate-buffered saline

**POPC** 1-palmitoyl-2-oleoyl-glycero-3-phosphocholine

**PVDF** Polyvinylidene fluoride

# S2. Parallel Artificial Membrane Permeation Assay (PAMPA)

## S2.1. PAMPA Set-up and Calibration

### S2.1.1 General Experimental Setup

The PAMPA kit (Figure S1A) is composed of a bottom plate (B, well volume of 200 µL), a top plate (T, well volume of 300 µL) which has a hydrophobic polyvinylidene fluoride (PVDF) membrane at the bottom (area = 0.24 $cm^2$, pore size = 0.45 µm), a lid to prevent evaporation from the top plate and a third flat plate that the top plate can be placed on during set up to avoid damage to the membranes.

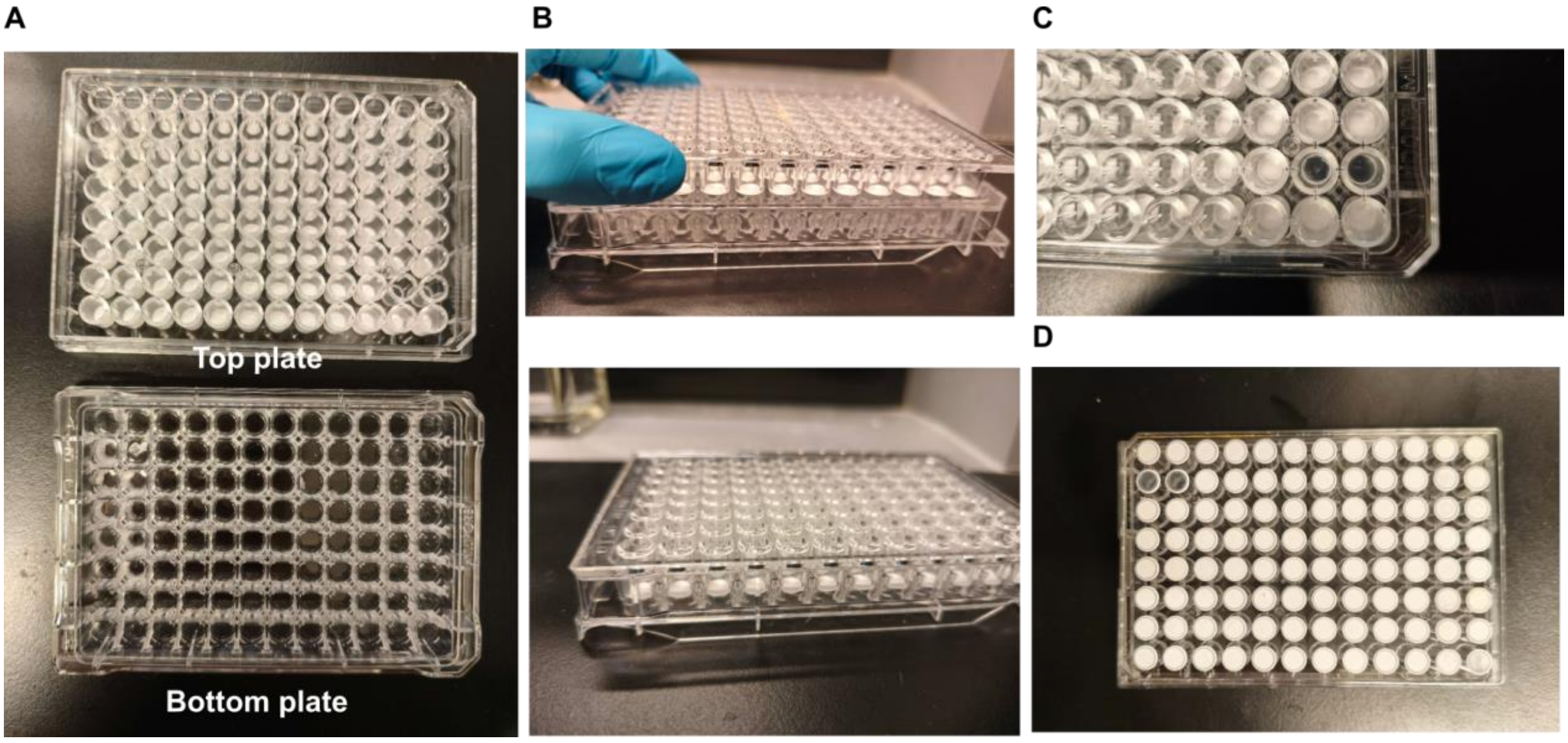


**Figure S1.** The PAMPA set up. **A** Top and bottom plates shown separately. **B** Stacked together. **C** Top plate with two transparent wells which are coated with lipids. **D** Top plate upside down showing the membrane surface.

The membrane used in PAMPA consists of a 4% lipid solution in dodecane. Lipid solutions were prepared as follows: when using lecithin, lecithin (30 mg) was dissolved in dodecane (750 µL); when using DOPC, POPC or EYPC, solutions of these lipids (55 µL, 100 mM in chloroform) were dried under reduced pressure to form thin films, which were subsequently dissolved in dodecane (100 µL) to give a 4% lipid solution.

To apply the lipids to the PVDF support, the top and bottom plates were separated, and a 5 µL aliquot of the lipid solution (4% lipid in dodecane) was added using a Gilson pipette; n.b., the membrane changes from white to translucent (Figure S1C). This step might form a multilayer lipid membrane on the support according to the calculation of membrane area and the amount of lipids.[S1]

For each experiment, buffer was added to the top (200 µL) and bottom (300 µL) compartments before careful reassembly of the kit. Control compounds, or fuel in the case of active transport, were either present in the buffer prior to assembly or added as an aliquot afterwards to the top compartment. To monitor both compartments, the setup was temporarily separated during sampling. Assays were performed in duplicate.

### S2.1.2 PAMPA Calibration

The effective permeability (Pe) of high- (*chloramphenicol*), medium- (*diclofenac*), and low-permeability (*theophylline*) control compounds was measured to validate the experimental setup in accordance with the provided procedure.[S2]

For a typical calibration assay, PBS buffer (10 mM, pH 7.4) was added to the top (190 µL) and bottom (300 µL) compartments. Then the control compound (10 µL, 10 mM stock solution in DMSO; final concentration: 500 µM) was added to the top compartment to initiate the assay. Analogous experiments omitting the control compounds (i.e., DMSO only) were performed in parallel, and the corresponding absorbance values were subtracted from sample measurements to correct for the background.

The assays were incubated for 18 h and then 120 µL was taken from the bottom compartment and diluted to 450 µL for analysis by UV–vis spectroscopy. The peak absorbance wavelengths for the high-, medium-, and low-permeability controls were 280 nm, 270 nm, and 270 nm, respectively.

The peak absorbance for each control compound was used to determine the effective permeability, *Pe* (cm $s^{-1}$) using the following equation:[S2]

$$\mathrm{Pe} = \mathrm{C} \cdot \left(-\ln\left(1 - \frac{\mathrm{OD_B}}{\mathrm{OD_E}}\right)\right) \quad \text{(Equation S1)}$$

Where $OD_B$ is the absorbance of the bottom compartment, $OD_E$ is the absorbance of the control compound expected at equilibrium (corresponding to an equilibrium concentration of 53 µM), and C (cm $s^{-1}$) is a geometric and temporal normalization factor derived from compartment volumes, membrane area, and incubation time and can be calculated with the equation:

$$\mathrm{C} = \frac{\mathrm{V_T\,V_B}}{(\mathrm{V_T} + \mathrm{V_B}) * \mathrm{Area} * \mathrm{time}} \quad \text{(Equation S2)}$$

C = $7.72\times10^{-6}$ cm $s^{-1}$

Here, $V_T$ and $V_B$ are the volumes of the top (200 µL) and bottom (300 µL) compartments respectively, the Area relates to the membrane area (0.24 $cm^2$) and time relates to the incubation time of the assay (18 hour).

All control compounds gave calculated Pe values consistent with the provided values, confirming the function of the PAMPA setup. To optimize the conditions for transport experiments using biologically relevant lipids, a series of membrane conditions were compared (Figure S2). For example, the PVDF solid support only (Figure S2B), dodecane only (Figure S2C), dodecane with different concentrations of lecithin (3.2–4.8%, Figure S2D–F) and DOPC (4% lipid in dodecane, Figure S2G). EYPC and POPC showed low solubility in dodecane and were not studied further.

Comparative experiments were performed using MES buffer (250 mM MES, pH 6.0, Figure S2H & I). In these experiments, control compounds were dissolved directly into the MES buffer without DMSO (top = 500 µM control compound, 250 mM MES & bottom = 250 mM MES).

The permeability of 2,3,4,5,6-pentafluorophenol was also investigated, acting as another high permeability control (top = 500 µM 2,3,4,5,6-pentafluorophenol, 250 mM MES & bottom = 250 mM MES, Figure S2H & I).

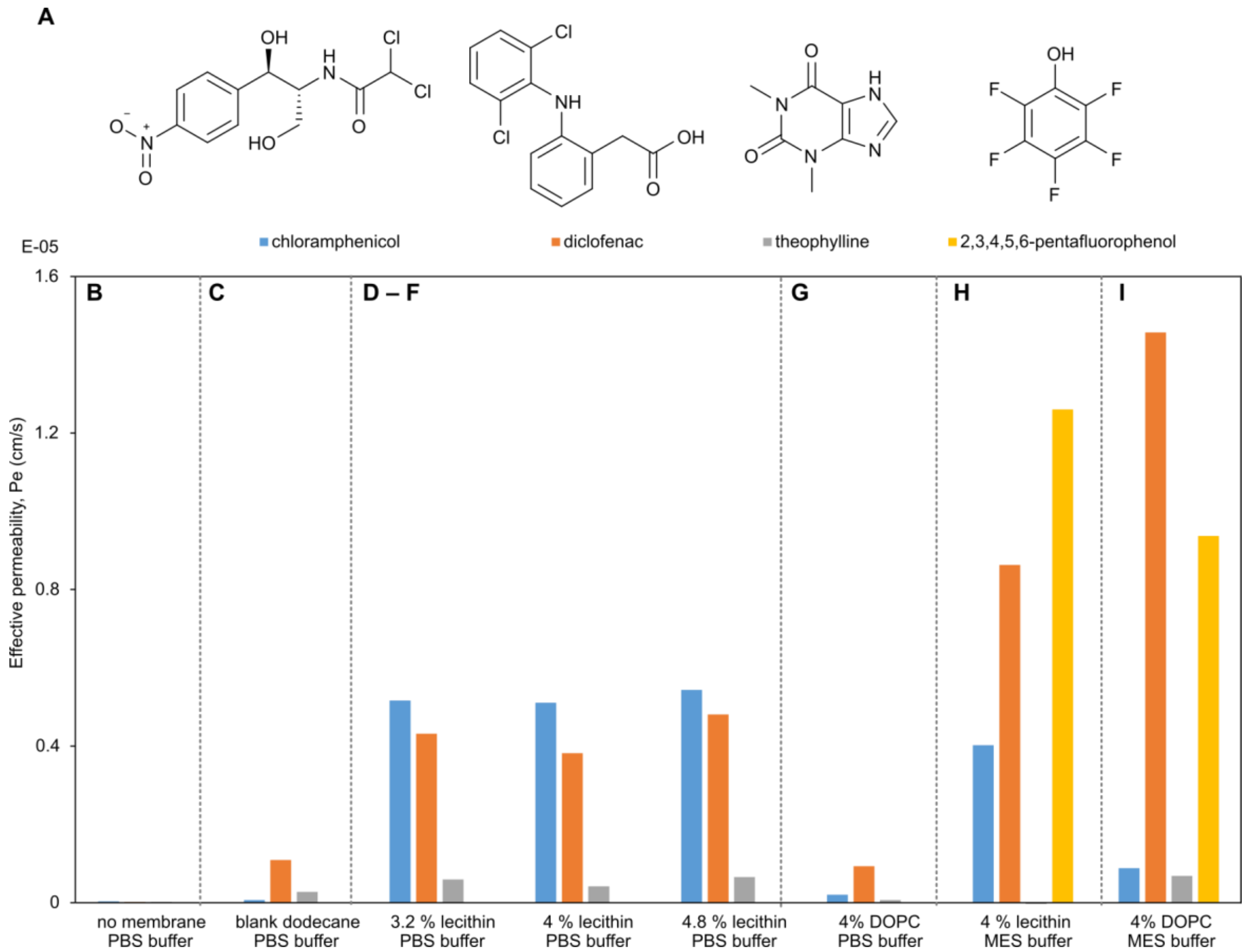


**Figure S2.** PAMPA of control compounds under different conditions: **A** Control compounds; **B** PVDF support only; **C** 5 µL dodecane only; **D–F** 5 µL dodecane with 3.2%, 4%, or 4.8% lecithin; **G** 5 µL of dodecane containing 4% DOPC; **H**, **I** 5 µL dodecane containing 4% lecithin or DOPC. In experiments **B**–**G** the top = 10 mM PBS, pH 7.4 and control compounds added in 10 µL of a DMSO stock solution, final concentration: 500 µM; bottom = 10 mM PBS, pH 7.4. For **H** and **I**, control compounds were dissolved directly in the buffer (top = 500 µM control compound, 250 mM MES buffer, pH 6.0; bottom = 250 mM MES buffer, pH 6.0). In experiment **H** the calculated permeability for low permeability control was less than 0 (Equation S1).

## S2.2. Transport of Cargo (PAMPA)

The transport behavior of molecules **1** – **4** (Figure S3) was investigated using PAMPA.

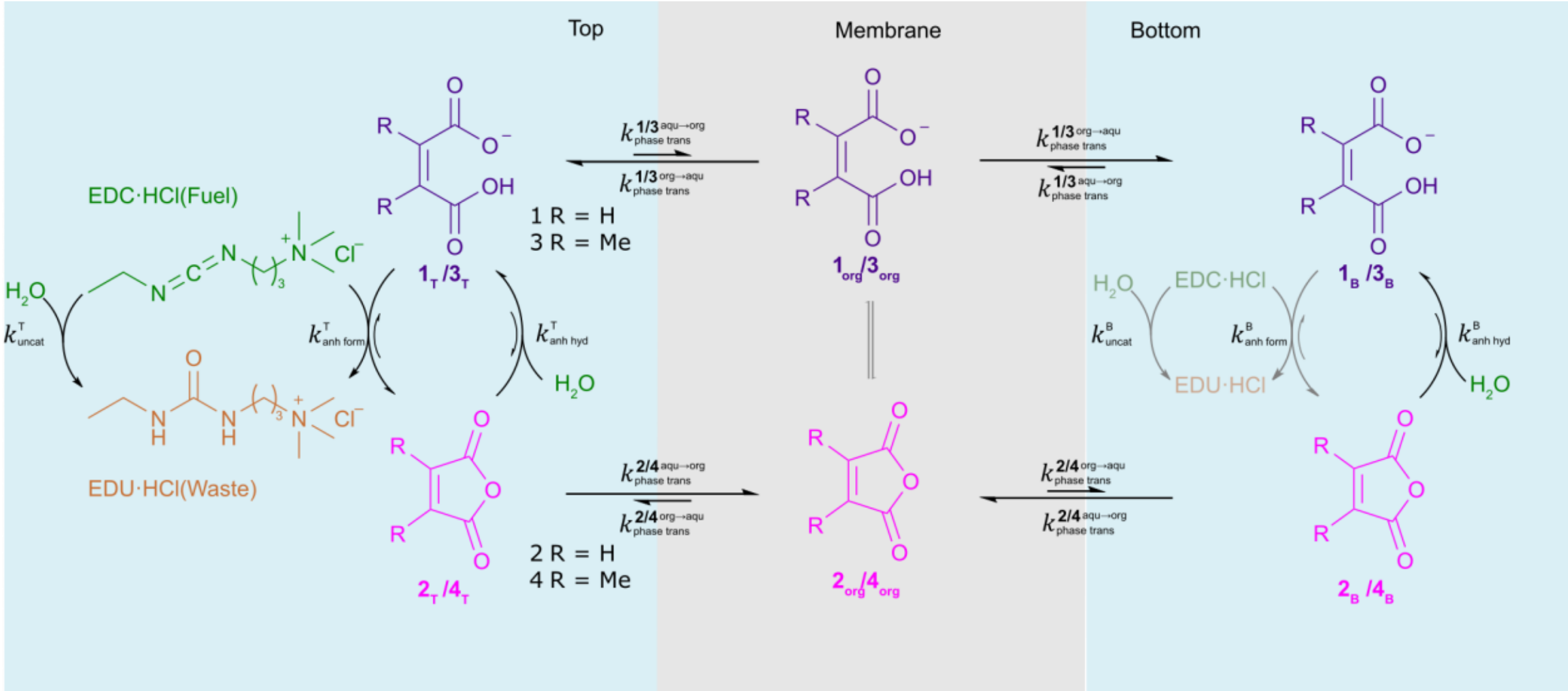


**Figure S3.** Schematic representation of the reaction network and transport behavior in the PAMPA setup.

### S2.2.1 Preparation of Buffers and Samples for Active Transport

Diacid **1** (17.4 mg, 0.15 mmol) was dissolved in MES buffer (3.0 mL, 250 mM, pH 6.0) to give a 50 mM stock solution. The addition of **1** did not change the $pH_{obs}$. To obtain a 50 mM stock solution of **3**, anhydride **4** (19.2 mg, 0.15 mmol) was dissolved in MES buffer (3.0 mL 250 mM, pH 6.0) and left to hydrolyze overnight. $^1$H NMR spectroscopy confirmed that hydrolysis was complete. These stock solutions were later diluted with additional MES buffer (250 mM, pH 6.0) to obtain 5 mM solutions of **1** or **3** for the PAMPA assay.

### S2.2.2 HPLC Calibration of Diacids 1 and 3

For analysis of **1** and **3**, isocratic methods were used with MeCN (+0.1% formic acid, *v*/*v*) and $H_2O$ (+0.1% formic acid, *v*/*v*) at ratios of 70:30 (*v*/*v*) for **1** and 30:70 (v/v) for **3**. The flow rate was 0.6 mL/min, and the run time was 6 min for **1** and 4 min for 3. UV detection was performed at 210 nm. The retention times were 2.9 min for **1** and 1.8 min for **3**.

1 mL samples of the diacids at different concentrations (0.0125–0.3 mM) were prepared in a MES buffer (5 mM, $pH_{obs}$ 6.0) analysed by HPLC to give the calibration curve. Each calibration was repeated in triplicate.

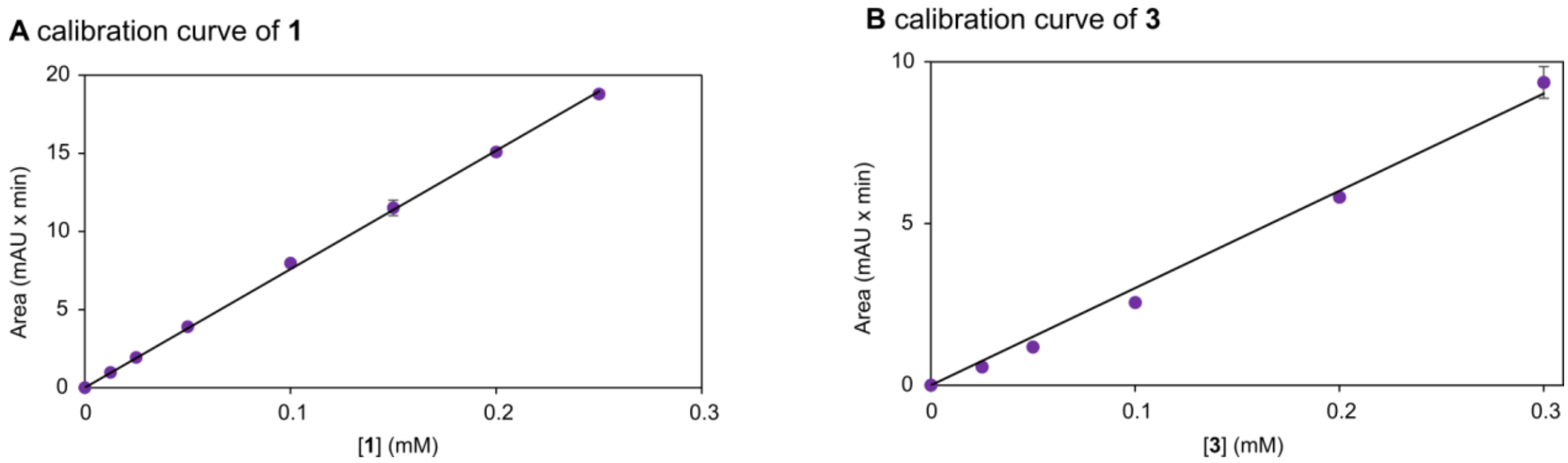


**Figure S4.** HPLC calibration curves for **A** diacid **1**, **B** diacid **3**, dissolved in MES buffer (5 mM, $pH_{obs}$ 6.0). Concentrations refer to the concentration of acid in the HPLC vial. The solid black line is a linear fit of the experimental data.

### S2.2.3 Negligible Passive Transport of Acids and Fuel

For passive transport experiments, the top compartment (5 mM of **1**, **3**, or 100 mM EDC·HCl fuel in 250 mM MES buffer, $pH_{obs}$ 6.0) and the bottom compartment (250 mM MES buffer, $pH_{obs}$ 6.0) were separated by a membrane of DOPC in dodecane (4%). The passive transport of the diacids was monitored by HPLC for 8 h, while for fuel, transport was monitored by NMR for 24 hr.

For HPLC analysis, 20 µL were sampled from both compartments. Top compartment samples were diluted to 1 mL with Milli-Q water, while samples from the bottom compartment were diluted to only 100 µL. To keep the overall volumes unchanged during PAMPA, MES buffer (20 µL, 250 mM MES, $pH_{obs}$ 6.0) was added back to both compartments after sampling. The calculation of the concentration considers this dilution.

To prepare the NMR samples, 100 µL of samples were taken from both compartments and diluted with 400 µL $D_2O$.

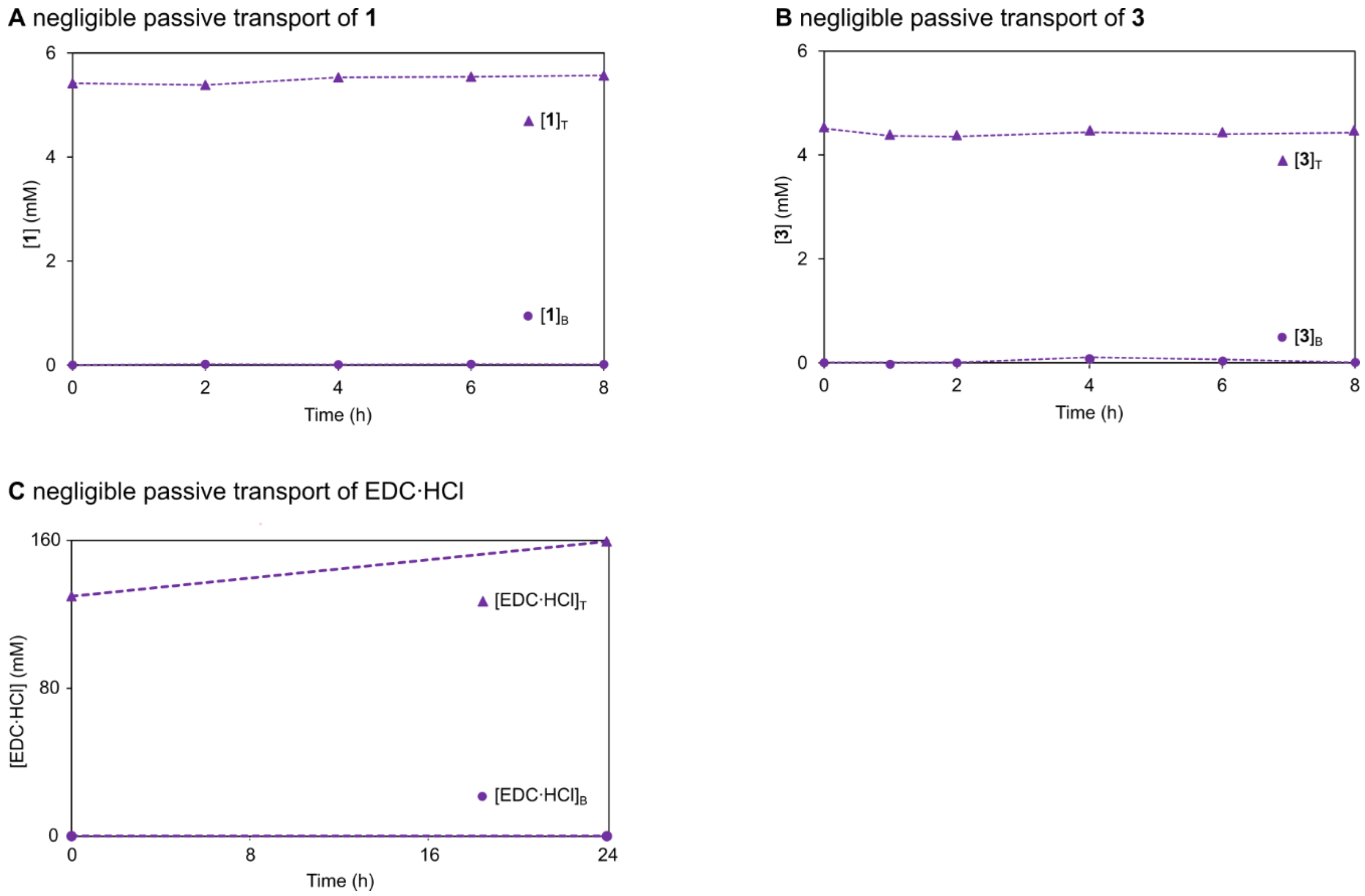


**Figure S5.** Concentration evolution of: **A** Diacid **1** starting from $[\mathbf{1}]_{T,0}$ = 5 mM, $[\mathbf{1}]_{B,0}$ = 0 mM, monitored by HPLC; **B** **3** starting from $[\mathbf{3}]_{T,0}$ = 5 mM, $[\mathbf{3}]_{B,0}$ = 0 mM, monitored by HPLC; **C** EDC·HCl starting from $[EDC{\cdot}HCl]_{T,0}$ = 100 mM, $[EDC{\cdot}HCl]_{B,0}$ = 0 mM, monitored by $^{1}H$ NMR spectroscopy. Top compartment marked with purple triangles and bottom with purple dots.

## S2.2.4 Active Transport Experiments

The majority of active transport experiments are detailed in the main text (Figure 2B), here experiments were performed with diacid **1** with an increasing concentration of fuel (Figure S6A and B). Additionally, to investigate whether lipid placement had any effect on transport an experiment was carried out in which the lipids coated the surface that is typically in contact with the bottom compartment (Figure S6C).

A typical active transport experiment was completed as follows: the top (200 µL) and bottom (300 µL) compartments consisted of MES buffer (250 mM, $pH_{obs}$ 6.0) containing equal concentrations of diacid **1** or **3** (ca. 5 mM). Final diacid concentration was calculated from HPLC calibration (Section S2.2.2, 3.74 mM for Figure 2B in the main text and 6 mM for Figure S6). Stock solutions of EDC·HCl (9.6 mg, 0.05 mmol, 1 M and 38.4 mg, 0.2 mmol, 4 M, respectively) were prepared in MES buffer (50 µL, 250 mM, $pH_{obs}$ 6.0). To initiate the assay, 20 µL of the fuel was added to the top compartment (final EDC·HCl concentration 100 mM or 400 mM). For bottom to top experiments (Figure S6C), 30 µL of the 4 M EDC·HCl stock solution was added to the bottom compartment instead. The preparation of samples for HPLC was done as previously described (Section S2.2.3).

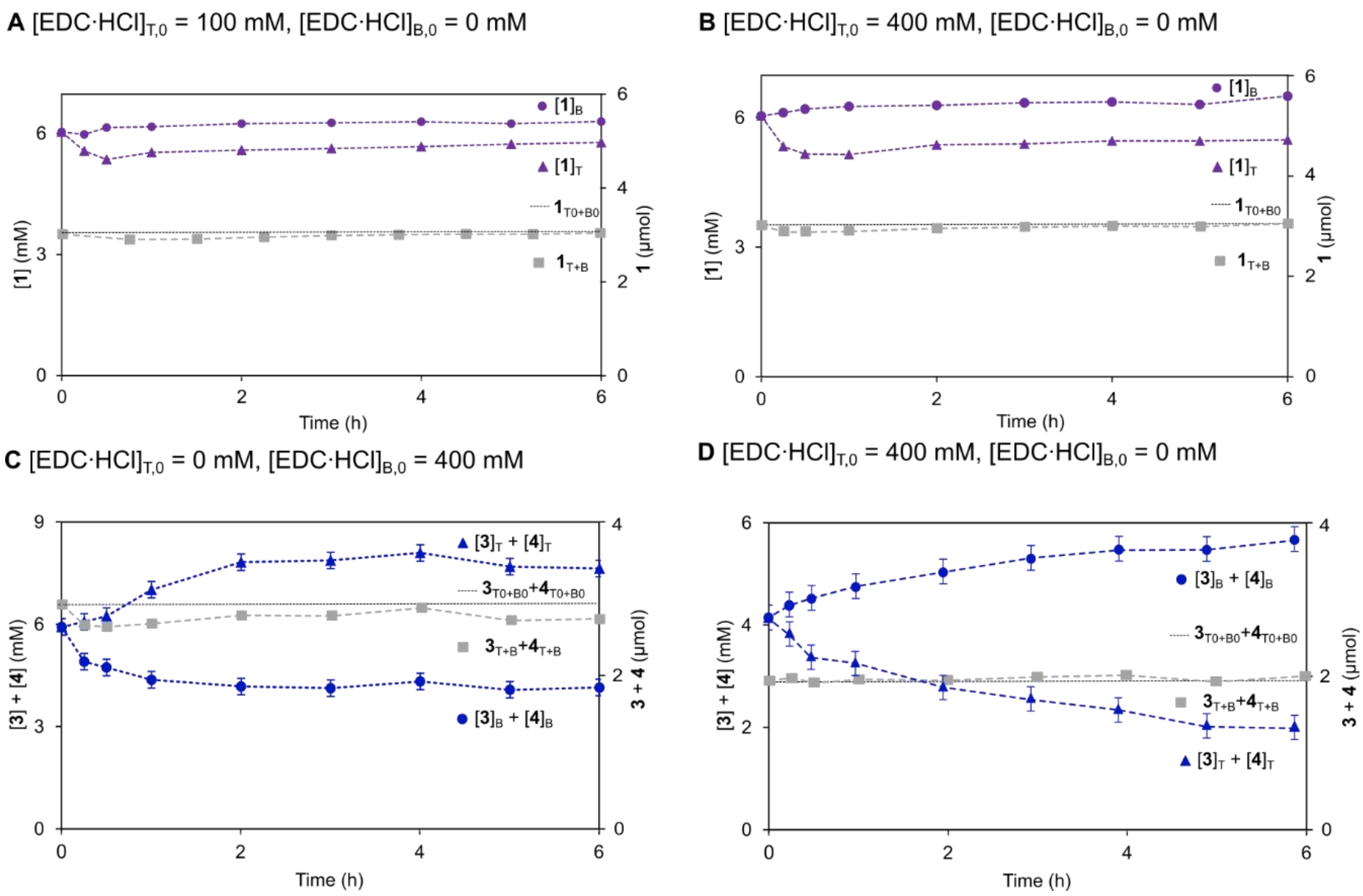


**Figure S6.** Evolution of [**1**] or [**3**] over time in compartment T (triangles) and B (circles) as observed via HPLC, together with the expected total moles of **1** or **3** + **4** in both compartments (full grey line) and the calculated total moles based on experimental data (grey squares). Transport experiments performed in MES buffer (250 mM, $pH_{obs}$ 6.0). **A** $[\mathbf{1}]_{T,0} = [\mathbf{1}]_{B,0}$ = 6 mM, $[EDC\cdot HCl]_{T,0}$ = 100 mM, $[EDC\cdot HCl]_{B,0}$ = 0 mM; **B** $[\mathbf{1}]_{T,0} = [\mathbf{1}]_{B,0}$ = 6 mM, $[EDC\cdot HCl]_{T,0}$ = 400 mM, $[EDC\cdot HCl]_{B,0}$ = 0 mM. **C** $[\mathbf{3}]_{T,0} = [\mathbf{3}]_{B,0}$ = 6 mM, $[EDC\cdot HCl]_{T,0}$ = 0 mM, $[EDC\cdot HCl]_{B,0}$ = 400 mM. **D** $[\mathbf{3}]_{T,0} = [\mathbf{3}]_{B,0}$ = 4 mM, $[EDC\cdot HCl]_{T,0}$ = 400 mM, $[EDC\cdot HCl]_{B,0}$ = 0 mM. The error bars were calculated based on two independent bottom-to-top transport experiments and the average from all the data points in both two aqueous compartments.

## S2.3. Isolated Reaction Kinetics

### S2.3.1 Transient Formation of Anhydrides monitored by $^1$H NMR Spectroscopy

A 250 mM deuterated MES buffer solution was prepared by dissolving MES (244 mg, 1.25 mmol) in $D_2O$ (5 mL). The $pD_{obs}$ was adjusted to 6.00 with NaOD.

A DSS stock solution was prepared by dissolving DSS (5.48 mg) in $D_2O$ (0.5 mL, final concentration = 50 mM).

An EDC·HCl solution was prepared by dissolving EDC·HCl (9.6 mg) in 250 mM MES buffer (20 µL, final [EDC·HCl] = 2.5 M). This solution was freshly prepared just before measurements to avoid the hydration of EDC·HCl in MES buffer.

Experiments were performed in $D_2O/H_2O$ (1:9, *v*/*v*) as follows: a dry NMR tube was charged with DSS (10 µL of 50 mM stock solution, final [DSS] = 1 mM), **1** or **3** (50 µL of 50 mM stock solution, final [**1**] or [**3**] = 5 mM), deuterated MES buffer (40 µL) and non-deuterated MES buffer (380 µL). A $^1$H NMR spectrum was taken, EDC·HCl fuel (20 µL of 2.5 M stock solution in MES buffer, final [EDC·HCl] = 100 mM) was added to initiate the experiment, and $^1$H NMR spectra were recorded every 5 min.

The reaction was modeled according to irreversible first order kinetics. Each experiment was repeated in triplicate to obtain an average rate of anhydride formation and hydrolysis.

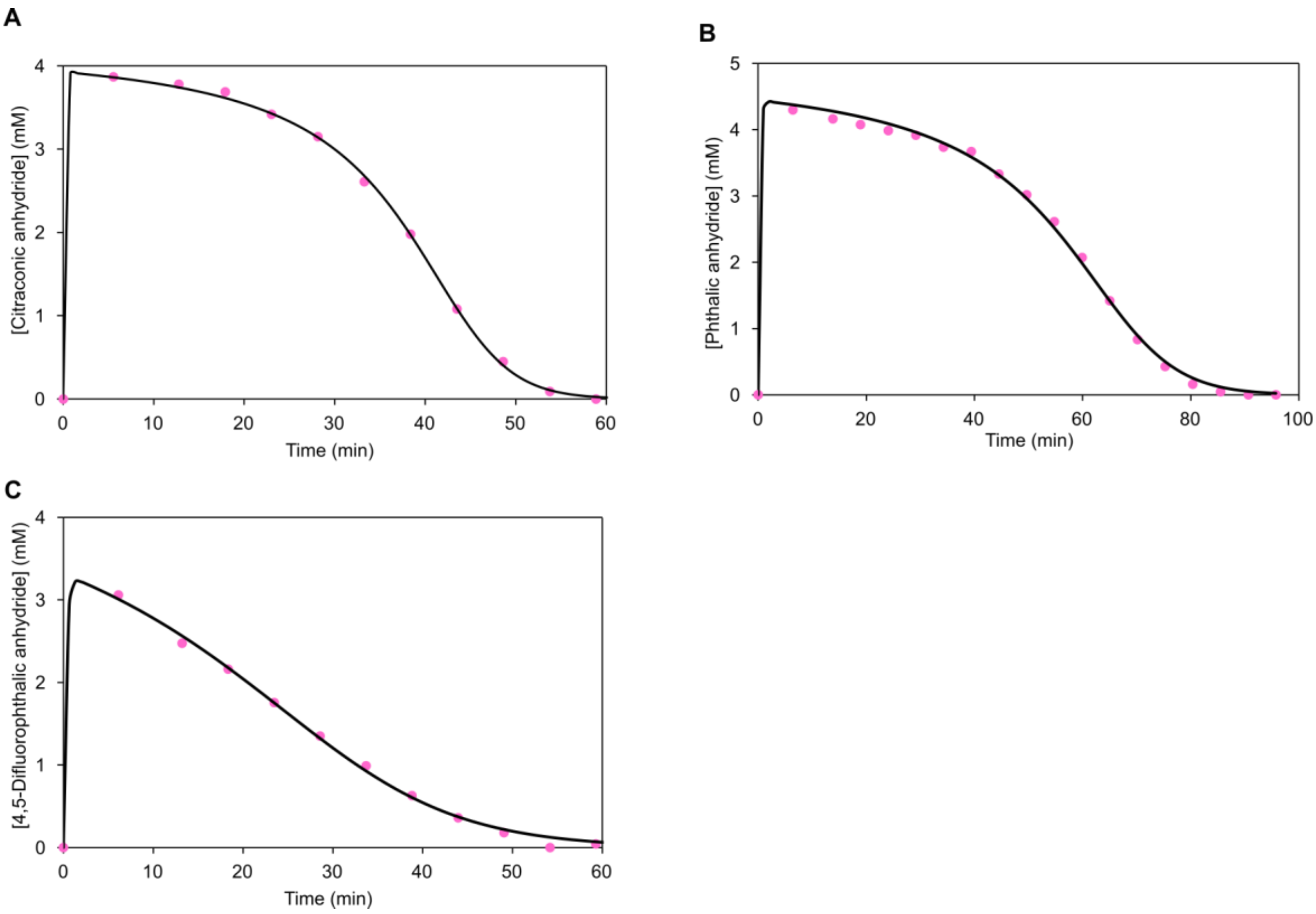


**Figure S7.** NMR kinetic trace (light purple dots) and fit (black line) for the transient formation of: **A** citraconic anhydride **B** phthalic anhydride, and **C** 4,5-difluorophthalic anhydride, and their subsequent hydrolysis at $p(H,D)_{obs}$ 6.0. Conditions: [acid] = 5 mM, [EDC·HCl] = 100 mM, [MES] = 250 mM in $D_2O/H_2O$ (1:9, *v*/*v*), 298 K, 400 MHz. Data fitting affords for **A** a second-order anhydride formation rate constant of $6.87 \times 10^{-1}$ $M^{-1}$ $s^{-1}$ and a first-order anhydride hydrolysis rate constant of $1.13 \times 10^{-2}$ $s^{-1}$ for citraconic anhydride; for **B** a second-order anhydride formation rate constant of $5.66 \times 10^{-1}$ $M^{-1}$ $s^{-1}$ and a first-order anhydride hydrolysis rate constant of $6.87 \times 10^{-3}$ $s^{-1}$ for phthalic anhydride; for **C** a second-order anhydride formation rate constant of $3.88 \times 10^{-1}$ $M^{-1}$ $s^{-1}$ and a first-order anhydride hydrolysis rate constant of $1.98 \times 10^{-2}$ $s^{-1}$ for 4,5-difluorophthalic anhydride.

### S2.3.2 Kinetic Modeling of Transient Anhydrides Formation

A Python code was used to fit the $^1H$ NMR spectroscopy data to the ordinary differential equations describing formation, hydrolysis of **4**. In particular, the method *differential evolution* of Python Scipy was employed to minimize the mean squared error between experimental and simulated data. The function *odeint* was used to integrate the differential equations numerically.

The formation of **4** and/or its hydrolysis were modeled thanks to the differential equations:

$$\frac{d[\mathbf{3}]}{dt} = -k_{anh\ form}[\mathrm{EDC{\cdot}HCl}][\mathbf{3}] + k_{anh\ hyd}[\mathbf{4}] \qquad \text{(Equation S3)}$$

$$\frac{d[\mathrm{EDC{\cdot}HCl}]}{dt} = -k_{anh\ form}[\mathrm{EDC{\cdot}HCl}][\mathbf{3}] \qquad \text{(Equation S4)}$$

Where the $k_{anh\ hyd}$ is the hydrolysis rate of anhydride and $k_{anh\ form}$ is the reaction rate of anhydride formation. We note that $k_{anh\ hyd}$ is treated as a pseudo-first order rate due to the vast excess of water, and thus the rate constant implicitly includes the (effectively constant) concentration of water, and the mass conservation laws:

$$[\mathbf{3}]_0 = [\mathbf{3}] + [\mathbf{4}] \quad \text{(Equation S5)}$$

$$[\mathrm{EDC{\cdot}HCl}]_0 = [\mathrm{EDC{\cdot}HCl}] + [\mathrm{EDU{\cdot}HCl}] \quad \text{(Equation S6)}$$

Where $_0$ indicates initial concentration of the associated species. Under the experimental conditions observed, both reactions described by $k_{\mathrm{anh\ form}}$ and $k_{\mathrm{anh\ hyd}}$ are practically irreversible, including their microscopic reverse process requires introducing additional assumptions and has no significant impact on the fitting results.

The initial concentrations (fixed) were set based on experimental values. When performing data fitting of NMR experiments on transient **4** formation and subsequent hydrolysis, $k_{\mathrm{anh\ hyd}}$ was set at the values experimentally determined in previous experiments for **1**, and allowed to vary within 200% of their value. The mean squared error was calculated from concentrations extracted from $^1$H NMR data when considering transient formation of **4**.

### S2.3.3 Competition Between Hydrolysis and Phase Transfer of Anhydrides 2 & 4

In the PAMPA setup, 340 µL and 200 µL of 250 mM MES buffer at pH$_{\mathrm{obs}}$ 6.0 were added respectively to bottom and top compartments as aqueous phases, separated by 5 µL of 4% DOPC solution in dodecane. For the experiments involving anhydride **2**, 1.7 mg (0.017 mmol) of **2** were dissolved in MeCN (70 µL, affording [**2**] = 240 mM) and 10 µL of this solution was added to compartment T while MeCN (10 µL, without **2**) was added to compartment B. Six replicates are prepared and started at same time. Time zero samples were taken from both aqueous phases before the addition of **2** while the other samples were taken after incubation for 2, 5, 10, 15, 30, 60 minutes from different replicates. The procedure used in experiment on **4** is the same, only replacing **2** with **4**.

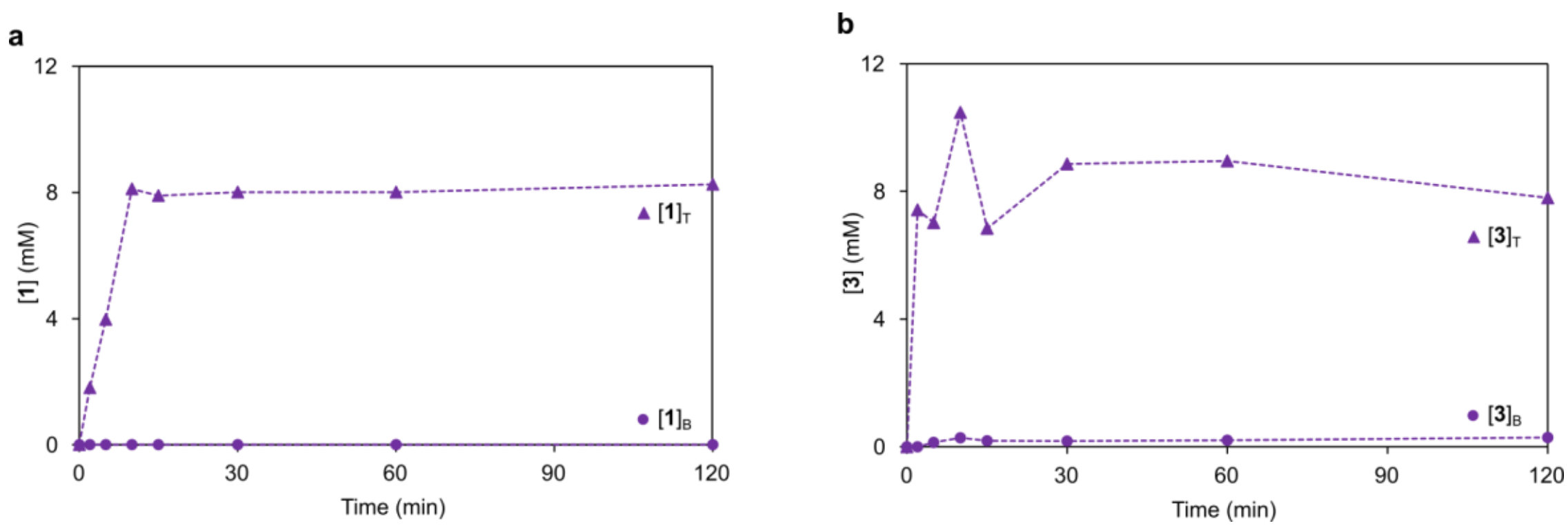


**Figure S8.** Evolution in time of **A** [**1**]$_T$ (purple triangles) and [**1**]$_B$ (purple dots), observed upon adding **2** to the aqueous compartment T. Conditions for both aqueous phases: [MES] = 250 mM, pH$_{\mathrm{obs}}$ 6.0. **B** [**3**]$_T$ (purple triangles) and [**3**]$_B$ (purple dots), same procedure with **4**. The high value for [**3**]$_T$ observed at $t$ = 10 min is due to an operational error associated with the sampling procedure. That data point was not used to calculate the concentration difference between the two aqueous phases.

### S2.3.4 Phase Transfer and Hydrolysis of 4

The hydrophobic phase was prepared by dissolving **4** (0.8 mg) in 20 µL of DOPC (4%) in dodecane (the anhydride only partially dissolved). The top plate was turned upside down and the lipids (5 µL) were coated onto the PVDF support that is typically in contact with the bottom compartment. The top plate was then turned back to its original orientation, and 250 mM MES buffer (pH$_{\mathrm{obs}}$ 6.0) was added to top (200 µL) and bottom (300 µL) compartments. 20 µL samples were taken from the top compartment every 30 minutes and diluted to 1 mL with Milli-Q water. For this fitting the PAMPA set-up was considered a biphasic system. The phase

transfer rate of **3** was negligible, and data from the previous study was applied.[S3] While the phase transfer rate of **4** from aqueous to organic phase ($k_{\text{phase trans}}^{\mathbf{4}\text{aqu}\rightarrow\text{org}}$) was also unknown, changing this value led to a similar fitting result. This is attributed to the fact that the hydrolysis rate is a magnitude faster than this process, leading to predominantly acid in the aqueous phase, rendering this parameter largely inconsequential.

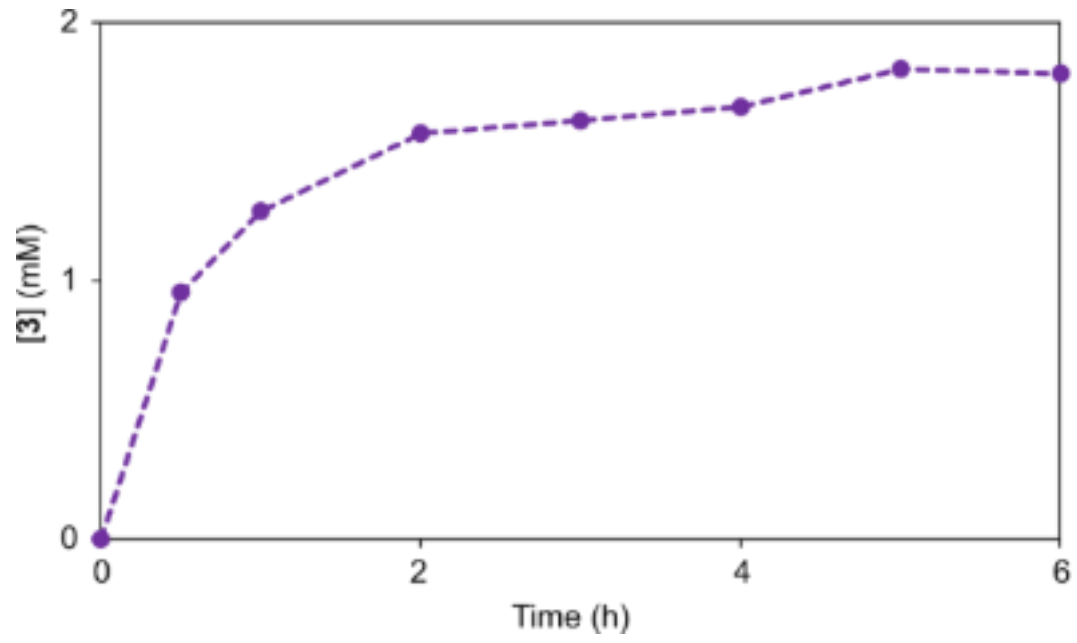


**Figure S9.** Evolution in time of the $[\mathbf{3}]_T$ (purple triangles) observed upon adding **4** to the hydrophobic phase. Data fitting affords the phase transfer rate of **4** ($k_{\text{phase trans}}^{\mathbf{4}\text{org}\rightarrow\text{aqu}}$) from organic to top aqueous phase as 0.00205 $s^{-1}$.

### S2.3.5 Kinetic Modeling of Phase Transfer Experiments (PAMPA)

If the volumes of the two phases were all equal, we could simply write the standard kinetic equations associated to the scheme in Figure S3 to describe the evolution of species concentration over time. However, since the volume of the organic phase ($V_{org}$ = 5 μL) and the two aqueous compartments also have different volume ($V_T$ = 200 μL, $V_B$ = 300 μL), the model must account for the fact that, when n moles of a species, say of **4**, migrate from the aqueous to the organic phase in a unit of time, the corresponding instantaneous concentration drops in one phase (e.g., $d_t\,[\mathbf{4}]_{aqu} = -k_{\text{phase trans}}^{\mathbf{4}\text{aqu}\rightarrow\text{org}}[\mathbf{4}]_{aqu}$, where $d_t$ indicates the derivative with respect to time of the following argument, here $[\mathbf{4}]_{aqu}$) is not equal to the concentration increment in the other (e.g., $d_t\,[\mathbf{4}]_{org} \neq k_{\text{phase trans}}^{\mathbf{4}\text{aqu}\rightarrow\text{org}}[\mathbf{4}]_{aqu}$). Indeed, to respect mass conservation, we must have that the number of moles leaving one phase (e.g., $d_t\, n\mathbf{4}_{aqu} = -k_{\text{phase trans}}^{\mathbf{4}\text{aqu}\rightarrow\text{org}}[\mathbf{4}]_{aqu} V_{aqu}$) equals the number of moles entering the other (e.g., $d_t\, n\mathbf{4}_{org} = k_{\text{phase trans}}^{\mathbf{4}\text{aqu}\rightarrow\text{org}}[\mathbf{4}]_{aqu} V_{aqu} = -d_t\, n\mathbf{4}_{aqu}$). Therefore, the kinetic equations describing the mass transfer of **4** in a biphasic system of aqueous and organic phase read:

$$\begin{aligned} d_t n\mathbf{4}_{aqu} &= -k_{\text{phase trans}}^{\mathbf{4}\text{aqu}\rightarrow\text{org}}[\mathbf{4}]_{aqu}V_{aqu} + k_{\text{phase trans}}^{\mathbf{4}\text{org}\rightarrow\text{aqu}}[\mathbf{4}]_{org}V_{org} \\ &= -\tilde{k}_{\text{phase trans}}^{\mathbf{4}\text{aqu}\rightarrow\text{org}}[\mathbf{4}]_{aqu} + \tilde{k}_{\text{phase trans}}^{\mathbf{4}\text{org}\rightarrow\text{aqu}}[\mathbf{4}]_{org} \end{aligned} \quad \text{(Equation S7)}$$

$$\begin{aligned} d_t n\mathbf{4}_{org} &= k_{\text{phase trans}}^{\mathbf{4}\text{aqu}\rightarrow\text{org}}[\mathbf{4}]_{aqu}V_{aqu} - k_{\text{phase trans}}^{\mathbf{4}\text{org}\rightarrow\text{aqu}}[\mathbf{4}]_{org}V_{org} \\ &= \tilde{k}_{\text{phase trans}}^{\mathbf{4}\text{aqu}\rightarrow\text{org}}[\mathbf{4}]_{aqu} - \tilde{k}_{\text{phase trans}}^{\mathbf{4}\text{org}\rightarrow\text{aqu}}[\mathbf{4}]_{org} \end{aligned} \quad \text{(Equation S8)}$$

where we defined the modified rate constants $\tilde{k}$ (in units of L $s^{-1}$) embedding the volume factors. By dividing $d_t\, n\mathbf{4}_{aqu}$ and $d_t\, n\mathbf{4}_{org}$ by $V_{aqu}$ and $V_{org}$, respectively, we recover the correct form of the kinetic equations describing the evolution of [**4**] in time due to phase transfer only (the same procedure leads to the correct expressions for [**3**]):

$$d_t[\mathbf{4}]_{aqu} = \frac{-\tilde{k}_{\text{phase trans}}^{\mathbf{4}\text{aqu}\rightarrow\text{org}}[\mathbf{4}]_{aqu}}{V_{aqu}} + \frac{\tilde{k}_{\text{phase trans}}^{\mathbf{4}\text{org}\rightarrow\text{aqu}}[\mathbf{4}]_{org}}{V_{aqu}} \quad \text{(Equation S9)}$$

$$d_t[\mathbf{4}]_{org} = \frac{\tilde{k}_{\text{phase trans}}^{\mathbf{4}\text{aqu}\rightarrow\text{org}}[\mathbf{4}]_{aqu}}{V_{org}} - \frac{\tilde{k}_{\text{phase trans}}^{\mathbf{4}\text{org}\rightarrow\text{aqu}}[\mathbf{4}]_{org}}{V_{org}} \quad \text{(Equation S10)}$$

The full kinetic model for phase transfer experiment of [**4**] in a biphasic system therefore reads:

$$d_t[\mathbf{3}]_{aqu} = \frac{-\tilde{k}_{\text{phase trans}}^{\mathbf{3}\text{aqu}\rightarrow\text{org}}[\mathbf{3}]_{aqu}}{V_{aqu}} + \frac{\tilde{k}_{\text{phase trans}}^{\mathbf{3}\text{org}\rightarrow\text{aqu}}[\mathbf{3}]_{org}}{V_{aqu}} + k_{anh\ hyd}[\mathbf{4}]_{aqu} \quad \text{(Equation S11)}$$

$$d_t[\mathbf{3}]_{org} = \frac{\tilde{k}_{\text{phase trans}}^{\mathbf{3}\text{aqu}\rightarrow\text{org}}[\mathbf{3}]_{aqu}}{V_{org}} - \frac{\tilde{k}_{\text{phase trans}}^{\mathbf{3}\text{org}\rightarrow\text{aqu}}[\mathbf{3}]_{org}}{V_{org}} \quad \text{(Equation S12)}$$

$$d_t[\mathbf{4}]_{aqu} = \frac{-\tilde{k}_{\text{phase trans}}^{\mathbf{4}\text{aqu}\rightarrow\text{org}}[\mathbf{4}]_{aqu}}{V_{aqu}} + \frac{\tilde{k}_{\text{phase trans}}^{\mathbf{4}\text{org}\rightarrow\text{aqu}}[\mathbf{4}]_{org}}{V_{aqu}} - k_{anh\ hyd}[\mathbf{4}]_{aqu} \quad \text{(Equation S13)}$$

$$d_t[\mathbf{4}]_{org} = \frac{\tilde{k}_{\text{phase trans}}^{\mathbf{4}\text{aqu}\rightarrow\text{org}}[\mathbf{4}]_{aqu}}{V_{org}} - \frac{\tilde{k}_{\text{phase trans}}^{\mathbf{4}\text{org}\rightarrow\text{aqu}}[\mathbf{4}]_{org}}{V_{org}} \quad \text{(Equation S14)}$$

## S2.4. Full Kinetic Modeling of Multi-Phase Experiments

We now introduce the kinetic model that was used to fit the active transport of **3** at $pH_{obs}$ = 6.0 upon localized addition of EDC·HCl in **T**, monitored by HPLC, which is presented in Figure S3. As for the single-phase and two-phase kinetic modeling, the method *differential evolution* of Python Scipy was employed to minimize the mean squared error between experimental and simulated data. Considering the comparatively slow hydrolysis of **4**, the concentration recorded with HPLC should be a sum of both [**3**] and [**4**], which is used to calculate the errors with the *differential evolution*. And since the two aqueous phases have different volumes, the errors were calculated based on moles in the two compartments instead of concentrations. The function *odeint* was used to integrate the differential equations numerically.

The model comprises all the species and reactions depicted in Figure S3 of the main text, as well as the background EDC·HCl hydration ($k_{\text{uncat}}$) which is determined in previous study.[S3] As detailed in the main text and in the previous sections, we conducted independent experiments to estimate the value of most rate constants.

The EDC·HCl formation from EDU·HCl and the microscopic reverse of **4** hydrolysis (i.e., spontaneous anhydride formation in buffered water) were established to be negligible and set to zero in the kinetic model.

The kinetic rate constants associated to the EDC·HCl-promoted conversion of **3** into **4** ($k^{\text{T/B}}_{\text{anh form}}$ = 0.45 $M^{-1}$ $s^{-1}$), hydrolysis of **4** ($k^{\text{T/B}}_{\text{anh hyd}}$ = 5.52 × $10^{-3}$ $s^{-1}$), EDC·HCl background hydration ($k^{\text{T/B}}_{\text{uncat}}$ = 7.78 × $10^{-6}$ $s^{-1}$), and phase transfer rate of **4** from organic to aqueous phase ($k^{\mathbf{4}\text{org}\rightarrow\text{aquT/B}}_{\text{phase trans}}$ = 2.05 × $10^{-3}$ $s^{-1}$) have been determined experimentally. As a result, the rate constants associated to migration of **4** from the aqueous to the organic phase ($k^{\mathbf{4}\text{aquT/B}\rightarrow\text{org}}_{\text{phase trans}}$) is the only parameter which needs to be fitted.

Considering the volume factor, the phase transfer rate $\tilde{k}^{\mathbf{4}\text{aquT/B}\rightarrow\text{org}}_{\text{phase trans}} = k^{\mathbf{4}\text{aquT}\rightarrow\text{org}}_{\text{phase trans}} V_T = k^{\mathbf{4}\text{aquB}\rightarrow\text{org}}_{\text{phase trans}} V_B$ and the full kinetic model therefore reads:

$$d_t[\mathbf{3}]_T = \frac{-\tilde{k}^{\mathbf{3}\text{aquT}\rightarrow\text{org}}_{\text{phase trans}}[\mathbf{3}]_T + \tilde{k}^{\mathbf{3}\text{org}\rightarrow\text{aquT}}_{\text{phase trans}}[\mathbf{3}]_{org}}{V_T} - k^{\text{Top}}_{\text{anh form}}[\text{EDC·HCl}]_T[\mathbf{3}]_T + k^{\text{Top}}_{\text{anh hyd}}[\mathbf{4}]_T \quad \text{(Equation S15)}$$

$$d_t[\mathbf{3}]_{org} = \frac{\tilde{k}^{\mathbf{3}\text{aquT}\rightarrow\text{org}}_{\text{phase trans}}[\mathbf{3}]_T}{V_{org}} + \frac{\tilde{k}^{\mathbf{3}\text{aquB}\rightarrow\text{org}}_{\text{phase trans}}[\mathbf{3}]_B}{V_{org}} - \frac{\tilde{k}^{\mathbf{3}\text{org}\rightarrow\text{aquT}}_{\text{phase trans}}[\mathbf{3}]_{org}}{V_{org}} - \frac{\tilde{k}^{\mathbf{3}\text{org}\rightarrow\text{aquB}}_{\text{phase trans}}[\mathbf{3}]_{org}}{V_{org}} \quad \text{(Equation S16)}$$

$$d_t[\mathbf{3}]_B = \frac{-\tilde{k}^{\mathbf{3}\text{aquB}\rightarrow\text{org}}_{\text{phase trans}}[\mathbf{3}]_B + \tilde{k}^{\mathbf{3}\text{org}\rightarrow\text{aquB}}_{\text{phase trans}}[\mathbf{3}]_{org}}{V_B} - k^{\text{Bottom}}_{\text{anh form}}[\text{EDC·HCl}]_B[\mathbf{3}]_B + k^{\text{Bottom}}_{\text{anh hyd}}[\mathbf{4}]_B \quad \text{(Equation S17)}$$

$$d_t[\mathbf{4}]_T = \frac{-\tilde{k}^{\mathbf{4}\text{aquT}\rightarrow\text{org}}_{\text{phase trans}}[\mathbf{4}]_T + \tilde{k}^{\mathbf{4}\text{org}\rightarrow\text{aquT}}_{\text{phase trans}}[\mathbf{4}]_{org}}{V_T} + k^{\text{Top}}_{\text{anh form}}[\text{EDC·HCl}]_T[\mathbf{3}]_T - k^{\text{Top}}_{\text{anh hyd}}[\mathbf{4}]_T \quad \text{(Equation S18)}$$

$$d_t[\mathbf{4}]_{org} = \frac{\tilde{k}^{\mathbf{4}\text{aquT}\rightarrow\text{org}}_{\text{phase trans}}[\mathbf{4}]_T}{V_{org}} + \frac{\tilde{k}^{\mathbf{4}\text{aquB}\rightarrow\text{org}}_{\text{phase trans}}[\mathbf{4}]_B}{V_{org}} - \frac{\tilde{k}^{\mathbf{4}\text{org}\rightarrow\text{aquT}}_{\text{phase trans}}[\mathbf{4}]_{org}}{V_{org}} - \frac{\tilde{k}^{\mathbf{4}\text{org}\rightarrow\text{aquB}}_{\text{phase trans}}[\mathbf{4}]_{org}}{V_{org}} \quad \text{(Equation S19)}$$

$$d_t[\mathbf{4}]_B = \frac{-\tilde{k}^{\mathbf{4}\text{aquB}\to\text{org}}_{\text{phase trans}}[\mathbf{3}]_B + \tilde{k}^{\mathbf{4}\text{org}\to\text{aquB}}_{\text{phase trans}}[\mathbf{3}]_{org}}{V_B} + k^{\text{Bottom}}_{\text{anh form}}[\text{EDC·HCl}]_B[\mathbf{3}]_B - k^{\text{Bottom}}_{\text{anh hyd}}[\mathbf{4}]_B$$

(Equation S20)

$$d_t[\boldsymbol{EDC}]_T = -k^{\text{Top}}_{\text{anh form}}[\text{EDC·HCl}]_T[\mathbf{3}]_T - k^{\text{Top}}_{\text{uncat}}[\text{EDC·HCl}]_T$$ (Equation S21)

$$d_t[\boldsymbol{EDC}]_B = -k^{\text{Bottom}}_{\text{anh form}}[\text{EDC·HCl}]_B[\mathbf{3}]_B - k^{\text{Bottom}}_{\text{uncat}}[\text{EDC·HCl}]_B$$ (Equation S22)

Since the slow diffusion will lead to less anhydride reaching membrane, we introduce a factor ($k^{\mathbf{4}\text{aquT/B}\to\text{org}}_{\text{phase trans}}$ = $k^{\mathbf{4}\text{aquT}\to\text{org}}_{\text{phase trans\_}original}$ × factor) to account for the fact that only the molecules near the membrane will be able to partition into the organic phase. Therefore, in practice we optimize $k_{4aoT/B}$ in the model, starting from $k^{\mathbf{4}\text{aquT}\to\text{org}}_{\text{phase trans_}}$0 = $k^{\mathbf{4}\text{aquT}\to\text{org}}_{\text{phase trans_}original_}$0 × factor (factor = 0.006 as calculated by diffusion of a species with diffusion coefficient of $10^{-5}$ s $cm^{-2}$). The fitting starts with a $k^{\mathbf{4}\text{aquT}\to\text{org}}_{\text{phase trans_}original_}$0 which was calculated roughly with $k^{\mathbf{4}\text{org}\to\text{aquT/B}}_{\text{phase trans}}$ and partition coefficient of **2** between aqueous phase and organic phases.[S3] The fitting end with $\tilde{k}^{\mathbf{4}\text{aquT/B}\to\text{org}}_{\text{phase trans}}$ = 1.70 × $10^{-8}$ L $s^{-1}$ (giving $k^{\mathbf{4}\text{aquT}\to\text{org}}_{\text{phase trans}}$ = 8.52 × $10^{-5}$ $s^{-1}$ and $k^{\mathbf{4}\text{aquB}\to\text{org}}_{\text{phase trans}}$ = 5.68 × $10^{-5}$ $s^{-1}$).

The quality of the fitting was evaluated by applying the normalised root-mean-square error (NRMSE), which indicates a 5% error on how much the fit differs from the experimental data.

**Table S1.** Parameters for the kinetic model.

| Species | Top Compartment | Membrane | Bottom Compartment |
|---|---|---|---|
| Volume | 2.0 × $10^{-4}$ L | 5.0 × $10^{-6}$ L | 3.0 × $10^{-4}$ L |
| [**3**] | 4.12 mM | 0 mM | 4.12 mM |
| [**4**] | 0 mM | 0 mM | 0 mM |
| [EDC·HCl] | 400 mM | 0 mM | 0 mM |
| [EDU·HCl] | 0 mM | 0 mM | 0 mM |

**Table S2.** Individually determined rate constants for kinetic model. Most rates constants were obtained from isolated experiments (Section S2.3). For the full fit, rate constants for phase transfer of **4** from aqueous phases to organic phase was re-fitted to account for the actual transport behavior during the experiments.

| Constant | Value from independent experiments | Value from updated fit |
|---|---|---|
| $k_{uncat}^{T/B}$ [a] | $7.78 \times 10^{-6}$ $s^{-1}$ | Identical (fixed) |
| $k_{anh\,form}^{T/B}$ | $4.5 \times 10^{-1}$ $M^{-1}s^{-1}$ | Identical (fixed) |
| $k_{anh\,hyd}^{T/B}$ | $5.52 \times 10^{-3}$ $s^{-1}$ | Identical (fixed) |
| $k_{phase\,trans}^{\mathbf{3}org\to aqu}$ [a] | $2.08 \times 10^{-9}$ $s^{-1}$ | Identical (fixed) |
| $\tilde{k}_{\mathrm{phase\,trans}}^{\mathbf{3}\mathrm{org}\to\mathrm{aqu}}$ | $1.04 \times 10^{-14}$ $L^{-1}$ $s^{-1}$ | Identical (fixed) |
| $k_{phase\,trans}^{\mathbf{3}aqu\to org}$ [a] | $8.42 \times 10^{-25}$ $s^{-1}$ | Identical (fixed) |
| $\tilde{k}_{\mathrm{phase\,trans}}^{\mathbf{3}\mathrm{aqu}\to\mathrm{org}}$ | $4.21 \times 10^{-30}$ $L^{-1}$ $s^{-1}$ | Identical (fixed) |
| $k_{phase\,trans}^{\mathbf{4}org\to aqu}$ | $2.05 \times 10^{-3}$ $s^{-1}$ | Identical (fixed) |
| $\tilde{k}_{\mathrm{phase\,trans}}^{\mathbf{4}\mathrm{org}\to\mathrm{aqu}}$ | $1.02 \times 10^{-8}$ $L^{-1}$ $s^{-1}$ | Identical (fixed) |
| $k_{phase\,trans}^{\mathbf{4}aquT\to org}$ _original_0 | $2.87 \times 10^{-2}$ $s^{-1}$ | \ |
| $k_{phase\,trans}^{\mathbf{4}aquT\to org}$ | $1.72 \times 10^{-4}$ $s^{-1}$ | $8.52 \times 10^{-5}$ $s^{-1}$ |
| $k_{phase\,trans}^{\mathbf{4}aquB\to org}$ | | $5.68 \times 10^{-5}$ $s^{-1}$ |
| $\tilde{k}_{\mathrm{phase\,trans}}^{\mathbf{4}\mathrm{aquT/B}\to\mathrm{org}}$ | $3.44 \times 10^{-8}$ $L^{-1}$ $s^{-1}$ | $1.70 \times 10^{-8}$ $L^{-1}$ $s^{-1}$ |

[a] data from previous study [S3].

## S2.5. Thermodynamic Analysis of Active Transport with PAMPA

The free energy stored in the transport of **3** is defined as the free energy that would be released if the non-equilibrium distribution of **3** relaxed towards equilibrium by passive transport during the PAMPA experiment. Therefore, the stored free energy can be quantified as the difference between the Gibbs free energy density of a certain nonequilibrium concentration distribution after transport has occurred ($[\mathbf{3}]_T$, $[\mathbf{3}]_B$), and the Gibbs free energy density of the reference equilibrium distribution (e.g., $[\mathbf{3}]_{T,eq} = [\mathbf{3}]_{B,eq} = (2\times[\mathbf{3}]_T + 3\times [\mathbf{3}]_B)/5$ for pumping between aqueous compartments at the same $pH_{obs}$). Note that the free energy stored can be computed at any time, provided that the $[\mathbf{3}]_T$ and $[\mathbf{3}]_B$ are known. According to our definition, **3** present in the organic phase does not contribute to the free energy stored.

In the ideal approximation, the free energy stored in the system takes the following form:[S4]

$$G_{stored} = \frac{RT\left(V_T\left([\mathbf{3}]_T \cdot ln\frac{[\mathbf{3}]_T}{[\mathbf{3}]_{T,eq}} + [\mathbf{3}]_{T,eq} - [\mathbf{3}]_T\right) + V_B\left([\mathbf{3}]_B \cdot ln\frac{[\mathbf{3}]_B}{[\mathbf{3}]_{B,eq}} + [\mathbf{3}]_{B,eq} - [\mathbf{3}]_B\right)\right)}{(V_T + V_B)} \quad \text{(Equation S23)}$$

where $R$ and $T$ are the gas constant and the temperature in Kelvin, respectively. Equation S23 was used to compute the free energy storage values reported in the main text at 298.15 K, unless specified otherwise.

# S3. $^1$H NMR Spectroscopy Studies

## S3.1. Solvent Suppression Protocol for $^1$H NMR Studies of LUVs

The $H_2O$ solvent signal was attenuated using the Robust-5 pulse sequence due to its high experimental tolerance.[S5]

$^1$H NMR spectra were collected using a Varian/Agilent 500 MHz spectrometer equipped with a broadband observe (BBO) probe with a maximum pulsed field gradient of 64 G cm$^{-1}$. 16 transients, each comprising 32,768 complex data points and a spectral width of 10 kHz, were collected for each spectrum. Two steady state transients were used. Each acquisition required 7.5 min. Secondary notches produced by the Robust-5 pulse sequence were set 6.7 ppm either side of the water frequency. 0.5 ms long rectangular gradient pulses with an amplitude of 28.3 G cm$^{-1}$ (first pair of gradient pulses) and 4.9 G cm$^{-1}$ (second pair) were used. The gradient stabilization delay was 0.5 ms. The first pair of lock pre-focusing field gradients were separated by 1.5 ms from the first radio-frequency pulse. The repetition time was 26.8 s, of which 3.3 s comprised the acquisition time. This repetition time was calculated setting it to five time the longest $T_1$ of the sample (to ensure the signals were not attenuated due to fast pulsing, Section S3.3). $T_1$ constants were calculated in $H_2O$:$D_2O$ (9:1 *v*/*v*) mixture with an in-house modified Robust-5 pulse sequence with an added inversion recovery module.

## S3.2. Experimental Procedures

### S3.2.1 General Preparation of Buffer Solutions

Deuterated MES buffer solutions (100 mM) were prepared by dissolving MES (488 mg), NaCl (292 mg, 200 mM), in $D_2O$ (22.5 mL initially, 25 mL total, after any other additions and $p(H,D)_{obs}$ adjustment). Initial MES buffer ($D_2O$, [MES] = 110 mM, [NaCl] = 220 mM, various $pH_{obs}$, 22.5 mL). A concentrated solution of NaOH dissolved in $D_2O$ was used to incrementally adjust the $pH_{obs}$ as monitored with a pH meter. Once the solution was within ± 0.02 pH units of the desired $pH_{obs}$, the solution was made up to a final volume of 25 mL with $D_2O$.

A standard solution of DSS was made by dissolving DSS (44 mg) in $D_2O$ MES buffer solution ($D_2O$, [DSS] = 110 mM, [MES] = 110 mM, [NaCl] = 220 mM, 1.8 mL).

A standard solution of NDA was made by dissolving NDA (166 mg) in $D_2O$ MES buffer solution ($D_2O$, [NDA] = 110 mM, [MES] = 110 mM, [NaCl] = 220 mM, 4.5 mL).

A standard solution of maleic acid **1** was made by dissolving maleic acid (232 mg) in $D_2O$ MES buffer solution ($D_2O$, [**1**] = 1.1 M, [MES] = 110 mM, [NaCl] = 220 mM, 1.8 mL).

DSS, **1**, and NDA were dried under vacuum prior to use due to their hygroscopic nature.

For intravesicular solutions (5 mL): NDA solution ($D_2O$, [NDA] = 110 mM, [MES] = 110 mM, [NaCl] = 220 mM, 1.8 mL), **1** solution ($D_2O$, [**1**] = 1.1 M, [MES] = 110 mM, [NaCl] = 220 mM, 0.090 mL) and MES buffer solution ($D_2O$, [MES] = 110 mM, [NaCl] = 220 mM, 2.6 mL) were combined and the $pH_{obs}$ adjusted with NaOD as monitored with a pH meter. The solution was made up to 5.0 mL using $D_2O$ to give the intravesicular solution ($D_2O$, [**1**] = 40 mM, [NDA] = 20 mM, [NaCl] = 200 mM, [MES] = 100 mM, $pH_{obs}$ 5.98).

Due to an error during the $pH_{obs}$ adjustment, the final intravesicular solution used in the main text (Figure 3) was ($D_2O$, [**1**] = 38 mM, [NDA] = 19 mM, [NaCl] = 200 mM, [MES] = 100 mM, $pH_{obs}$ 6.0).

For extravesicular solutions (25 mL): DSS solution ($D_2O$, [DSS] = 110 mM, [MES] = 110 mM, [NaCl] = 220 mM, 0.56 mL) and MES buffer solution ($D_2O$, [MES] = 110 mM, [NaCl] = 220 mM, 21.9 mL) were combined and the $pH_{obs}$ adjusted with NaOD as monitored with a pH meter. The solution was made up to 25 mL using $D_2O$ to give the extravesicular solution ($D_2O$, [DSS] = 2.5 mM, [NaCl] = 200 mM, [MES] = 100 mM, $pH_{obs}$ 5.61).

For deuterated MES buffer solutions to be used for uncatalyzed rate experiments (5 mL): DSS solution ($D_2O$, [DSS] = 110 mM, [MES] = 110 mM, [NaCl] = 220 mM, 0.11 mL) and MES buffer solution ($D_2O$, [MES] = 110 mM, [NaCl] = 220 mM, 4.39 mL) were combined and the $pH_{obs}$ adjusted with NaOD as monitored with a pH meter. The solutions were made up to 5.0 mL using $D_2O$ to give the uncatalyzed buffer solutions ($D_2O$, [DSS] = 2.5 mM, [NaCl] = 200 mM, [MES] = 100 mM, $pH_{obs}$ 5.21, 5.59, 5.99, 6.41, 6.80).

For deuterated MES buffer solutions to be used for catalyzed rate experiments (5 mL): **1** solution ($D_2O$, [**1**] = 1.1 M, [MES] = 110 mM, [NaCl] = 220 mM, 0.023 mL), DSS solution ($D_2O$, [DSS] = 110 mM, [MES] = 110 mM, [NaCl] = 220 mM, 0.11 mL) and MES buffer solution ($D_2O$, [MES] = 110 mM, [NaCl] = 220 mM, 4.37 mL) were combined and the $pH_{obs}$ adjusted with NaOD as monitored with a pH meter. The solutions were made up to 5.0 mL using $D_2O$ to give the catalyzed buffer solutions ($D_2O$, [**1**] = 5.0 mM, [DSS] = 2.5 mM, [NaCl] = 200 mM, [MES] = 100 mM, $pH_{obs}$ 5.19, 5.59, 6.01, 6.41, 6.79). MES buffer solutions containing **1** for pH curve calibration were made up in the same manner for a wider pH range.

### S3.2.2 General Preparation of LUV Solutions

DOPC lipid (30 mg, 0.038 mmol) was dissolved in chloroform and reduced under vacuum to form an even film of lipid which was then dried thoroughly under high vacuum for at least 6 hours. The lipid was rehydrated with the intravesicular buffer solution ($D_2O$, [**1**] = 38 mM, [NDA] = 19 mM, [NaCl] = 200 mM, [MES] = 100 mM, $pH_{obs}$ 6.0) and vortexed to ensure full saturation. The lipid suspension was subjected to 7 freeze-thaw cycles to prevent multilayer formation, flash-freezing in an acetone-dry ice bath (−78 °C) and thawing in a water bath (50 °C). The mixture was extruded 25 times through a polycarbonate membrane (pore size 200 nm). Extravesicular components were removed with size exclusion chromatography using a Cytivia PD–10 size-exclusion column eluted with the extravesicular buffer solution ($D_2O$, [DSS] = 2.5 mM, [NaCl] = 200 mM, [MES] = 100 mM, $pH_{obs}$ 5.6) and the collected LUV suspension diluted with additional extravesicular buffer solution to give the LUV suspension ([DOPC] = 12 mM, intravesicular solution: $D_2O$, [$\mathbf{1_{int}}$]$_0$ = 38 mM, [NDA] = 19 mM, [NaCl] = 200 mM, [MES] = 100 mM, $pH_{obs}$ 6.0; extravesicular solution: $D_2O$, [$1_{ext}$]$_0$ = 0 mM, [DSS] = 2.5 mM, [NaCl] = 200 mM, [MES] = 100 mM, $pH_{obs}$ 5.6).

### S3.2.3 General $^1$H NMR Vesicle Experiment Protocol

Freshly prepared LUV suspension (0.7 mL, [DOPC] = 12 mM, intravesicular solution: $D_2O$, [$\mathbf{1_{int}}$]$_0$ = 38 mM, [NDA] = 19 mM, [NaCl] = 200 mM, [MES] = 100 mM, $pH_{obs}$ 6.0; extravesicular solution: $D_2O$, [$\mathbf{1_{ext}}$]$_0$ = 0 mM, [DSS] = 2.5 mM, [NaCl] = 200 mM, [MES] = 100 mM, $pH_{obs}$ 5.6) was added to an NMR tube and $^1$H NMR spectra were acquired over time to assess the leakage of **1** across the lipid bilayer membrane. *t* = 0 was set as the first drop of vesicle solution collected from the size exclusion column for all experiments and the assumption has been made that there was no change to the extravesicular and intravesicular solutions until the LUVs began to elute. The time point for each $^1$H NMR spectrum was taken from the acquisition time metadata for each spectrum.

Once vesicles were assessed to have leaked to acceptable point (at least 9 h), active transport was initiated by the addition of EDC·MeI (20 µL, 3.6 M, [EDC·MeI] = 100 mM). The active transport of **1** was then followed by $^1$H NMR spectroscopy.

All $^1$H NMR spectra (Section S3.1) were referenced to DSS at 0.00 ppm, in addition to being the reference integral for species in the extravesicular solution. The NDA peak at 8.40 ppm was used as the reference integral for the intravesicular solution. Peaks at 6.17 ppm ($\mathbf{1_{ext}}$) and the range 6.11–6.07 ($\mathbf{1_{int}}$) were integrated through line fitting to a Lorentzian–Gaussian function. All other relevant peaks (7.24 ppm (**2**), 1.21 ppm (EDC·MeI), 1.07 ppm (EDU·MeI), and the reference species) were integrated normally over a consistent width for each spectrum.

The relative concentrations of the intravesicular species were calibrated from the internal standard NDA and the relative concentrations of the extravesicular species were calibrated from the internal standard DSS, then normalised relative to the number of nuclei pertaining to each signal. The integrals of $\mathbf{1_{int}}$, $\mathbf{1_{ext}}$, **2**, EDC·MeI, and EDU·MeI were calibrated using Equation S24, where I, N, and C are the integral area, number of nuclei, and concentration of the compound of interest (x) and the calibrant (cal), respectively.

$$C_x = \frac{I_x}{I_{cal}} \times \frac{N_{cal}}{N_x} \times C_{cal} \quad \text{(Equation S24)}$$

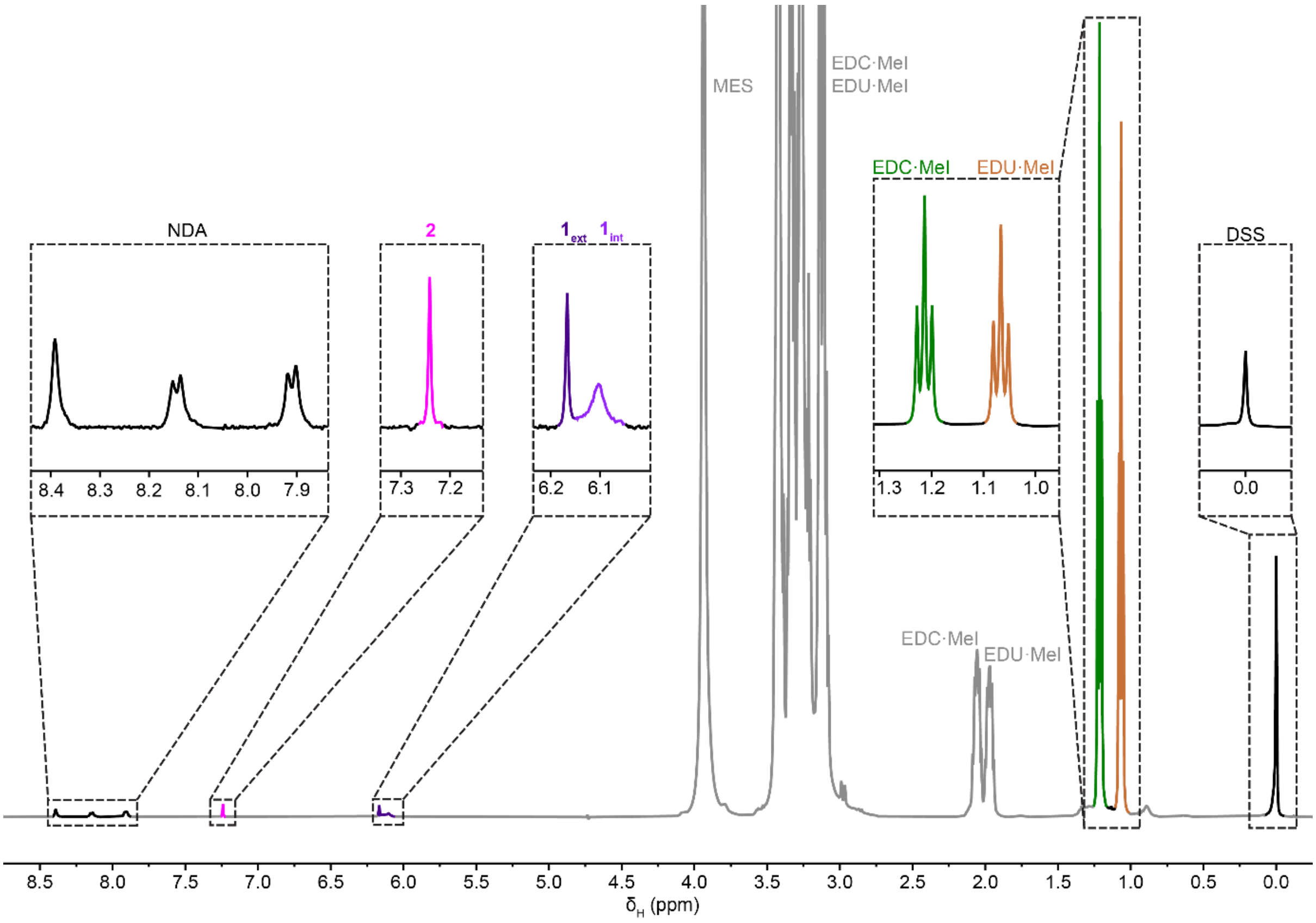


**Figure S10.** General example of the $^1$H NMR (500 MHz, 298 K, $D_2O$, Section S.31) spectra obtained during fueling experiments of LUV solutions (0.7 mL, 200 nm LUVs, [DOPC] = 12 mM, intravesicular solution: $D_2O$, $[\mathbf{1}_{int}]_0$ = 38 mM, [NDA] = 19 mM, [NaCl] = 200 mM, [MES] = 100 mM, $pH_{obs}$ 6.0; extravesicular solution: $D_2O$, $[\mathbf{1}_{ext}]_0$ = 0 mM, [DSS] = 2.5 mM, [NaCl] = 200 mM, [MES] = 100 mM, $pH_{obs}$ 5.6). The region 8.5–6.0 ppm is scaled vertically 40× compared to the region 1.3–0.0 ppm.

### S3.2.4 General $^1$H NMR Non-Vesicle Experiment Protocol

A pre-fuelled spectrum was obtained to assess the initial concentrations for *t* = 0 s. EDC·MeI (10 µL, 3.6 M, [EDC·MeI] = 50 mM) was added to initiate the experiment at *t* = 0 s. $^1$H NMR spectra were obtained every ~8 min for at least 10 spectra, after which more spectra were obtained at longer intervals, depending on the concentration of EDC·MeI still visible.

All $^1$H NMR spectra (Section S3.1) were referenced to DSS at 0.00 ppm, in addition to being the reference integral for the solution. Peaks at 7.24 ppm (**2**), in the range 6.30–6.00 (**1**), 1.21 ppm (EDC·MeI), 1.07 ppm (EDU·MeI), and the reference species at 0.00 ppm (DSS) were integrated normally over a consistent width for each spectrum. The relative concentrations of each species were calculated with respect to the integral of DSS using Equation S24.

## S3.3. $^{1}$H NMR Quantitation and $T_1$ Relaxation Constants

To ensure the integrals were correct, the repetition time was set as the acquisition time added to 5 times the longest $T_1$ constant of the sample.

A sample of freshly prepared LUV solution (0.7 mL, [DOPC] = 20 mM, intravesicular solution: $H_2O/D_2O$ (9:1 *v*/*v*), [**1**$_{int}$]$_0$ = 50 mM, [NDA] = 20 mM, [NaCl] = 200 mM, [MES] = 100 mM, $pH_{obs}$ 5.5; extravesicular solution: $H_2O/D_2O$ (9:1 *v*/*v*), [**1**$_{ext}$]$_0$ = 0 mM, [DSS] = 5.0 mM, [NaCl] = 200 mM, [MES] = 100 mM, $pH_{obs}$ 5.5) was monitored by inversion recovery $^{1}$H NMR (Section 3.1, Figure S11). A series of spectra with the inversion recovery delay time (τ) varied were obtained and the integrals of the relevant peaks plotted against τ. The resulting plot follows Equation S25, where $M_0$ is the net magnetization vector prior to irradiating, and was fitted in MestreNova to a three-parameter exponential fit (Equation S26, where B = $M_0$, F = −2$M_0$ and G = 1/$T_1$). The constants calculated are shown in Table S3 and the value of each resonance's $T_1$ is therefore 1/G, as highlighted (Table S3, blue).

$$M(\tau) = M_0\left(1 - 2e^{-\tau/T_1}\right) \qquad \text{(Equation S25)}$$

$$\mathrm{y = B + Fe^{-Gx}} \qquad \text{(Equation S26)}$$

**Table S3.** Parameters associated with $T_1$ measurements

| Species | Integral Range | B | F | G | Total Error | $T_1$ / s |
|---|---|---|---|---|---|---|
| NDA | 8.60→8.30 | 235 ± 3 | −454 ± 4 | 0.3702 ± 0.01 | 12.86 | 2.7 |
| **1** | 6.69→6.02 | 2940 ± 40 | −5750 ± 50 | 0.214 ± 0.005 | 139.1 | 4.7 |
| DSS | 0.30→−0.30 | 7840 ± 70 | − 15290 ± 90 | 0.483 ± 0.009 | 305.2 | 2.1 |

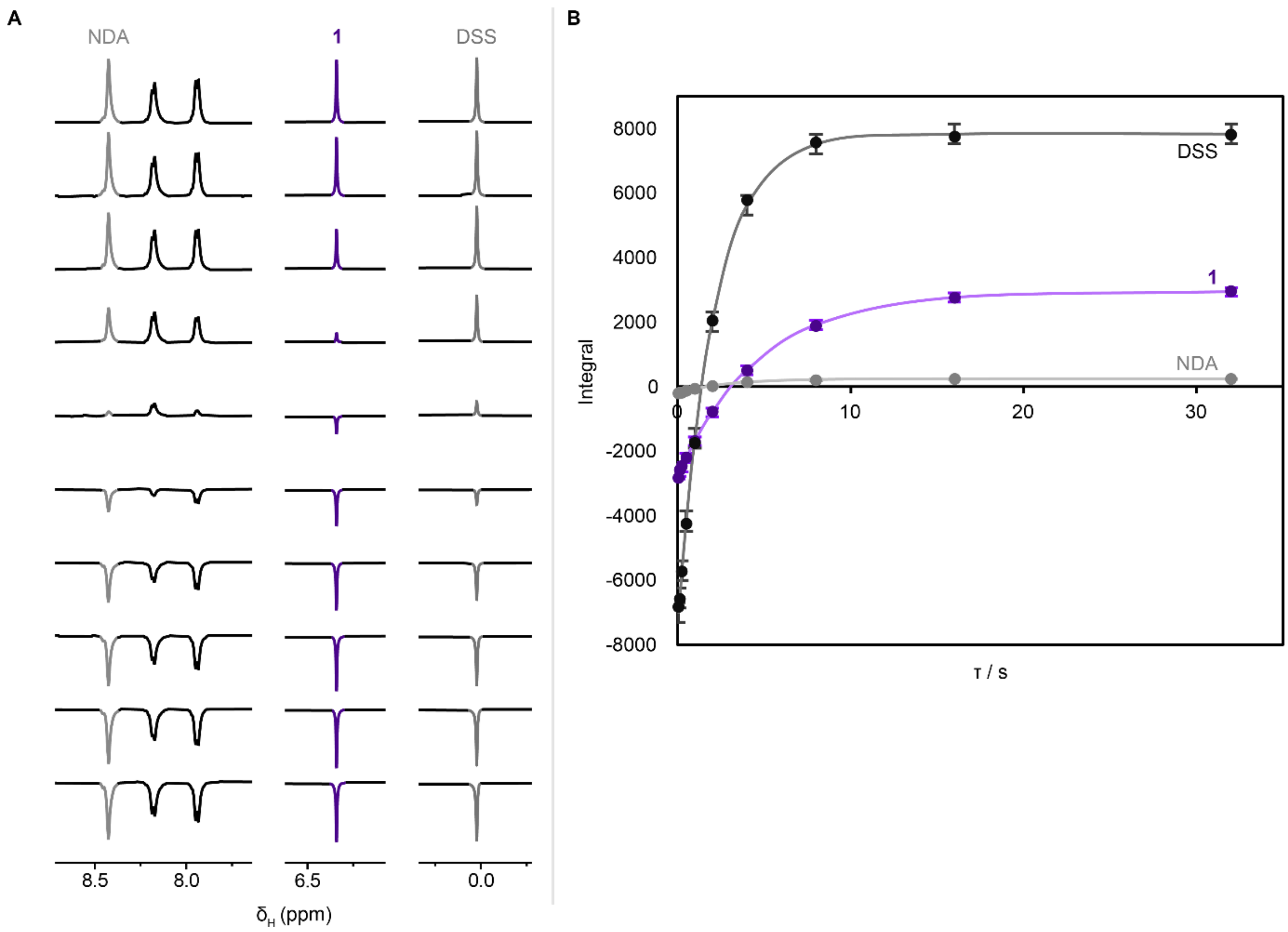


**Figure S11.** Inversion recovery using Robust-5 pulse sequence (Section 3.1) $^{1}$H NMR for calculating the $T_1$ relaxation constants. **A** Partial $^{1}$H NMR (600 MHz, 298 K, $H_2O/D_2O$ (9:1 *v*/*v*)) stack plot of LUV solution (0.7 mL, 200 nm LUVs, [DOPC] = 20 mM, intravesicular solution: $H_2O/D_2O$ (9:1 *v*/*v*), [$\mathbf{1}_{\mathbf{int}}$]$_0$ = 50 mM, [NDA] = 20 mM, [NaCl] = 200 mM, [MES] = 100 mM, $pH_{obs}$ 5.5; extravesicular solution: $H_2O/D_2O$ (9:1 *v*/*v*), [$\mathbf{1}_{\mathbf{ext}}$]$_0$ = 0 mM, [DSS] = 5.0 mM, [NaCl] = 200 mM, [MES] = 100 mM, $pH_{obs}$ 5.5) highlighting the resonances followed. The region 8.5–6.0 ppm is scaled vertically 40× compared to the region 0.5–−0.5 ppm. **B** Plot of the integral of the relevant resonances against τ. The solid lines represent the fit to Equation S26 and errors were plotted from the total error values (Table S3) given in MestreNova.

For all $^{1}$H NMR experiments using the Robust-5 solvent suppression sequence (Section 3.1), a repetition time of 3.3 + (5 × 4.7) = 26.8 s was used. The $T_1$ constants for EDC·MeI and EDU·MeI and **2** were not obtained in this method as these species would not have constant integrals under the relevant conditions.

## S3.4. pH Calibration Curve

Solutions of **1** in MES buffer were prepared (Section S3.2) to produce a series of solutions ($D_2O$, [**1**] = 5.0 mM, [DSS] = 2.5 mM, [NaCl] = 200 mM, [MES] = 100 mM) with the $pH_{obs}$ ranging from 2.2–8.0. The chemical shift of **1** was read relative to the chemical shift of DSS at 0.00 ppm. The plot of $\delta_H$ against $pH_{obs}$ could be fitted to a general sigmodal formula of the form Equation S27, where a, b, c, and d are constants that were allowed to be fitted using the Excel Solver function. The constants a–d were found to be a = 0.382, b = −1.77, c = −10.0, and d = 5.98. Rearranging Equation S27 produced Equation S28 which allowed the pH of the intravesicular and extravesicular compartments to be followed from the $^1H$ NMR spectra (Figure S12).

$$\delta_H = \frac{a}{1+e^{(-b\cdot pH_{obs}+c)}} + d \qquad \text{(Equation S27)}$$

$$pH = \frac{ln\left(\frac{0.382}{\delta_H - 5.98} - 1\right) + 10.0}{1.77} \qquad \text{(Equation S28)}$$

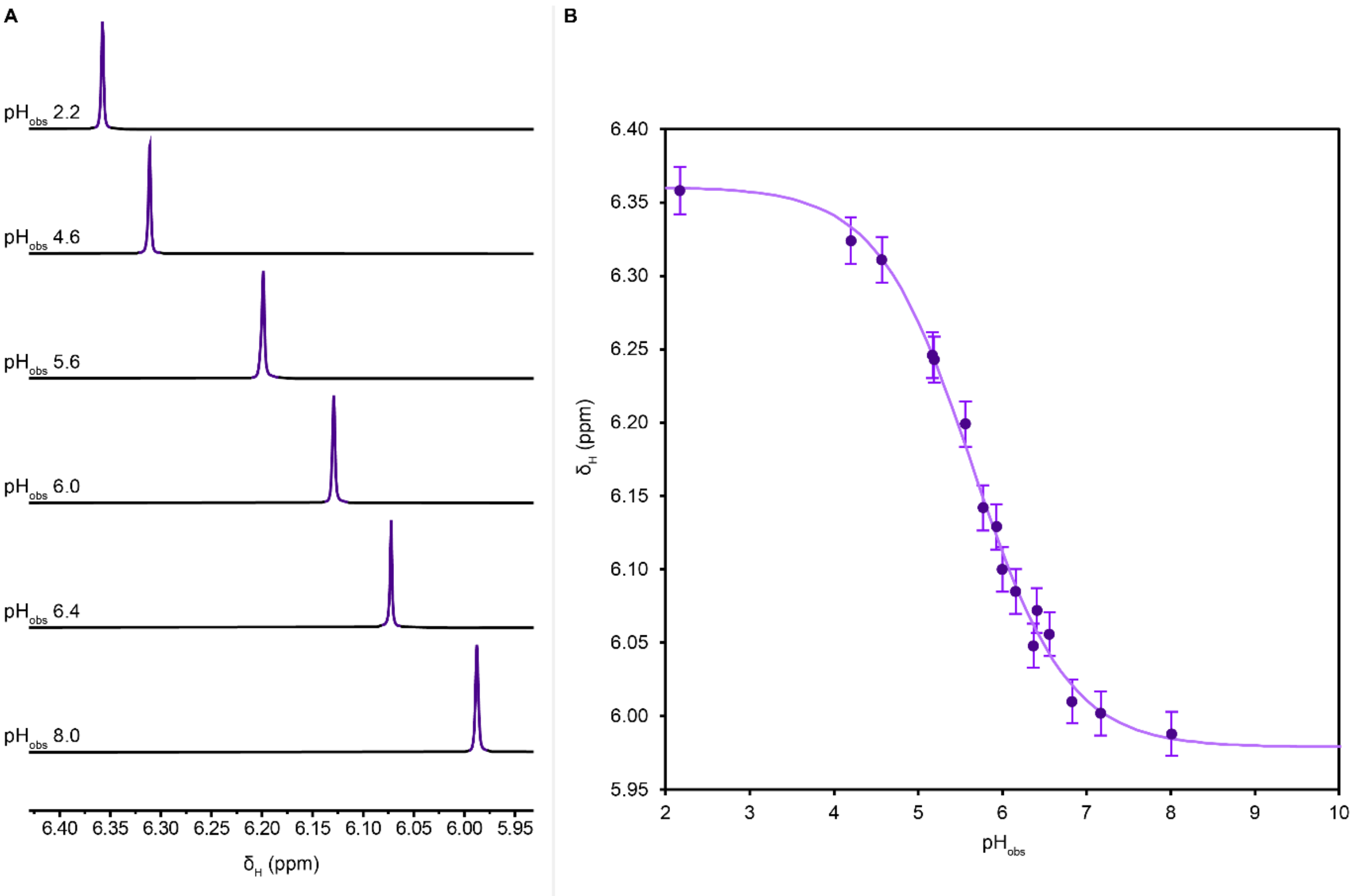


**Figure S12.** pH dependence of **1** in MES-buffered solutions. **A** Selected partial $^1H$ NMR stack plot ($D_2O$, 500 MHz, 298 K, [**1**] = 5 mM, [NaCl] = 200 mM, [MES buffer] = 100 mM, [DSS] = 2.5 mM) relative to DSS showing the chemical shift of **1** at various $pH_{obs}$. **B** The sigmodal profile observed for the chemical shift of **1**. Solid line represents the fit to Equation S27. Errors are set to a standard of 0.25% of the observed chemical shift.

## S3.5. Active Transport Experiments

Monitoring active transport by $^{1}H$ NMR spectroscopy requires balancing of several factors:

1. Intravesicular and extravesicular pH must be sufficiently different (>0.3 pH units) to allow $\mathbf{1}_{int}$ and $\mathbf{1}_{ext}$ to be resolved.

2. The intravesicular pH should be as high as possible to increase the proportion of doubly deprotonated $\mathbf{1}_{int}$, and thus reduce the rate of leakage.

3. An extravesicular $pH_{obs} \approx 5.5$ was required to ensure that the reaction proceeded at a convenient rate for observing the anhydride formation by $^{1}H$ NMR spectroscopy.

4. Finally, the NMR peaks for $\mathbf{1}_{int}$ and $\mathbf{1}_{ext}$ come in a similar region to the urea NH peaks, so steps must be taken to allow integration and minimize overlap.

After much optimization, the above considerations resulted in the following standard experimental conditions for active transport experiments as described in Section S3.2.

Active transport experiments were performed in duplicate (Figure S13, S14) on the same batch of LUVs and produced results in good agreement. There was slight variation due to the repeat experiment being performed on vesicles which had been able to leak for ~1.5 h more than the initial ones as active transport experiments could not be followed simultaneously on two samples by $^{1}H$ NMR spectroscopy.

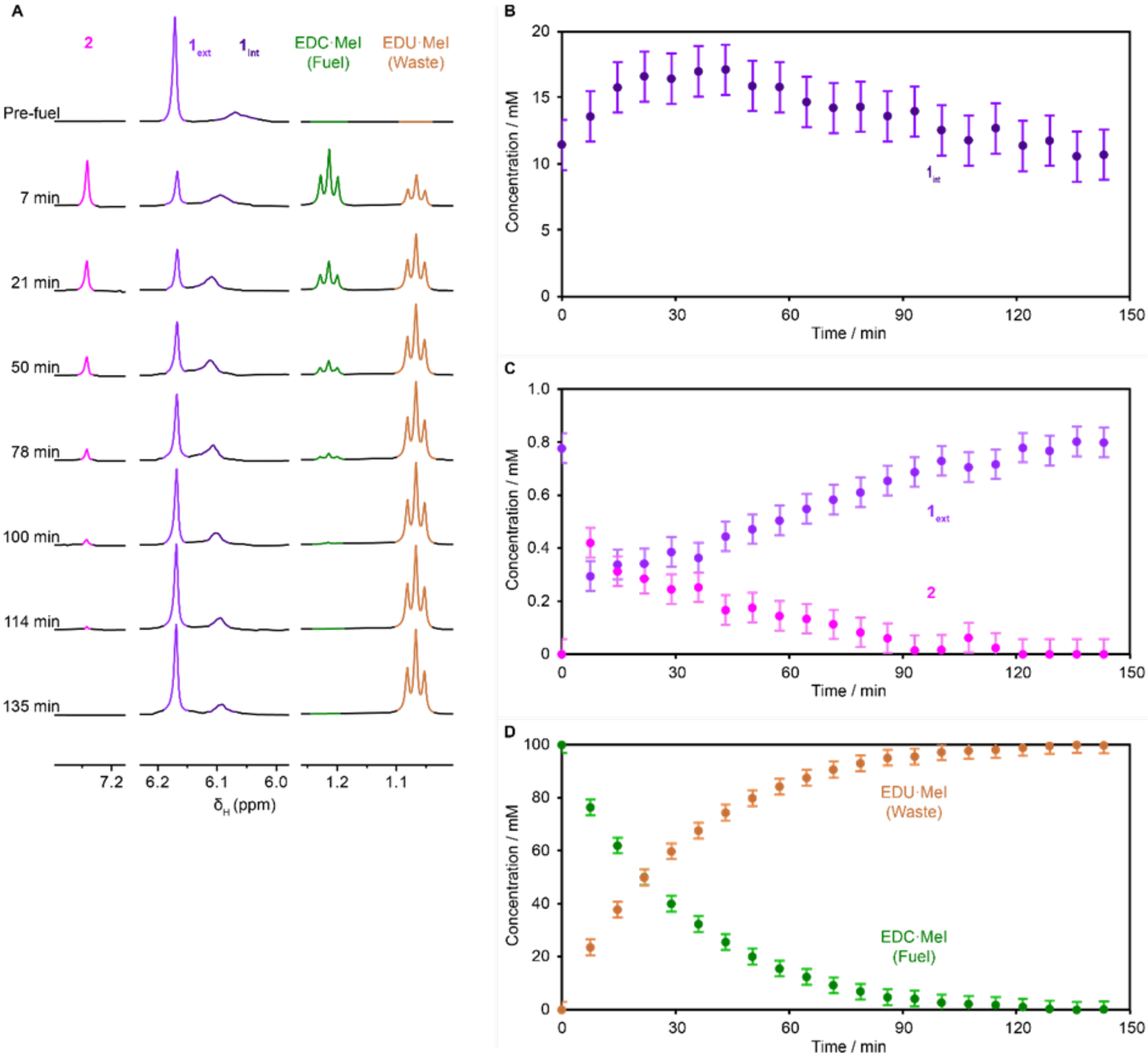


**Figure S13.** In situ monitoring of active transport upon fueling a suspension of pseudo-equilibrated vesicles (0.7 mL, 200 nm LUVs, DOPC = 12 mM, [$\mathbf{1}_{\mathbf{int}}$]$_0$ = 12 mM, [NDA] = 19 mM, [NaCl] = 200 mM, [MES buffer] = 100 mM, pH$_{obs}$ 6.0; extravesicular solution: [$\mathbf{1}_{\mathbf{ext}}$]$_0$ = 0.8 mM, [EDC·MeI]$_0$ = 100 mM, [DSS] = 2.5 mM, [NaCl] = 200 mM, [MES buffer] = 100 mM, pH$_{obs}$ 5.6) by $^1$H NMR spectroscopy. This active transport experiment was run before the data shown in the main text (Figure 3), hence the noticeably lower concentration of $\mathbf{1}_{\mathbf{ext}}$. **A** Partial selected $^1$H NMR ($D_2O$, 500 MHz, 298 K) stack plot showing the change in concentration of $\mathbf{1}_{\mathbf{ext}}$, $\mathbf{1}_{\mathbf{int}}$, **2**, EDC·MeI and EDU·MeI (ethyl triplet peaks) upon fueling. **B** Concentrations of $\mathbf{1}_{\mathbf{ext}}$, $\mathbf{1}_{\mathbf{int}}$, and **2** over time. **C** Expansion of previous plot showing the concentrations of $\mathbf{1}_{\mathbf{ext}}$ and **2** more clearly. Error bars represent 5% of the total concentration of $\mathbf{1}_{\mathbf{int}}$, $\mathbf{1}_{\mathbf{ext}}$ and **2**. **D** Concentrations of EDC·MeI and EDU·MeI over time. Error bars represent 3% of the total concentration of EDC·MeI and EDU·MeI.

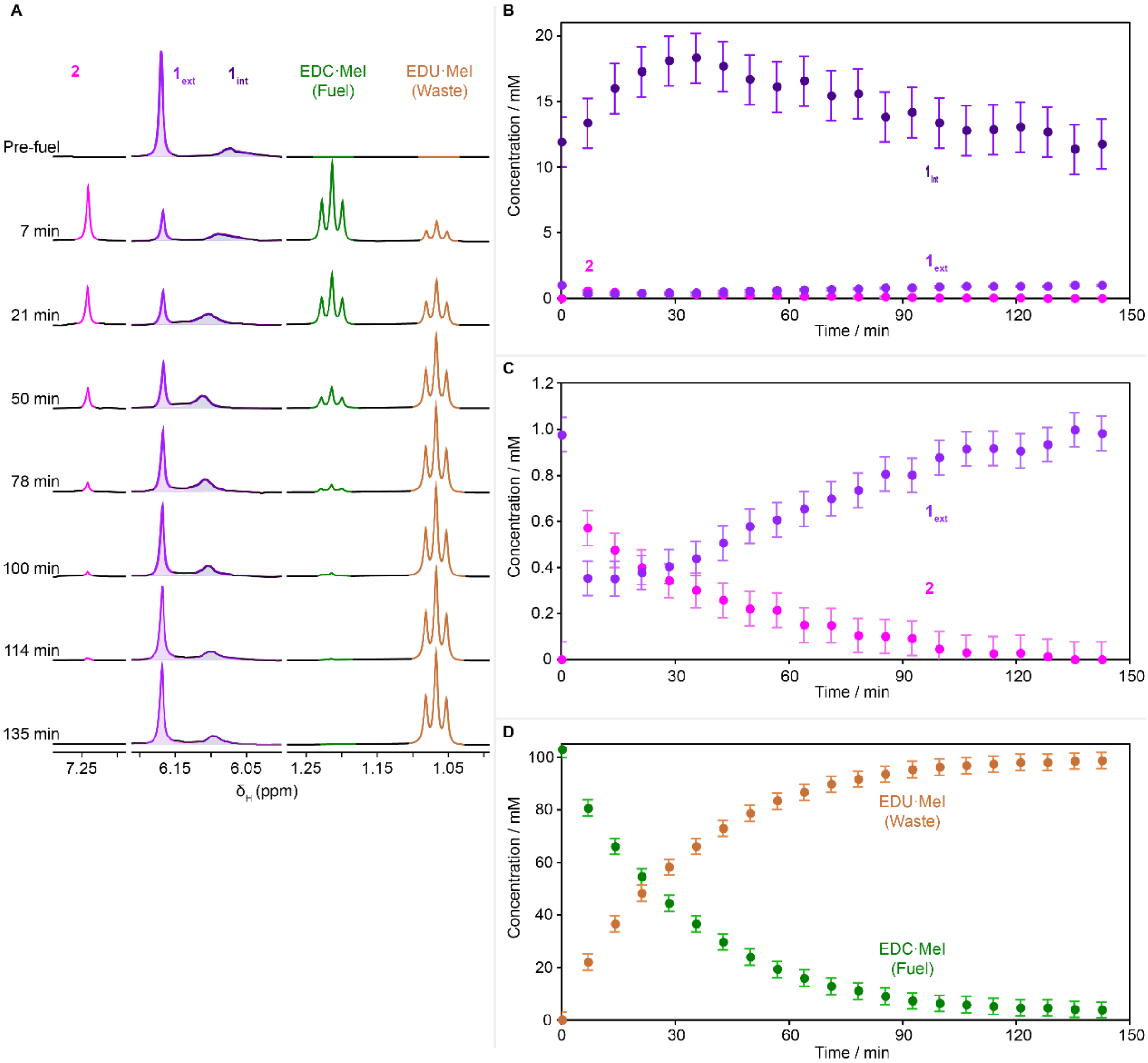


**Figure S14.** In situ monitoring of active transport upon fueling a suspension of pseudo-equilibrated vesicles (0.7 mL, 200 nm LUVs, DOPC = 12 mM, [$\mathbf{1_{int}}$]$_0$ = 11 mM, [NDA] = 19 mM, [NaCl] = 200 mM, [MES buffer] = 100 mM, pH$_{obs}$ 6.0; extravesicular solution: [$\mathbf{1_{ext}}$]$_0$ = 1.0 mM, [EDC·MeI]$_0$ = 100 mM, [DSS] = 2.5 mM, [NaCl] = 200 mM, [MES buffer] = 100 mM, pH$_{obs}$ 5.6) by $^1$H NMR spectroscopy. This is the data contained within the main text (Figure 3). **A** Partial selected $^1$H NMR ($D_2O$, 500 MHz, 298 K) stack plot showing the change in concentration of $\mathbf{1_{ext}}$, $\mathbf{1_{int}}$, **2**, EDC·MeI and EDU·MeI (ethyl triplet peaks) upon fueling. **B** Concentrations of $\mathbf{1_{ext}}$, $\mathbf{1_{int}}$, and **2** over time. Error bars represent 5% of the total concentration of $\mathbf{1_{int}}$, $\mathbf{1_{ext}}$ and **2**. **C** Expansion of previous plot showing the concentrations of $\mathbf{1_{ext}}$ and **2** more clearly. **D** Concentrations of EDC·MeI and EDU·MeI over time. Error bars represent 3% of the total concentration of EDC·MeI and EDU·MeI.

A comparable active transport experiment using a separate batch of LUVs (200 nm LUVs, [DOPC] = 12 mM, intravesicular solution: $D_2O$, [**1**$_{int}$]$_0$ ≈ 3 mM, [NDA] = 20 mM, [NaCl] = 200 mM, [MES buffer] = 100 mM, pH$_{obs}$ 6.0; extravesicular solution: $D_2O$, [**1**$_{ext}$]$_0$ ≈ 2 mM, [EDC·MeI]$_0$ = 100 mM, [DSS] = 2.5 mM, [NaCl] = 200 mM, [MES buffer] = 100 mM, pH$_{obs}$ 5.6) was performed. These LUVs started with lower concentrations of **1**$_{int}$ and were fueled with less EDC·MeI (Figure S15).

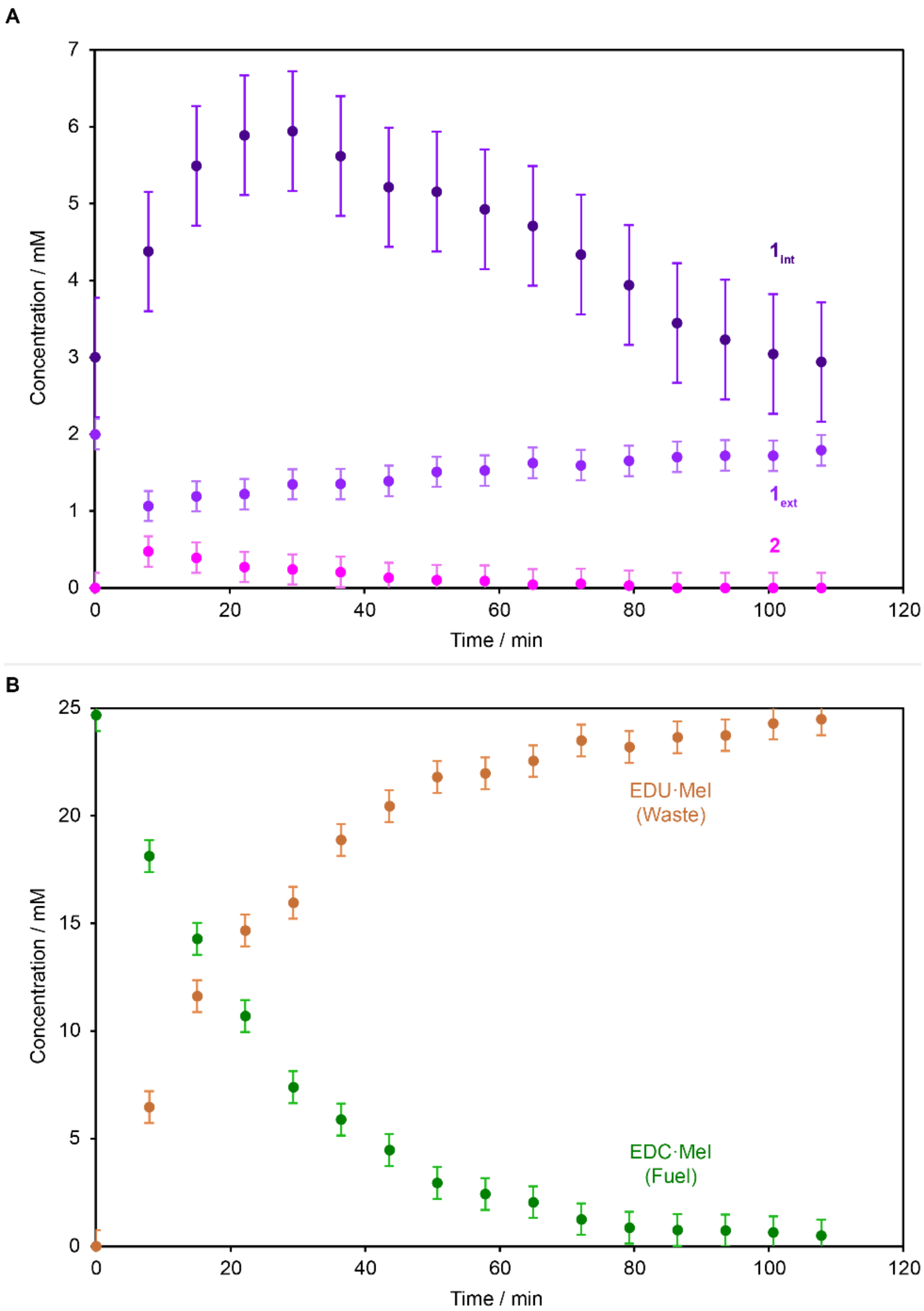


**Figure S15.** In situ monitoring of active transport in LUVs (200 nm LUVs, [DOPC] = 12 mM, intravesicular solution: $D_2O$, [**1**$_{int}$]$_0$ ≈ 3 mM, [NDA] = 20 mM, [NaCl] = 200 mM, [MES buffer] = 100 mM, pH$_{obs}$ 6.0; extravesicular solution: $D_2O$, [**1**$_{ext}$]$_0$ ≈ 2 mM, [EDC·MeI]$_0$ = 25 mM, [DSS] = 2.5 mM, [NaCl] = 200 mM, [MES buffer] = 100 mM, pH$_{obs}$ 5.6) by $^1$H NMR ($D_2O$, 500 MHz, 298 K) spectroscopy. **A** Concentration of **1**$_{int}$, **1**$_{ext}$ and **2** during the active transport experiment. Data points for **1**$_{int}$ and **1**$_{ext}$ at *t* = 0 s were estimates taken. Error bars represent 5% of the total concentration of **1**$_{int}$, **1**$_{ext}$ and **2**. **B** Concentration of EDC·MeI fuel and EDU·MeI waste during the active transport experiment. Error bars represent 3% of the total concentration of EDC·MeI and EDU·MeI.

### S3.5.1 Generation of a pH gradient

As diacid **1** is pumped inside the vesicles, a change in the chemical shift of the $\mathbf{1}_{\mathbf{int}}$ resonance is visible. This can be followed and converted to a pH using Equation S28. There is a slight increase in the pH of the extravesicular solution due to the addition of EDC·MeI being slightly basic. The initial intravesicular solution was at $pH_{obs}$ 6.0; however, as diacid **1** leaked out prior to fueling, the $pH_{obs}$ immediately before fueling was actually pH ~6.3. Upon fueling, the intravesicular $pH_{obs}$ drops to pH ~6.0 during the pumping, before slowly increasing again as the rate of leakage of **1** starts to dominate.

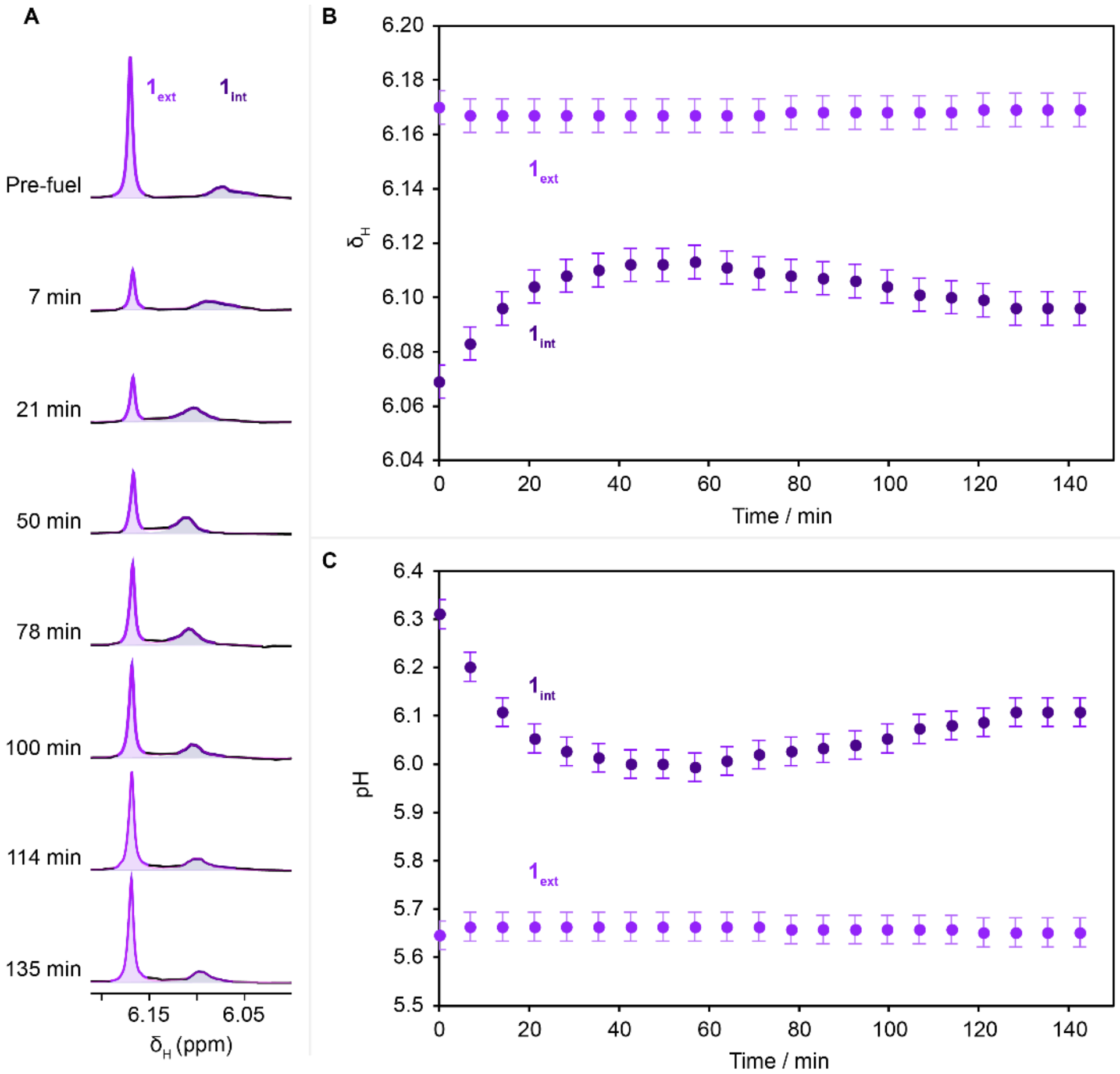


**Figure S16.** The change in pH of the intravesicular and extravesicular compartments can be followed using the resonances of **1** in $^1$H NMR. **A** Selected partial $^1$H NMR (stack plot $D_2O$, 500 MHz, 298 K) of LUV solution (0.7 mL, 200 nm LUVs, [DOPC] = 12 mM, $[\mathbf{1}_{\mathbf{int}}]_0$ = 11 mM, [NDA] = 19 mM, [NaCl] = 200 mM, [MES buffer] = 100 mM, $pH_{obs}$ 6.0; extravesicular solution: $[\mathbf{1}_{\mathbf{ext}}]_0$ = 1.0 mM, $[\text{EDC·MeI}]_0$ = 100 mM, [DSS] = 2.5 mM, [NaCl] = 200 mM, [MES buffer] = 100 mM, $pH_{obs}$ 5.6) showing the downfield shift of $\mathbf{1}_{int}$ during the fueling experiment, and subsequent upfield shift as $\mathbf{1}_{\mathbf{int}}$ leaks out again. **B** The change in $\delta_H$ of the resonances of $\mathbf{1}_{\mathbf{int}}$ and $\mathbf{1}_{\mathbf{ext}}$ with time. Error bars represent 0.1% of the $\delta_H$ observed. **C** The change in pH of $\mathbf{1}_{\mathbf{int}}$ and $\mathbf{1}_{\mathbf{ext}}$ with time as calculated using Equation S28. Error bars represent ±0.03 pH units.

### S3.5.2 Correlation of Intravesicular Concentration and pH

As **1** is pumped into the vesicles, the pH decreases. Plotting the pH vs the [$\mathbf{1}_{int}$] reveals two separate linear regimes. A steeper gradient (Figure S17, light purple) as the active transport occurs, followed by a shallower gradient (Figure S17, dark purple) as **1** leaks out of the vesicles; the ratio of the gradients is 2:1. This hysteresis is consistent with the different mechanism of transport during active transport and leakage. As the anhydride is hydrolyzed in the intravesicular solution during active transport, protons on one water molecule are converted to acidic protons on **1**; each molecule of **1** transported generates 2× $H^+$. The leakage likely occurs by leakage of the mono-protonated version of **1** (since the doubly protonated version is inaccessible at the experimental pH values); each molecule of **1** that leaks transports 1× $H^+$.

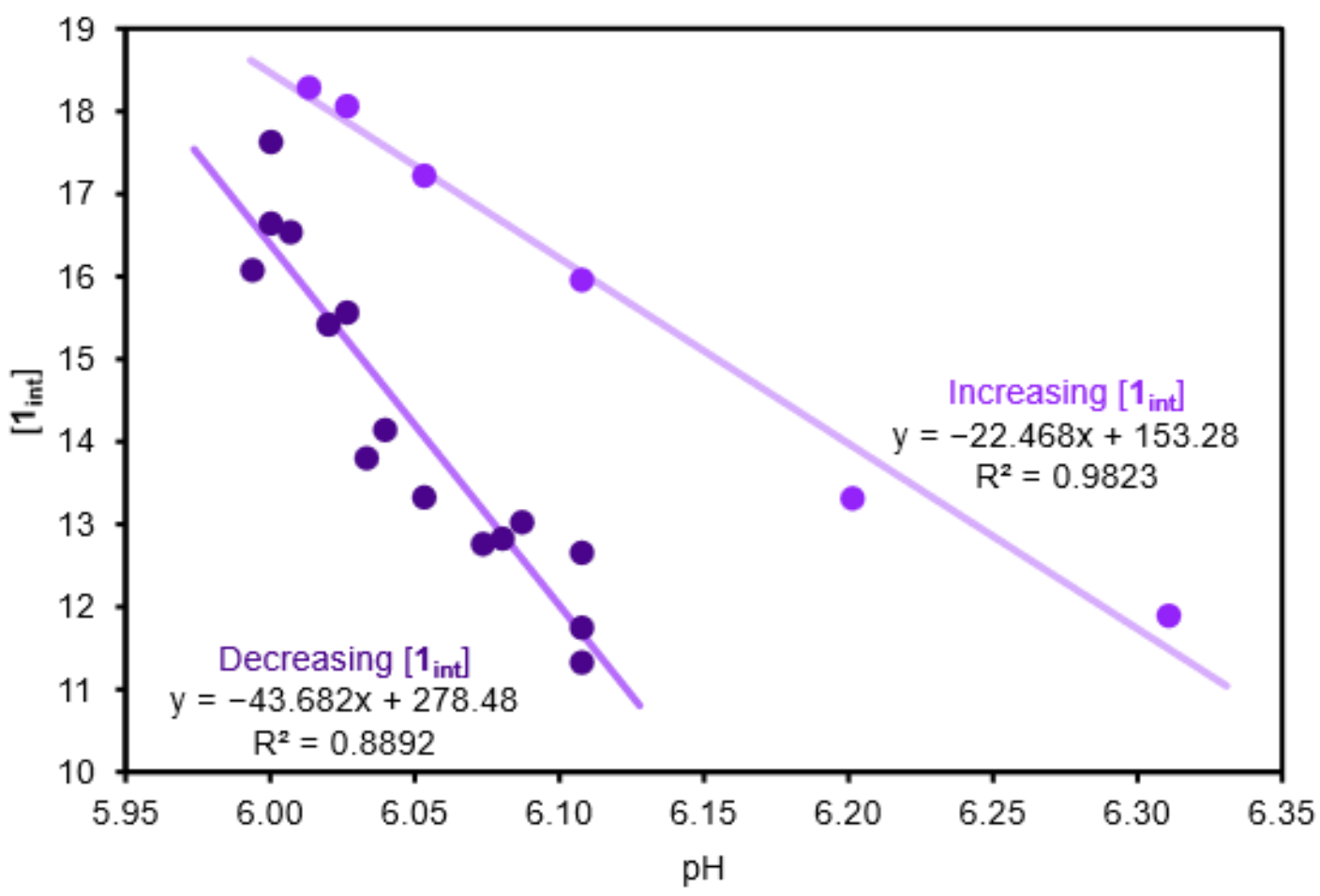


**Figure S17.** Plot of [$\mathbf{1}_{int}$] against pH during active transport experiment in LUVs (0.7 mL, 200 nm LUVs, [DOPC] = 12 mM, [$\mathbf{1}_{int}$]$_0$ = 11 mM, [NDA] = 19 mM, [NaCl] = 200 mM, [MES buffer] = 100 mM, $pH_{obs}$ 6.0; extravesicular solution: [$\mathbf{1}_{ext}$]$_0$ = 1.0 mM, [EDC·MeI]$_0$ = 100 mM, [DSS] = 2.5 mM, [NaCl] = 200 mM, [MES buffer] = 100 mM, $pH_{obs}$ 5.6). Errors are set to standard errors of ±0.03 pH units horizontally, and 5% of the total concentration of $\mathbf{1}_{int}$, $\mathbf{1}_{ext}$ and **2** vertically, but have been removed for clarity.

## S3.6. Vesicle Calculations

### S3.6.1 Calculating the Intravesicular Volume from Literature Values

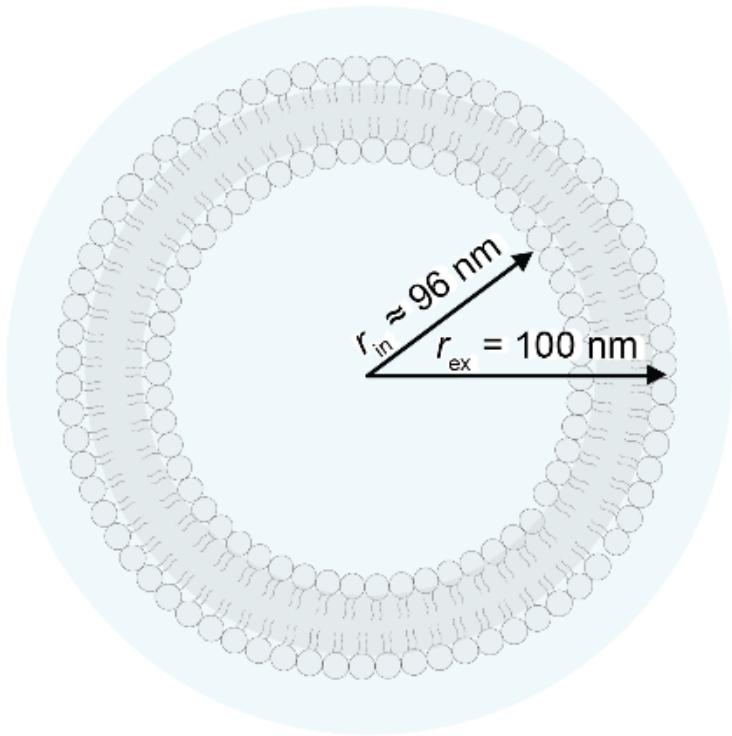


**Figure S18.** Schematic representation of a 200 nm diameter LUV.

A literature calculation[S6] was followed to find the overall intravesicular volume based on DOPC LUVs with a mean diameter of 200 nm (Figure S18). The parameters used for this calculation follow.

Overall radius of the LUV exterior, $r_{ex}$ = 100 nm.

Thickness of DOPC lipid bilayer = 3.71 nm.[S7a–h] Hence the radius of the interior volume, $r_{in}$ ≈ 96 nm

Overall volume, $V_{\mathrm{ext}} = \frac{4}{3}\pi r_{\mathrm{ext}}^3$ = 4.189 × $10^{-12}$ μL

Interior volume, $V_{\mathrm{int}} = \frac{4}{3}\pi r_{\mathrm{int}}^3$ = 3.739 × $10^{-12}$ μL

Bilayer membrane volume, $V_{\mathrm{mem}} = V_{\mathrm{ext}} - V_{\mathrm{int}}$ = 4.494 × $10^{-13}$ μL

Exterior lipid bilayer surface area, $SA_{\mathrm{ext}} = 4\pi r_{\mathrm{ext}}^2$ = 1.257 × $10^5$ $\mathrm{nm}^2$

Interior lipid bilayer surface area, $SA_{\mathrm{int}} = 4\pi r_{\mathrm{int}}^2$ = 1.165 × $10^5$ $\mathrm{nm}^2$

Since the cross-sectional area per DOPC molecule ($A_{DOPC}$) from literatures[S7a–h] = 0.7021 $\mathrm{nm}^2$

Number of DOPC molecules on the exterior leaflet, $DOPC_{ext}$ = $SA_{\mathrm{ext}} \div A_{\mathrm{DOPC}}$ = 1.790 × $10^5$

Number of DOPC molecules on the interior leaflet, $DOPC_{int}$ = $SA_{\mathrm{int}} \div A_{\mathrm{DOPC}}$ = 1.659 × $10^5$

Total number of DOPC molecules per LUV, $DOPC_{total}$ = $DOPC_{int}$ + $DOPC_{ext}$ = 3.449 × $10^5$.

NMR studies were conducted with LUV suspensions with a lipid concentration ($C_{DOPC}$) of 12 mM; hence the concentration of LUVs, $C_{LUV}$ = $C_{DOPC}$ ÷ $DOPC_{total}$ = 3.479 × $10^{-5}$ mM

Fraction of interior volume to total volume, $\%V_{int}$ = $C_{LUV}$ × $V_{in}$ × $N_A$ = 7.83%

Fraction of exterior bulk volume to total volume, $\%V_{ext}$ = 1 – ($C_{LUV}$ × $V_{in}$ × $N_A$) = 92.17%.

Hence, the total intravesicular volume, $V_{int}$ = 5.484 × $10^{-5}$ L and the total extravesicular volume, $V_{ext}$ = 6.452 × $10^{-4}$ L.

The volume conversion factor (the number the intravesicular volume would have to be multiplied by to give the extravesicular volume), $V_{c.f.}$ = 92.17% ÷ 7.83% = 11.76.

Avogadro constant, $N_A = 6.022 \times 10^{23}$ mol$^{-1}$.

Values used above for the lipid bilayer thickness (Luzzati thickness)[S8] and cross-sectional area (defined as area per lipid along the surface of the bilayer) were averages from previous values determined from experimental and simulation methods in fully hydrated DOPC lipid bilayer systems, between a temperature range of 293 K to 303 K.[S7a–h]

### S3.6.2 Calculating the Intravesicular Volume from Active Transport Experiments

During the active transport experiments monitored by $^1$H NMR, an intravesicular standard (NDA) and an extravesicular standard (DSS) were also used to compare the interior and exterior volumes as the above calculations assumes no lipid is lost at any stage during the synthesis of LUVs and also does not account for the volume taken up by the lipid bilayers. Additionally, the compartmental volumes are essential for accurately modelling the kinetic system.

An estimate for the interior volume from the experimental standards was obtained by first calculating the concentration of DSS with respect to (w.r.t.) the known concentration of NDA across all spectra using Equation S24 and then taking the average of those values. This is the concentration of DSS if the integral measured was for a resonance occurring inside the vesicles. The average concentration of DSS w.r.t. NDA was divided by the known concentration of DSS in the extravesicular solution to produce a volume conversion factor ($V_{c.f.}$). The same was done for calculating the concentration of NDA w.r.t. the known concentration of DSS, also producing a $V_{c.f.}$ by dividing the known intravesicular concentration by the calculated extravesicular concentration. The average of the two values for $V_{c.f.}$ came out to be 26.8. This value is higher than the value for $V_{c.f.}$ obtained from literature-based calculations, indicating that lipid is lost during the process of making LUVs.

Using the simultaneous equations: $V_{c.f.} = \frac{V_{ext}}{V_{int}}$ ; $V_{total} = V_{int} + V_{ext} = 700$ µL, these can be rearranged to give $V_{ext} = \frac{V_{total} \times V_{c.f.}}{1+V_{c.f.}} = 6.748 \times 10^{-4}$ L, %$V_{ext}$ = 96.40%. Hence the total intravesicular volume $V_{int} = 2.517 \times 10^{-5}$ L and %$V_{int}$ = 3.60%.

This method has also assumed that the lipid volume is negligible compared to the bulk solution; however, indicates that the total intravesicular solution is smaller than initially thought.

### S3.6.3 Calculating the Intervesicular Distance

Mean inter-particle distance = $\frac{1}{\sqrt[3]{n}}$ , where n is the particle density defined as the total number of LUVs ($N_{LUV}$) per unit volume. DOPC LUV suspensions were analysed by $^1$H NMR in 0.7 mL batches ($V_{total}$).

$$N_{LUV} = \frac{DOPC_{total}}{C_{DOPC} \times V_{total} \times N_A} = 1.467 \times 10^{13}$$

The particle density, $n = \frac{N_{LUV}}{V_{total}} = 2.095 \times 10^{19}$ m$^{-3}$

Hence the mean interparticle distance = $\frac{1}{\sqrt[3]{n}}$ = 362 nm

## S3.7. Kinetic Modelling of Active Transport

### S3.7.1 The Model

Kinetic modelling was performed as per Figure S19.

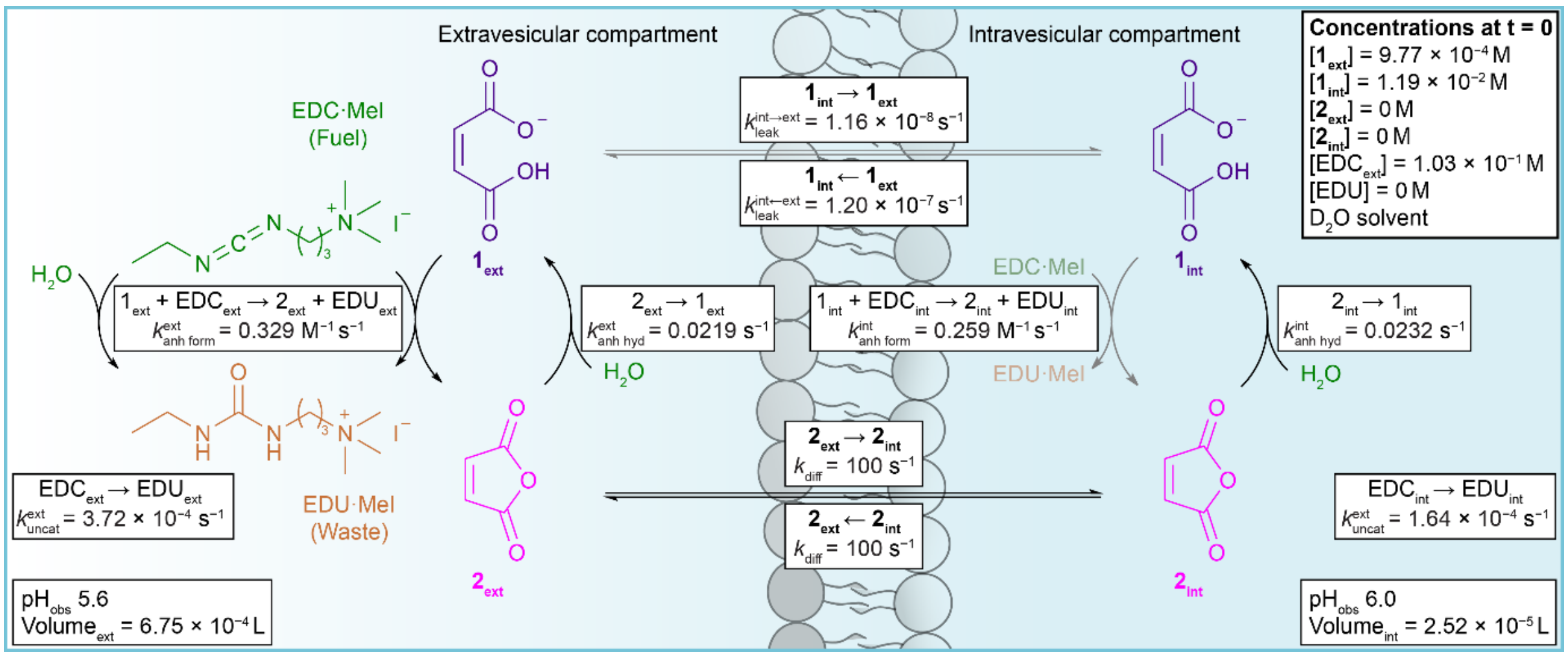


**Figure S19.** Schematic representation of the kinetic model. Microscopic reverse processes of chemical transitions are not considered since they are vanishingly small.

**Table S4.** Parameters for the kinetic model.

| Species | Intravesicular Compartment | Extravesicular Compartment |
|---|---|---|
| Volume | $2.517 \times 10^{-5}$ L | $6.748 \times 10^{-4}$ L |
| [**1**] | $1.190 \times 10^{-2}$ mM | $9.767 \times 10^{-4}$ mM |
| [**2**] | 0 mM | 0 mM |
| [EDC·MeI] | 0 mM | $1.029 \times 10^{-1}$ mM |
| [EDU·MeI] | 0 mM | 0 mM |

**Table S5.** Individually determined rate constants for kinetic model. Most rates constants were obtained from isolated experiments (Section S3.7). For the full fit, rate constants for leakage were re-fitted to account for the actual intravesicular and extravesicular pH during the experiments.

| Constant | Value from independent experiments | Value from updated fit |
|---|---|---|
| $k_{uncat}^{pH\ 5.6}$ | $3.720 \times 10^{-4}\ s^{-1}$ | $3.720 \times 10^{-4}\ s^{-1}$ |
| $k_{uncat}^{pH\ 6.0}$ | $1.490 \times 10^{-4}\ s^{-1}$ | $1.490 \times 10^{-4}\ s^{-1}$ |
| $k_{anh\ form}^{pH\ 5.6}$ | $3.489 \times 10^{-1}\ M^{-1}s^{-1}$ | $3.489 \times 10^{-1}\ M^{-1}s^{-1}$ |
| $k_{anh\ form}^{pH\ 6.0}$ | $2.591 \times 10^{-1}\ M^{-1}s^{-1}$ | $2.591 \times 10^{-1}\ M^{-1}s^{-1}$ |
| $k_{anh\ hyd}^{pH\ 5.6}$ | $2.283 \times 10^{-2}\ s^{-1}$ | $2.283 \times 10^{-2}\ s^{-1}$ |
| $k_{anh\ hyd}^{pH\ 6.0}$ | $2.889 \times 10^{-2}\ s^{-1}$ | $2.889 \times 10^{-2}\ s^{-1}$ |
| $k_{diff}^{int \to ext} = k_{diff}^{int \to ext}$ | $100\ s^{-1}$ | $100\ s^{-1}$ |
| $k_{leak}^{int \to ext}$ | $2.949 \times 10^{-9}\ s^{-1}$ | $1.161 \times 10^{-8}\ s^{-1}$ |
| $k_{leak}^{ext \to int}$ | $3.903 \times 10^{-8}\ s^{-1}$ | $1.200 \times 10^{-7}\ s^{-1}$ |

Differential equations for the kinetic model:

$$\frac{d([\mathbf{1}_{ext}]\cdot V_{ext})}{dt} = -V_{ext} \cdot (3.489 \times 10^{-1} \cdot [\mathbf{1}_{ext}] \cdot [\mathrm{EDC{\cdot}MeI}_{ext}]) + V_{ext} \cdot (2.283 \times 10^{-2} \cdot [\mathbf{2}_{ext}]) - (1.200 \times 10^{-7} \cdot [\mathbf{1}_{ext}] - 1.161 \times 10^{-8} \cdot [\mathbf{1}_{int}])$$ (Equation S29)

$$\frac{d([\mathbf{1}_{int}]\cdot V_{int})}{dt} = -V_{int} \cdot (2.591 \times 10^{-1} \cdot [\mathbf{1}_{int}] \cdot [\mathrm{EDC{\cdot}MeI}_{int}]) + V_{int} \cdot (2.889 \times 10^{-2} \cdot [\mathbf{2}_{int}]) + (1.200 \times 10^{-7} \cdot [\mathbf{1}_{ext}] - 1.161 \times 10^{-8} \cdot [\mathbf{1}_{int}])$$ (Equation S30)

$$\frac{d([\mathbf{2}_{ext}]\cdot V_{ext})}{dt} = -V_{ext} \cdot (3.489 \times 10^{-1} \cdot [\mathbf{1}_{ext}] \cdot [\mathrm{EDC{\cdot}MeI}_{ext}]) - V_{ext} \cdot (2.283 \times 10^{-2} \cdot [\mathbf{2}_{ext}]) - (1 \times 10^{2} \cdot [\mathbf{2}_{ext}] - 1 \times 10^{2} \cdot [\mathbf{2}_{int}])$$ (Equation S31)

$$\frac{d([\mathbf{2}_{int}]\cdot V_{int})}{dt} = +V_{int} \cdot (2.591 \times 10^{-1} \cdot [\mathbf{1}_{int}] \cdot [\mathrm{EDC{\cdot}MeI}_{int}]) - V_{int} \cdot (2.889 \times 10^{-2} \cdot [\mathbf{2}_{int}]) + (1 \times 10^{2} \cdot [\mathbf{2}_{ext}] - 1 \times 10^{2} \cdot [\mathbf{2}_{int}])$$ (Equation S32)

$$\frac{d([\mathrm{EDC}_{ext}]\cdot V_{ext})}{dt} = -V_{ext} \cdot (3.720 \times 10^{-4} \cdot [\mathrm{EDC{\cdot}MeI}_{ext}]) - V_{ext} \cdot (3.489 \times 10^{-1} \cdot [\mathbf{1}_{ext}] \cdot [\mathrm{EDC{\cdot}MeI}_{ext}])$$ (Equation S33)

$$\frac{d([\mathrm{EDC}_{int}]\cdot V_{int})}{dt} = -V_{ext} \cdot (2.591 \times 10^{-1} \cdot [\mathbf{1}_{int}] \cdot [\mathrm{EDC{\cdot}MeI}_{int}])$$ (Equation S34)

$$\frac{d([\mathrm{EDU}_{ext}]\cdot V_{ext})}{dt} = +V_{ext} \cdot (3.720 \times 10^{-4} \cdot [\mathrm{EDC{\cdot}MeI}_{ext}]) + V_{ext} \cdot (3.489 \times 10^{-1} \cdot [\mathbf{1}_{ext}] \cdot [\mathrm{EDC{\cdot}MeI}_{ext}]) \quad \text{(Equation S35)}$$

$$\frac{d([\mathrm{EDU}_{int}]\cdot V_{int})}{dt} = +V_{int} \cdot (2591 \times 10^{-4} \cdot [\mathrm{EDC{\cdot}MeI}_{ext}]) \quad \text{(Equation S36)}$$

Instead of fitting the model to the active transport data, which would result in over-fitting, individual experiments were run to independently derive the rate constants for each step of the catalytic cycle.

### S3.7.2 Quantifying the Rate of Uncatalyzed EDC·MeI Hydration

The uncatalyzed hydration of EDC·MeI to form EDU·MeI was followed by $^{1}$H NMR (Section S3.2). EDC·MeI (10 μL, 1.42 M) was added to an NMR tube of MES-buffered solution (0.70 mL, $D_2O$, $[\mathrm{EDC{\cdot}MeI}]_0$ = 20 mM, [MES] = 100 mM, [NaCl] = 200 mM, [DSS] = 2.5 mM, $pH_{obs}$ 5.6 or 6.0) to initiate the experiment. The ethyl triplet peaks of EDC·MeI and EDU·MeI were integrated relative to each other in order to follow the reaction. The rate of uncatalyzed EDC·MeI hydration was fitted to the pseudo-first order rate equation (Equation S37) to obtain the rate constants for each $pH_{obs}$.

$$\frac{d[\mathrm{EDC{\cdot}MeI}]}{dt} = -k_{uncat}^{pH_{obs}}[\mathrm{EDC{\cdot}MeI}] \quad \text{(Equation S37)}$$

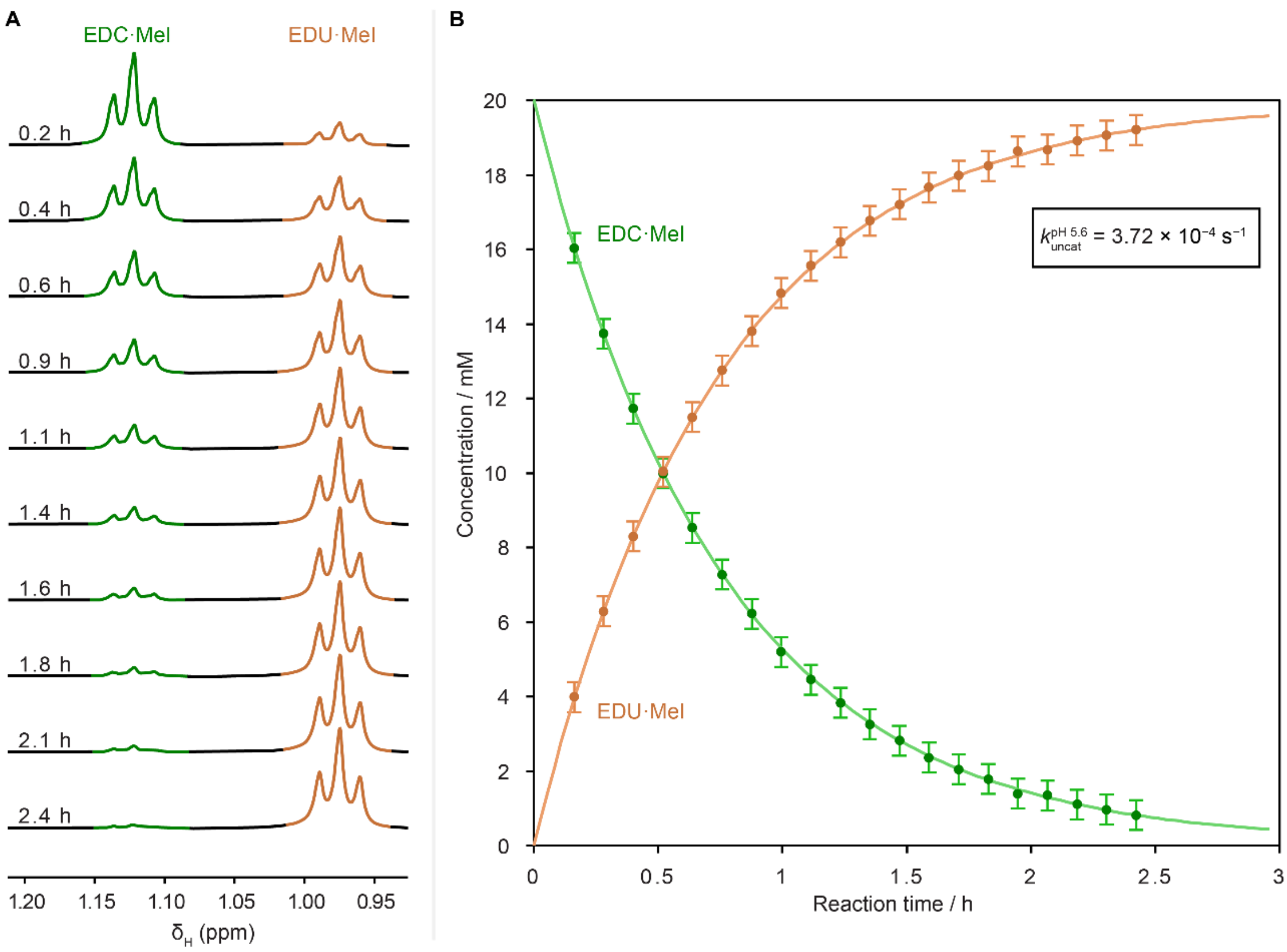


**Figure S20.** Kinetics of uncatalyzed EDC·MeI fuel consumption at $pH_{obs}$ 5.6 ([EDC·MeI] = 20.0 mM, [NaCl] = 200 mM, [MES] = 100 mM, $pH_{obs}$ 5.6). **A** Selected partial $^{1}H$ NMR ($D_2O$, 500 MHz, 298 K) stack plot showing the disappearance of EDC·MeI fuel and appearance of EDU·MeI waste (ethyl $CH_3$ signals). **B** Kinetics of carbodiimide hydration in the absence of maleic acid catalyst, determined by $^{1}H$ NMR spectroscopy. Solid lines represent the fit to pseudo-first order kinetics ($k_{uncat}$). Error bars represent standard integral errors of 3% of the total concentration of EDC·MeI and EDU·MeI.

Experiments were run in duplicate and the average rate of the individually calculated fits used. An individual run and fit are shown with the average rate in Figure S20.

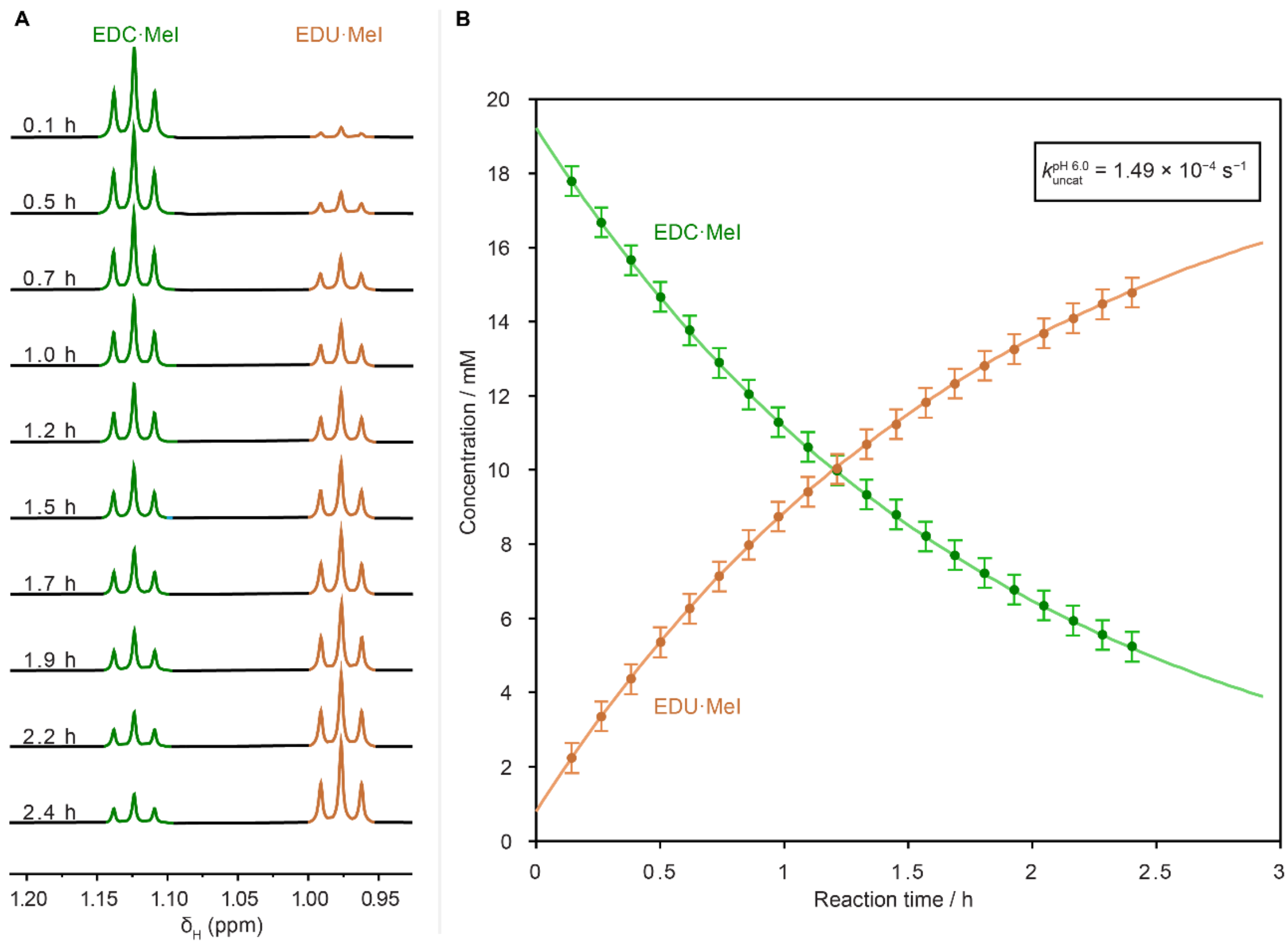


**Figure S21.** Kinetics of uncatalysed EDC·MeI hydration at $pH_{obs}$ 6.0 ([EDC·MeI] = 20.0 mM, [NaCl] = 200 mM, [MES] = 100 mM, $pH_{obs}$ 6.0). **A** Selected partial $^1$H NMR ($D_2O$, 500 MHz, 298 K) stack plot showing the disappearance of EDC·MeI fuel and appearance of EDU·MeI waste (ethyl $CH_3$ signals). **B** Kinetics of carbodiimide hydration in the absence of catalyst **1**, determined by $^1$H NMR spectroscopy. Solid lines represent the fit to pseudo-first order kinetics ($k_{uncat}$). Error bars represent standard integral errors of 3% of the total concentration of EDC·MeI and EDU·MeI.

Experiments were run in duplicate and the average rate of the individually calculated fits used. An individual run and fit are shown with the average rate in Figure S21.

### S3.7.3 Quantifying the Rate of Catalyzed EDC·MeI Hydration

Using the previously obtained uncatalyzed rates of EDC·MeI hydration, the catalyzed rate could be quantified. The **1** catalyzed hydration of EDC·MeI was followed by $^1$H NMR (Section S3.2). EDC·MeI (10 µL, 3.55 M) was added to an NMR tube of MES-buffered **1** solution (0.71 mL, $D_2O$, $[EDC{\cdot}MeI]_0$ = 50 mM, [**1**] = 4.9 mM, [MES] = 100 mM, [NaCl] = 200 mM, [DSS] = 2.5 mM, $pH_{obs}$ 6.0) to initiate the experiment. The catalyzed hydration at $pH_{obs}$ 5.6 with 50 mM EDC·MeI proceeded too quickly to be followed by $^1$H NMR so $[EDC{\cdot}MeI]_0$ was doubled (0.72 mL, $D_2O$, $[EDC{\cdot}MeI]_0$ = 100 mM, [**1**] = 4.9 mM, [MES] = 100 mM, [NaCl] = 200 mM, [DSS] = 2.5 mM, $pH_{obs}$ 5.6). The ethyl triplet peaks of EDC·MeI and EDU·MeI were integrated relative to each other and the resonances for **1** and **2** were integrated relative to each other in order to follow the reaction. The rate of catalyzed EDC·MeI hydration was fitted using the following equations:

$$\frac{d[\mathbf{1}]}{dt} = -k_{anh\,form}^{pH_{obs}}[\mathbf{1}][\mathrm{EDC{\cdot}MeI}] + k_{anh\,hyd}^{pH_{obs}}[\mathbf{2}] \quad \text{(Equation S38)}$$

$$\frac{d[\mathbf{2}]}{dt} = k_{anh\,form}^{pH_{obs}}[\mathbf{1}][\mathrm{EDC{\cdot}MeI}] - k_{anh\,hyd}^{pH_{obs}}[\mathbf{2}] \quad \text{(Equation S39)}$$

$$\frac{d[EDC\cdot MeI]}{dt} = -k^{pH_{obs}}_{anh\,form}[\mathbf{1}][\mathrm{EDC{\cdot}MeI}] - k^{pH_{obs}}_{uncat}[\mathrm{EDC{\cdot}MeI}] \qquad \text{(Equation S40)}$$

$$\frac{d[\mathrm{EDU{\cdot}MeI}]}{dt} = k^{pH_{obs}}_{anh\,form}[\mathbf{1}][\mathrm{EDC{\cdot}MeI}] + k^{pH_{obs}}_{uncat}[\mathrm{EDC \cdot MeI}] \qquad \text{(Equation S41)}$$

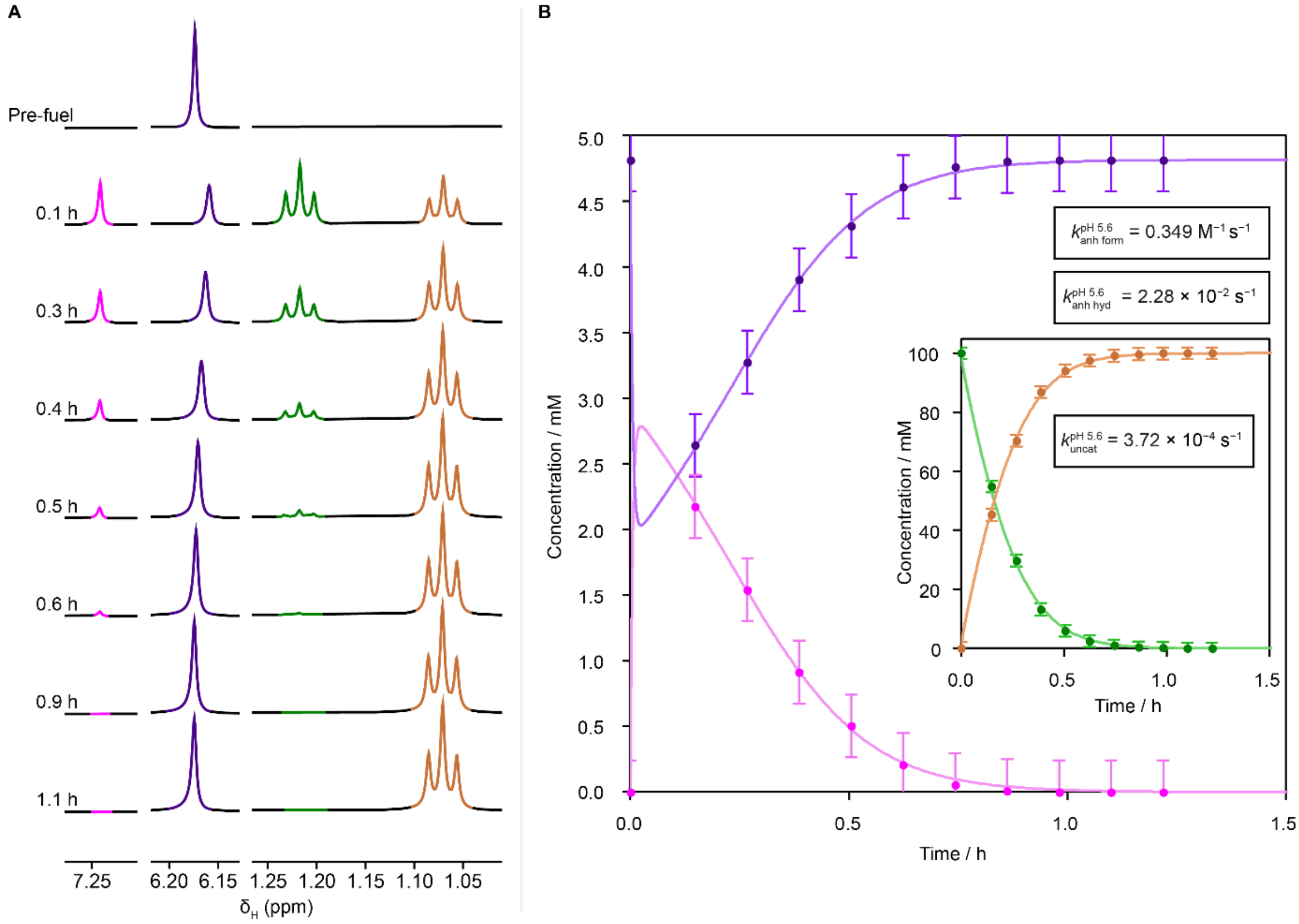


**Figure S22.** Kinetics of catalyzed EDC·MeI hydration at $pH_{obs}$ 5.6 (0.72 mL, $D_2O$, $[EDC{\cdot}MeI]_0$ = 100 mM, [**1**] = 4.9 mM, [MES] = 100 mM, [NaCl] = 200 mM, [DSS] = 2.5 mM, $pH_{obs}$ 5.6). **A** Selected partial $^1H$ NMR ($D_2O$, 500 MHz, 298 K) stack plot showing the conversion of **1** into **2** and subsequent hydrolysis back to **1** and the disappearance of EDC·MeI fuel and appearance of EDU·MeI waste (ethyl $CH_3$ signals). **B** Kinetics of carbodiimide hydration in the presence of catalyst **1** at $pH_{obs}$ 5.6, determined by $^1H$ NMR spectroscopy. Solid lines represent the fit to the model determining $k^{pH\,6.0}_{anh\,form}$ and $k^{pH\,6.0}_{anh\,hyd}$. Error bars represent standard integral errors of 5% the total concentration of **1** and **2** (large graph) and 3% of the total concentration of EDC·MeI and EDU·MeI (small graph).

Experiments were run in duplicate and the average rate of the individually calculated fits used. An individual run and fit are shown with the average rate in Figure S22.

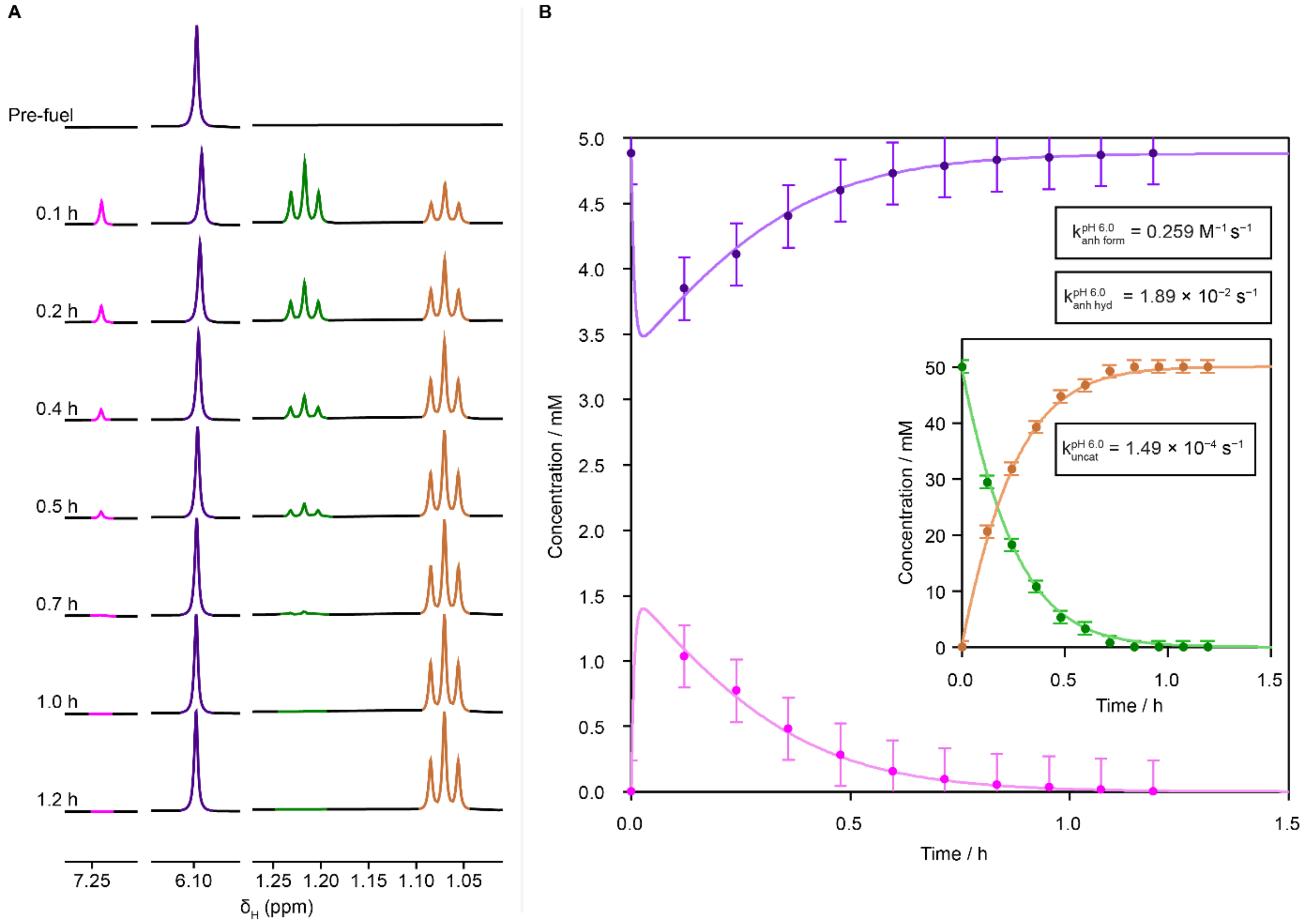


**Figure S23.** Kinetics of catalyzed EDC·MeI hydration at $pH_{obs}$ 6.0 (0.71 mL, $D_2O$, [EDC·MeI]$_0$ = 50 mM, [**1**] = 4.9 mM, [MES] = 100 mM, [NaCl] = 200 mM, [DSS] = 2.5 mM, $pH_{obs}$ 6.0). **A** Selected partial $^1$H NMR ($D_2O$, 500 MHz, 298 K) stack plot showing the conversion of **1** into **2** and subsequent hydrolysis back to **1** and the disappearance of EDC·MeI fuel and appearance of EDU·MeI waste (ethyl $CH_3$ signals). **B** Kinetics of carbodiimide hydration in the presence of catalyst **1** at $pH_{obs}$ 6.0, determined by $^1$H NMR spectroscopy. Solid lines represent the fit to the model determining $k_{anh\,form}^{pH\,6.0}$ and $k_{anh\,hyd}^{pH\,6.0}$. Error bars represent standard integral errors of 5% the total concentration of **1** and **2** (large graph) and 3% of the total concentration of EDC·MeI and EDU·MeI (small graph).

Experiments were run in duplicate and the average rate of the individually calculated fits used. An individual run and fit are shown with the average rate in Figure S23.

### S3.7.4 Quantifying the Rate of Leakage Across the Lipid Bilayer

Freshly made LUV suspension (0.7 mL, [DOPC] = 12 mM, intravesicular solution: $D_2O$, [$\mathbf{1}_{int}$]$_0$ = 38 mM, [NDA] = 19 mM, [NaCl] = 200 mM, [MES] = 100 mM, $pH_{obs}$ 6.0; extravesicular solution: $D_2O$, [$1_{ext}$]$_0$ = 0 mM, [DSS] = 2.5 mM, [NaCl] = 200 mM, [MES] = 100 mM, $pH_{obs}$ 5.6, Section S3.2) was immediately followed by $^1$H NMR over a period of ~9 h. The equilibration and leakage of **1** from the intravesicular solution to the extravesicular solution was followed and fitted to the following equilibrium:

$$k_{leak}^{int\rightarrow ext} \rightleftharpoons k_{leak}^{ext\rightarrow int} \qquad \text{(Equation S42)}$$

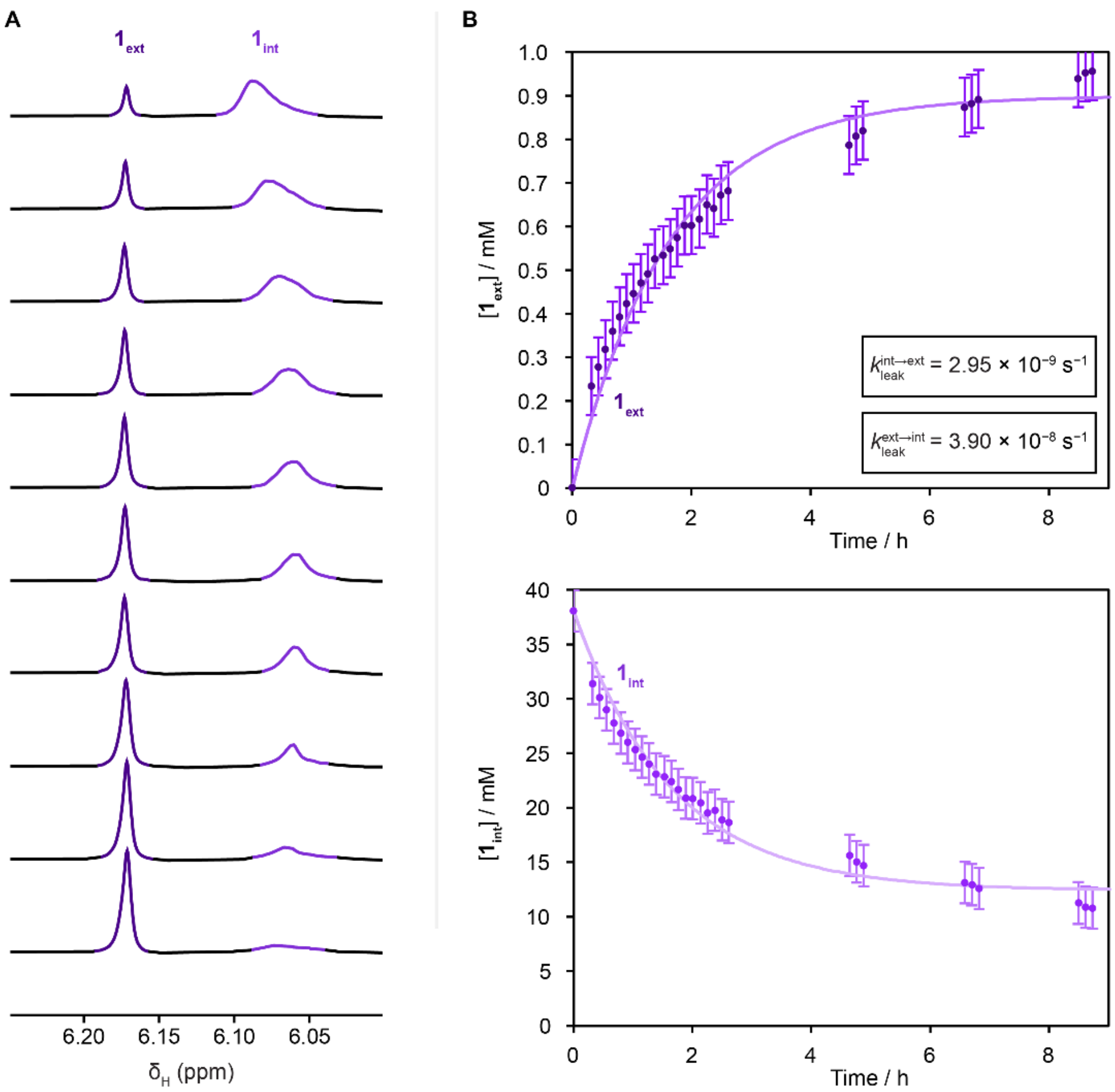


**Figure S24.** Kinetics of **1** leakage across phospholipid bilayer in LUVs (0.7 mL, 200 nm LUVs, [DOPC] = 12 mM, intravesicular solution: $D_2O$, [$\mathbf{1}_{int}$]$_0$ = 38 mM, [NDA] = 19 mM, [NaCl] = 200 mM, [MES] = 100 mM, $pH_{obs}$ 6.0; extravesicular solution: $D_2O$, [$\mathbf{1}_{ext}$]$_0$ = 0 mM, [DSS] = 2.5 mM, [NaCl] = 200 mM, [MES] = 100 mM, $pH_{obs}$ 5.6). **A** Selected partial $^1$H NMR stack plot ($D_2O$, 500 MHz, 298 K) showing the increase of $\mathbf{1}_{ext}$ and decrease of $\mathbf{1}_{int}$ in LUV solution. **B** Psuedo-first order equilibrium kinetics of the leakage of **1** across the membrane, determined by $^1$H NMR spectroscopy. Solid lines represent the fit to a pseudo-first order equilibrium kinetics, $k_{leak}^{int\rightarrow ext}$ and $k_{leak}^{ext\rightarrow int}$ . Error bars represent standard integral errors of 5% of the total concentration of $\mathbf{1}_{int}$ and $\mathbf{1}_{ext}$.

Experiments were run in duplicate and the average rate of the individually calculated fits used. An individual run and fit are shown with the average rate in Figure S24.

The leakage rates were fitted to a pseudo-equilibrium. As expected, the fitted pseudo-equilibrium shows a lower rate of leakage from the intravesicular compartment to the extravesicular compartment due to the higher intravesicular pH, as a greater proportion of $\mathbf{1}_{int}$ is in its di-anionic form, reducing its membrane permeability. The pseudo-equilibrium fit shown does not fit perfectly due to the changing pH, especially in the intravesicular compartment, during the leakage.

### S3.7.5 Full Kinetic Model Using Independently Derived Rate Constants

Using the individually determined rate constants for each step of the catalytic cycle, a model could be produced to describe the pumping of **1** across the lipid bilayer membranes as set out in Section S3.7.1. The kinetic model using the independently derived rate constants has been overlaid with the experimental data in Figure S25. During the active transport, the

concentration of acid inside the vesicle changes, hence the pH also changes (See Section S3.5). As the rate of leakage across the lipid bilayer is pH dependent, using the pseudo-first order leakage rate obtained when the vesicles are first made (Section S3.7.4) results in the concentrations of $\mathbf{1}_{int}$ being over-predicted and $\mathbf{1}_{ext}$ being under-predicted.

As $\mathbf{2}_{int}$ and $\mathbf{2}_{ext}$ cannot be distinguished by $^{1}$H NMR, the kinetic plot for **2** as a whole has been shown, instead of the separate outputs given by the modelling.

The normalised root-mean-square error (See Section S2.4) was calculated over 105 data points and found to be 16% for this model.

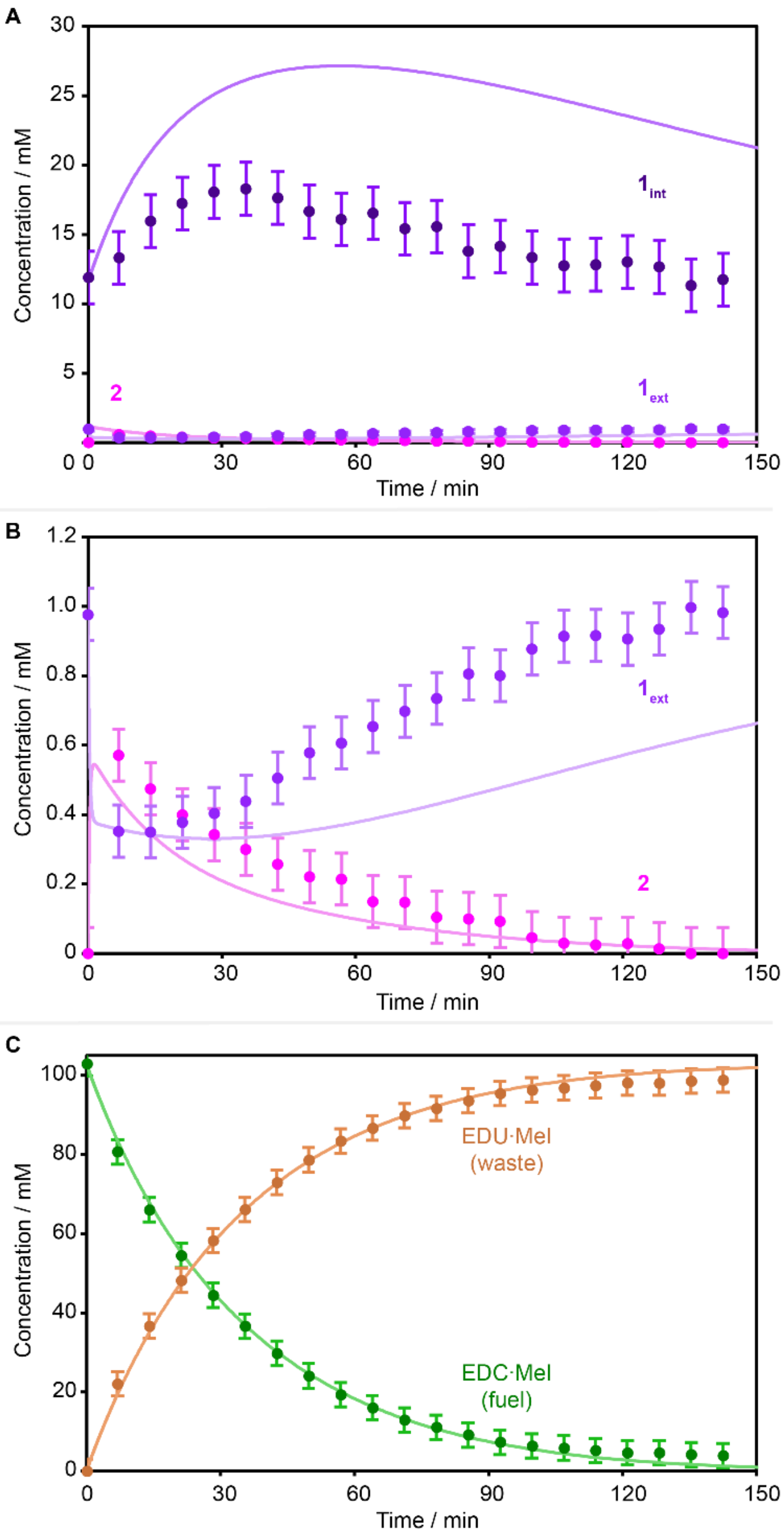


**Figure S25.** Kinetic plots showing the active concentrations of $\mathbf{1}_{int}$, $\mathbf{1}_{ext}$ and **2** (**A**, **B**) and EDC·MeI and EDU·MeI (**C**) over time. Solid lines show the overlaid kinetic model based on the independently measured rate constants. Error bars represent standard integral errors of 5% of the total concentration of $\mathbf{1}_{ext}$, $\mathbf{1}_{int}$ and **2** (**A**, **B**) and for 3% of the total concentration of EDC·MeI and EDU·MeI (**C**).

### S3.7.6 Full Kinetic Model Using Fitted Leakage Rate Constants

Due to the changing pH during the active transport experiment, the rate constants for the leakage of **1** across the membrane was allowed to be fitted in both directions. This provided an average rate constant for the membrane leakage across the whole active transport experiment. The rate of leakage is the most pH-sensitive rate constant (changing by an order of magnitude over 0.4 pH units, Section 3.7.4), hence was allowed to fit.

With all the other rate constants independently derived to prevent over-fitting the model to the data, the model shows good agreement and consistency with the experimental data.

The normalised root-mean-square error (See Section S2.4) was calculated over 105 data points and found to be 7% for this model, showing good overall agreement the experimental data.

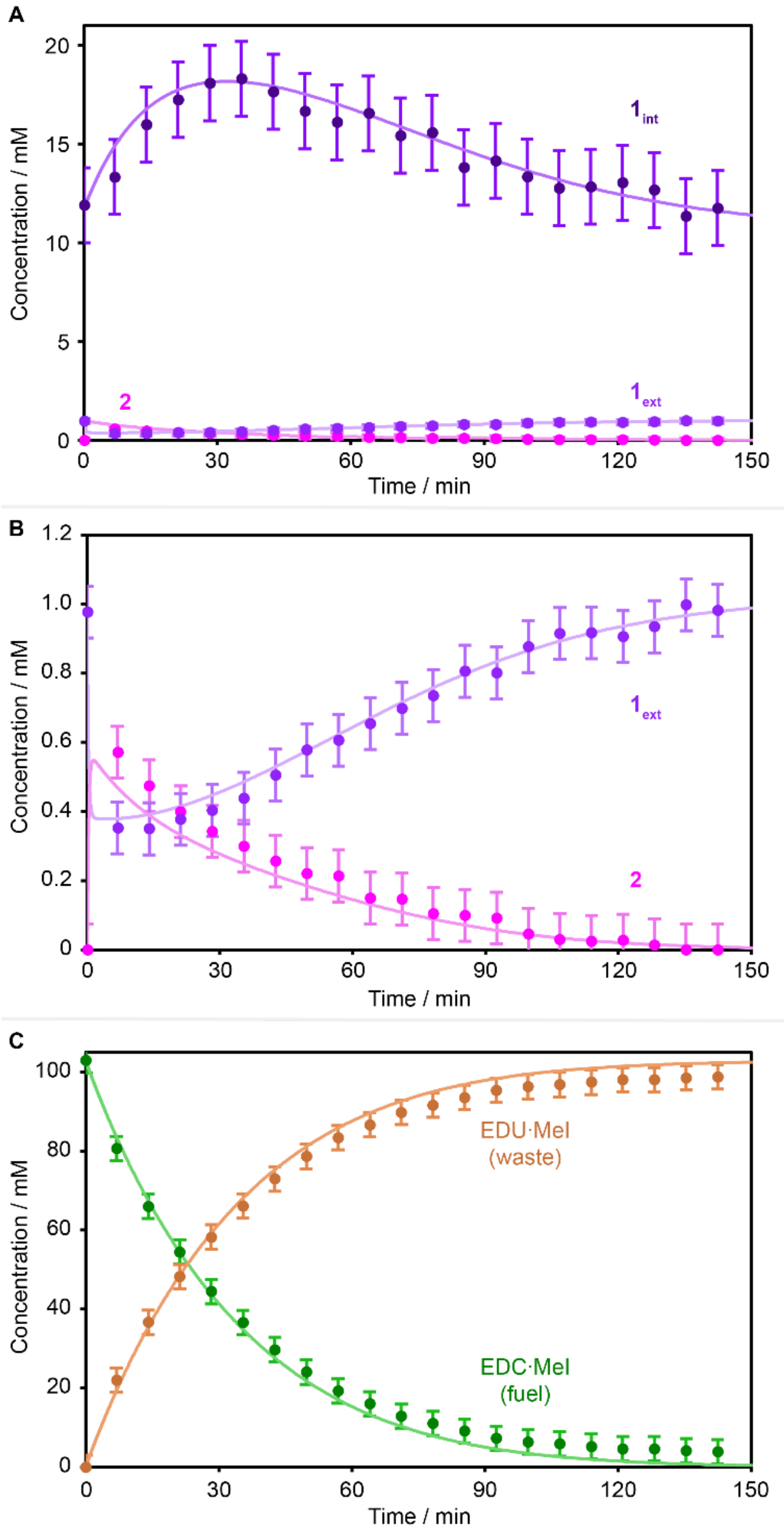


**Figure S26.** Kinetic plots showing the active concentrations of $\mathbf{1}_{int}$, $\mathbf{1}_{ext}$ and **2** (**A**, **B**) and EDC·MeI and EDU·MeI (**C**) over time. Solid lines show the overlaid kinetic model based on the independently measured rate constants with the rate of leakage allowed to fit. Error bars represent standard integral errors of 5% of the total concentration of $\mathbf{1}_{ext}$, $\mathbf{1}_{int}$ and **2** (**A**, **B**) and for 3% of the total concentration of EDC·MeI and EDU·MeI (**C**).

## Kinetic and Thermodynamic Analysis of Active Transport

## S3.8. Nonequilibrium Pumping Equation

The [$\mathbf{1_{int}}$]/[$\mathbf{1_{ext}}$] ratio is the primary measure of pumping performance, in particular when compared to the (pseudo) equilibrium ratio [$\mathbf{1_{int}}$]$_{eq}$/[$\mathbf{1_{ext}}$]$_{eq}$ = 12.1 achieved with passive transport (not equal to 1 due to the pH gradient). However, in our experiments, [$\mathbf{1_{int}}$]/[$\mathbf{1_{ext}}$] changes in time, as the pumping mechanism relies on EDC·MeI availability. An interesting question to evaluate pumping performance independently of time is therefore what would the [$\mathbf{1_{int}}$]/[$\mathbf{1_{ext}}$] ratio be if [EDC·MeI] were chemostatted at the initial condition and the system were given time to reach the corresponding steady state. In such a thought experiment, the resulting [$\mathbf{1_{int}}$]$_{ss}$/[$\mathbf{1_{ext}}$]$_{ss}$ is independent of observation time and EDC·MeI consumption, stemming from the intrinsic effectiveness of the pumping mechanism.

When [EDC·MeI] is chemostatted, the chemical reaction network describing transmembrane pumping is effectively unimolecular. Steady state concentrations in unimolecular systems can be computed via the Matrix Tree Theorem (King–Altman method).[S9–12] In particular, ratios of steady state concentrations take a general form known as the nonequilibrium pumping equality in the context of molecular machines.[S13–15] These tools can be applied to our systems, but since it involves compartments with different volumes, the nonequilibrium pumping equality should be derived starting from the unimolecular network describing changes in moles (m) instead of concentrations Once the rate constants are properly scaled with compartment volumes, the steady state ration reads:

$$\frac{[\mathbf{1}_{int}]}{[\mathbf{1}_{ext}]} = \frac{m_{\mathbf{1}_{int}}}{m_{\mathbf{1}_{ext}}} \times \frac{V_{ext}}{V_{int}} = K_{eq} + \lambda[\text{EDC·MeI}] \qquad \text{(Equation S43)}$$

Where m is the number of moles, V is the volume and λ is:

$$\lambda = \frac{k_{\text{anh form}}^{ext} \cdot k_{diff}^{ext\to int} \cdot k_{anh\ hyd}^{int}}{k_{leak}^{int\to ext} \times \left( k_{anh\ hyd}^{ext} \cdot k_{anh\ hyd}^{int} + k_{anh\ hyd}^{ext} \cdot k_{diff}^{int\to ext} / V_{int} + k_{anh\ hyd}^{int} \cdot k_{diff}^{ext\to int} / V_{ext} \right)} = 710.8\ M^{-1}$$

(Equation S44)

The above equation is particularly insightful. The linearity in [EDC·MeI] comes from the fact that EDC·MeI is present only in the external compartment, and implies that the pseudo equilibrium $\left(K_{eq} = \frac{k_{leak}^{ext\to int}}{k_{leak}^{int\to ext}}\right)$ is reached only when EDC·MeI is completely consumed. The "pumping factor" $\lambda[\text{EDC·MeI}]$ factorizes into contributions with distinct physical roles. The numerator $[\text{EDC·MeI}] \cdot k_{anh\ form}^{ext} \cdot k_{diff}^{ext\to int} \cdot k_{anh\ hyd}^{int}$ is the rate at which maleic acid is irreversibly deposited inside the vesicles. The denominator weights the relative contribution of the maleic acid leakage pathway from inside the vesicles and is dominated by the term $k_{anh\ hyd}^{int} \cdot k_{diff}^{ext\to int} / V_{ext}$ accounting for the rate at which maleic anhydride enters the vesicles and leaks as maleic acid. When the initial concentration $[\text{EDC}]_0$ is considered, the resulting steady state ratio is 85.1.

## S3.9. Pump Performance Calculations

Kinetic modelling (Section S3.8) allows the pump performance to be evaluated. The kinetic model was adapted to differentiate EDU·MeI waste formed by the catalyzed and background reactions.

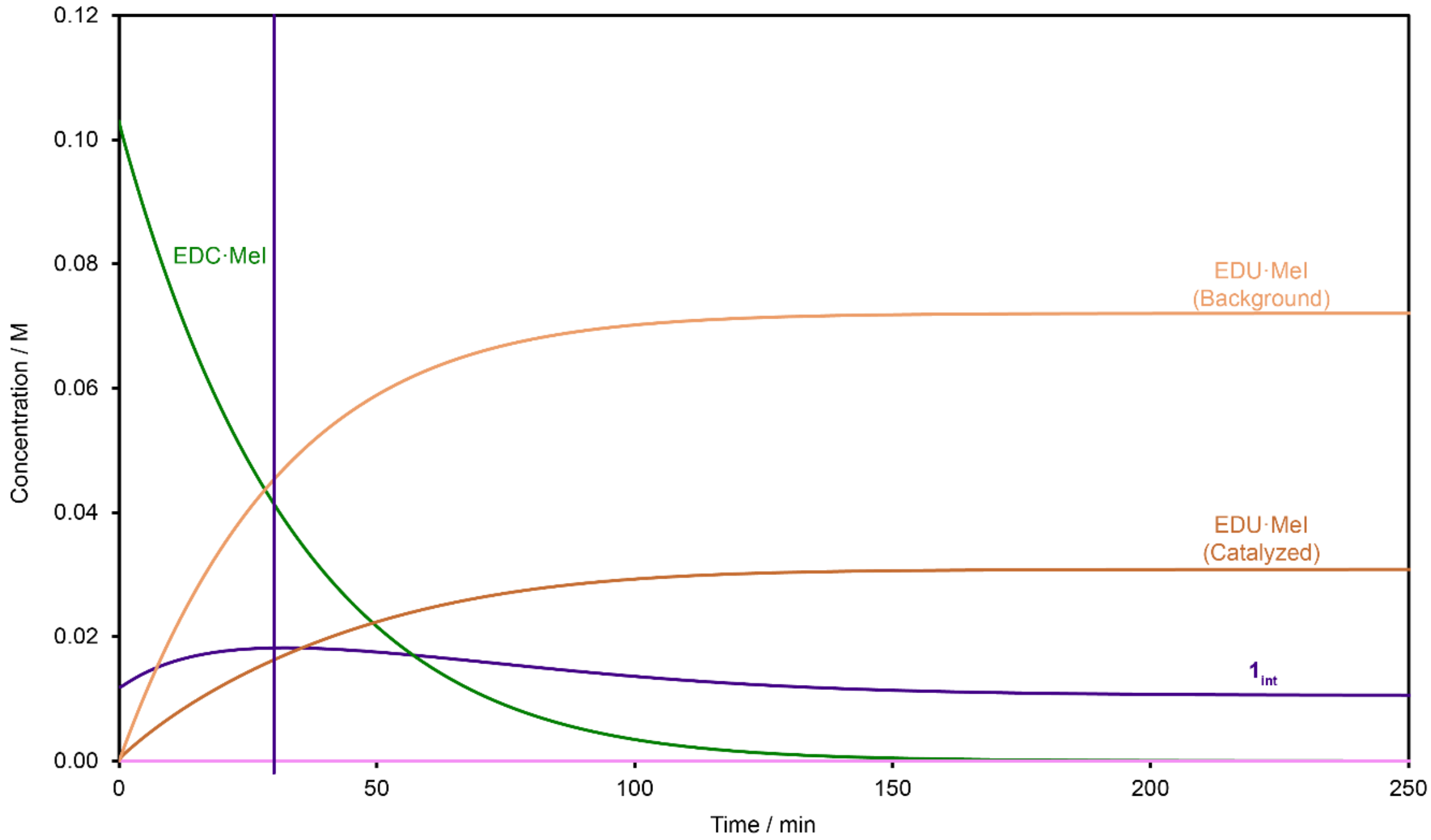


**Figure S27.** Kinetic model of the active transport with waste generated by the background (light orange) and catalyzed (dark orange) pathways separated out. The vertical line marks the maximum intravesicular [$\mathbf{1}_{int}$], at 32.4 mins.

The model shows that upon fueling with EDC·MeI (103 mM), the pump reaches a maximum internal concentration of maleic acid $\mathbf{1}_{int}$ after ~32.4 minutes ([$\mathbf{1}_{int}$]$_{34\ min}$ = 18.2 mM, vs [$\mathbf{1}_{int}$]$_{0\ min}$ = 11.9 mM). This corresponds to an increase in concentration of $\mathbf{1}_{int}$ of 6.3 mM, or approx. 153%. The total amount of EDC·MeI fuel used by this stage is 64.6 mM, with 17.1 mM consumed by the catalyzed pathway, and 47.4 mM used in the uncatalyzed hydration.

To calculate the performance, the absolute amounts of fuel, waste and cargo must be considered instead of concentration. The extravesicular volume = $6.74 \times 10^{-4}$ L, while the intravesicular volume = $2.52 \times 10^{-5}$ L (see Section S3.6). Thus, the amounts of species are as follows:

**Table S6.** Amounts of different species during active transport experiments.

| Species | Time = 0 min Concentration / mM | Time = 0 min Amount / μmol | Time = 32.4 min Concentration / mM | Time = 32.4 min Amount / μmol |
|---|---|---|---|---|
| $\mathbf{1}_{ext}$ | 0.977 | 0.659 | 0.468 | 0.316 |
| $\mathbf{1}_{int}$ | 11.9 | 0.299 | 18.2 | 0.457 |
| EDC·MeI | 103 | 68.5 | 38.4 | 25.9 |
| EDU·MeI (total) | 0 | 0 | 64.6 | 43.6 |
| EDU·MeI (catalyzed) | 0 | 0 | 17.1 | 11.6 |
| EDU·MeI (uncatalyzed) | 0 | 0 | 47.4 | 32.0 |

Based on these values, a series of key performance indications can be established based on the state the system reaches after 32.4 mins (maximum [$\mathbf{1}_{int}$]).

- The catalytic efficiency, that is the proportion of fuel that reacts via the catalyzed pathways $\left(\frac{\text{EDC·MeI (catalyzed)}_{32.4\text{ min}}}{\text{EDC·MeI (total)}_{32.4\text{ min}}}\right)$, is 27%.
- The pumping efficiency, that is the amount of cargo pumped as a proportion of the catalyzed fuel-to-waste reactions $\left(\frac{\mathbf{1}_{\text{int }32.4\text{ min}}-\mathbf{1}_{\text{int }0\text{ min}}}{\text{EDC·MeI (catalyzed)}_{32.4\text{ min}}}\right)$, is 1.4%. Note that this differs from the coupling efficiency that has previously been reported for molecular motors[S16] due to the significance of slip cycles for pumps.
- Thus, the overall efficiency (catalytic efficiency × pumping efficiency) = 0.4%.
- The average rate of pumping $\left(\frac{\mathbf{1}_{\text{int }32.4\text{ min}}-\mathbf{1}_{\text{int }0\text{ min}}}{32.4\text{ min}}\right)$, is 7.96 nmol min$^{-1}$.

## S3.10. Stored Energy Calculations

Creating a certain [**1**$_{int}$]/[**1**$_{ext}$] ratio starting from equilibrium has a certain minimal thermodynamic cost associated. This cost also corresponds to free energy stored in the system due to maleic acid disequilibrium, namely the maximum amount of free energy that can be in principle extracted from the spontaneous passive transport of a nonequilibrium maleic acid distribution towards equilibrium. In the ideal solution approximation, and considering maleic acid only, we can estimate the free energy store per unit volume, as it is usually reported in nonequilibrium systems chemistry works, as:[S4,S17]

$$G_{stored} = \frac{RT\left(V_{ext}\left([\mathbf{1}_{ext}]\cdot ln\frac{[\mathbf{1}_{ext}]}{[\mathbf{1}_{ext}]_{Eq}}+[\mathbf{1}_{ext}]_{Eq}-[\mathbf{1}_{ext}]\right)+V_{int}\left([\mathbf{1}_{int}]\cdot ln\frac{[\mathbf{1}_{int}]}{[\mathbf{1}_{int}]_{Eq}}+[\mathbf{1}_{int}]_{Eq}-[\mathbf{1}_{int}]\right)\right)}{(V_{ext}+V_{int})} \quad \text{(Equation S45)}$$

where R is the gas constant (8.314 J mol$^{-1}$ K$^{-1}$) and T the temperature (298.15 K). When considering the time at which the maximum [**1**$_{int}$]/[**1**$_{ext}$] ratio is reached, namely with [**1**$_{int}$]$_{32.4\ min}$ = 18.2 mM and [**1**$_{ext}$]$_{32.4\ min}$ = 0.468 mM, and the corresponding equilibrium concentrations, namely [**1**$_{int}$]$_{Eq}$ = 11.9 mM and [**1**$_{ext}$]$_{Eq}$ = 0.977 mM, the resulting free energy stored is 0.85 J L$^{-1}$.

If we consider that the maximum [**1**$_{int}$]/[**1**$_{ext}$] ratio is reached when 37.6 μmol of EDC·MeI has been consumed (assuming $\Delta G°_{EDC\cdot MeI\ hyd}$ = −80 kJ mol$^{-1}$ for EDC·MeI hydration), we get a thermodynamic efficiency[S4,S18] of about 0.02%.

# S4. Fluorescence Studies

## S4.1. General Vesicle Synthesis

Large unilamellar vesicles were prepared using the extrusion method as follows. Thin films of lipid were prepared in Eppendorf tubes by evaporating a chloroform solution of the lipid under a stream of nitrogen then under high vacuum for 6 hours. 1-palmitoyl-2-oleoyl-sn-3-phosphatidylcholine (POPC) or 1,2-dioleoyl-*sn*-glycero-3-phosphocholine (DOPC) were used either alone or in a 7:3 ratio with cholesterol. The lipid film was hydrated by vortexing with 1 mL of the prepared internal buffer solution then subjected to 5 freeze-thaw cycles using liquid nitrogen and a water bath (40°C). The vesicles were then extruded 19 times through a polycarbonate membrane (pore size 100 nm or 200 nm). Extravesicular components were removed by size exclusion chromatography on a Sephadex G-25 column as follows, the column was flushed with the prepared external buffer solution, the 1 mL vesicles solution was added to the column with 1.5 mL of external buffer. A further 3.5 mL of external buffer was added, and the purified vesicles were collected (3.5 mL). The concentration of the collected lipid stock is dependent on the initial lipid film concentration.

## S4.2. General HPTS Assay and Calibration

The kinetic fluorescence assays employ 8-hydroxypyrene-1,3,6-trisulfonic acid trisodium salt (HPTS) as an internal pH probe to monitor vesicle lumen acidification induced by maleic anhydride **2** hydrolysis into maleic acid **1** once chemically driven across the bilayer. The time-dependent change in fluorescence intensity ($\lambda_{em}$ = 510 nm) was monitored simultaneously at $\lambda_{ex}$ = 405 nm (acidic form of HPTS) and $\lambda_{ex}$ = 460 nm (basic form) which allowed for ratiometric analysis (Equation S46).

$$R = \frac{\lambda_{ex}\,460}{\lambda_{ex}\,405} \qquad \text{(Equation S46)}$$

The data was then normalized by dividing each fluorescence ratio at time t ($R_t$) by the initial fluorescence ratio at *t* = 0 ($R_0$) immediately before fuel addition, yielding $R/R_0$. All experiments were performed in triplicate, normalized individually, and then averaged to produce the final plot with error bars as the standard deviation of the sample.

HPTS calibration was carried out according to previously reported procedures.[S6] Since the change in fluorescence intensities of the basic form ($\lambda_{ex}$ = 465 nm) and protonated form ($\lambda_{ex}$ = 405 nm) of HPTS are proportional to the pH of the aqueous solution, the Henderson-Hasselbach equation can be used to calibrate the fluorescence changes intensities of HPTS in relation to pH following calibration.

POPC LUVs were made according to the general vesicle synthesis procedure described above with an internal solution of 200 mM NaCl, 100 mM MOPS and 3 mM HPTS, ±50 mM **1**, buffered to pH 6.5 and an external solution of 200 mM NaCl, ±50 mM **1** and 100 mM MOPS again buffered to pH 6.5. Calibration was performed with and without the presence of diacid **1**. The POPC LUVs (100 μL, 0.1 mM final concentration, 100 nm) were suspended in the external buffer solution (2400 μL) and the pH was adjusted using NaOH or HCl (measured to 2 d.p. accuracy using the pH meter). The transmembrane pH gradient was equilibrated using $H^+/Cl^-$ symporter prodigiosin (8 μL, 10 nM final concentration, 0.01 mol% with respect to lipid) and the HPTS emission ($\lambda_{em}$ = 510 nm) was recorded ($\lambda_{ex}$ = 405 and 465 nm) until stable, ca. 5 mins. This was repeated over a range of pH values.

The logarithm of the ratiometric intensity, R (460/405) was plotted as a function of pH and fitting to a linear regression to obtain the apparent p*Ka'* of HPTS under the experimental conditions (Equations S47–49). This value was subsequently used to convert ratiometric fluorescence data from transport experiments to apparent intravesicular pH values.

$$pH \;=\; pK_a + \log\frac{[\mathrm{A^-}]}{[\mathrm{HA}]} \qquad \text{(Equation S47)}$$

$$pH \;=\; pK_a{}' + \log\left(\frac{\lambda_{ex}\,460}{\lambda_{ex}\,405}\right) \qquad \text{(Equation S48)}$$

$$pK_a{}' = -\frac{intercept}{slope} \qquad \text{(Equation S49)}$$

To further validate the fluorescence response and calibration, the derivatives $K_b'$ and $K_a'$ of HPTS were obtained by plotting the ratiometric intensity R (460/F40) as a function of $[OH^-]$, and the inverse ratiometric intensity R (405/460) as a function of $[H^+]$ concentration (Equations S50–52). The $[OH^-]$, and $[H^+]$ concentrations were calculated from the measured pH values using Equation S53 and S54.

$$K_b{}' = \frac{[\mathrm{OH^-}][\mathrm{HA}]}{[\mathrm{A^-}]} \quad \text{i.e.} \quad \frac{\lambda_{ex}\,460}{\lambda_{ex}\,405} = \frac{[\mathrm{OH^-}]}{K_b{}'} \qquad \text{(Equation S50)}$$

$$K_a' = \frac{[\mathrm{H}^+][\mathrm{A}^-]}{[\mathrm{HA}]} \quad \text{i.e.} \quad \frac{\lambda_{ex}\,405}{\lambda_{ex}\,460} = \frac{[\mathrm{H}^+]}{K_a'} \qquad \text{(Equation S51)}$$

$$K_b' \; or \; K_a' = \frac{(1 - intercept)}{slope} \qquad \text{(Equation S52)}$$

$$[\mathrm{OH}^-] = 10^{-(14-\mathrm{pH})} \qquad \text{(Equation S53)}$$

$$[\mathrm{H}^+] = 10^{-\mathrm{pH}} \qquad \text{(Equation S54)}$$

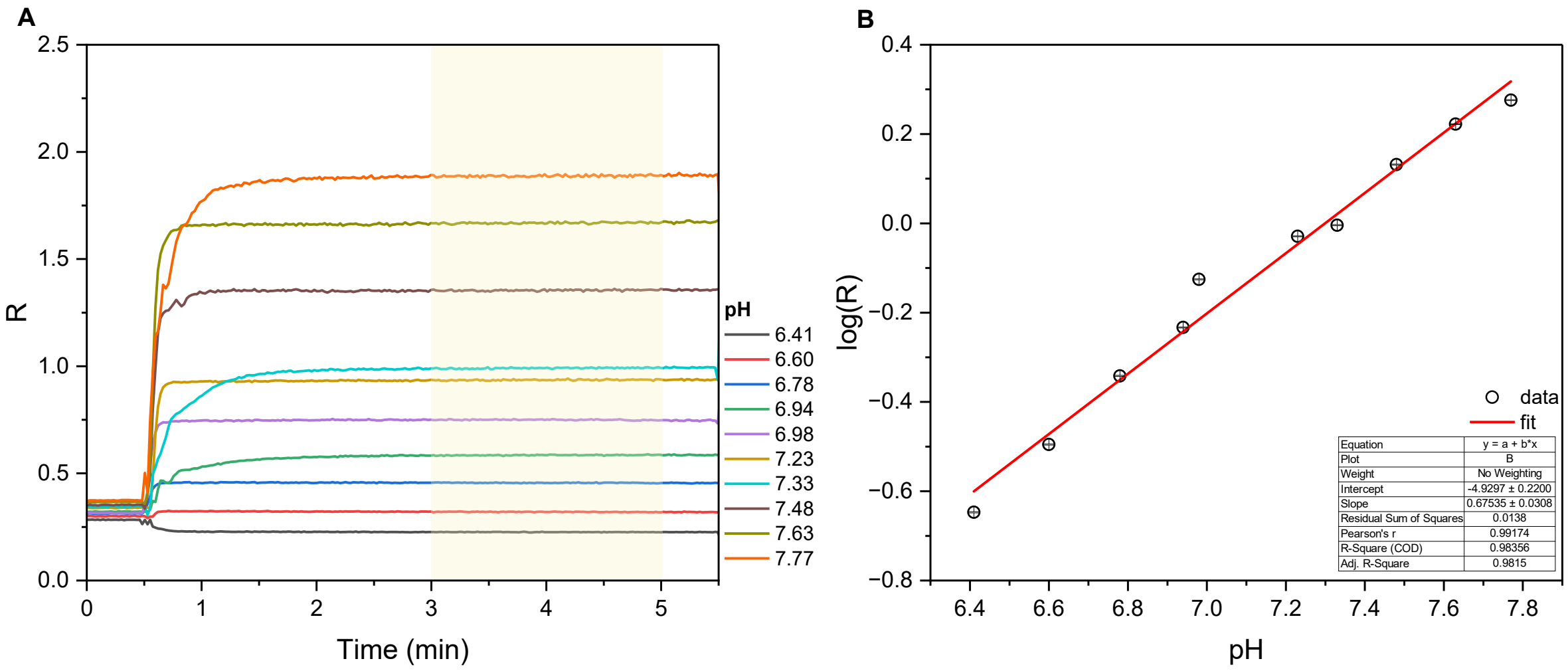


**Figure S28**. HPTS pH calibration. **A** Change in the relative fluorescence intensity (R) of HPTS over time ($\lambda_{em}$ = 510 nm; $\lambda_{ex1}$ = 405 nm, $\lambda_{ex2}$ = 460 nm) after adjusting the external pH. Prodigiosin (0.01 mol%, 10 nM final concentration) added at *t* = 30 s. POPC LUVs (0.1 mM, 100 nm); Internal: 3 mM HPTS, 200 mM NaCl, 100 mM MOPS, 50 mM **1**, External: 200 mM NaCl, 100 mM MOPS, 50 mM **1**. Measurements between 3 – 5 min (yellow) used for later standard deviation. **B** Plot of the logarithm of ratiometric intensity R (460/405) as a function of pH and fitted to a linear regression to obtain the pKa' of HPTS, here, 7.3 (50 mM **1** present in internal and external vesicle buffer). Error bars are SD from measurements between 3 – 5 min.

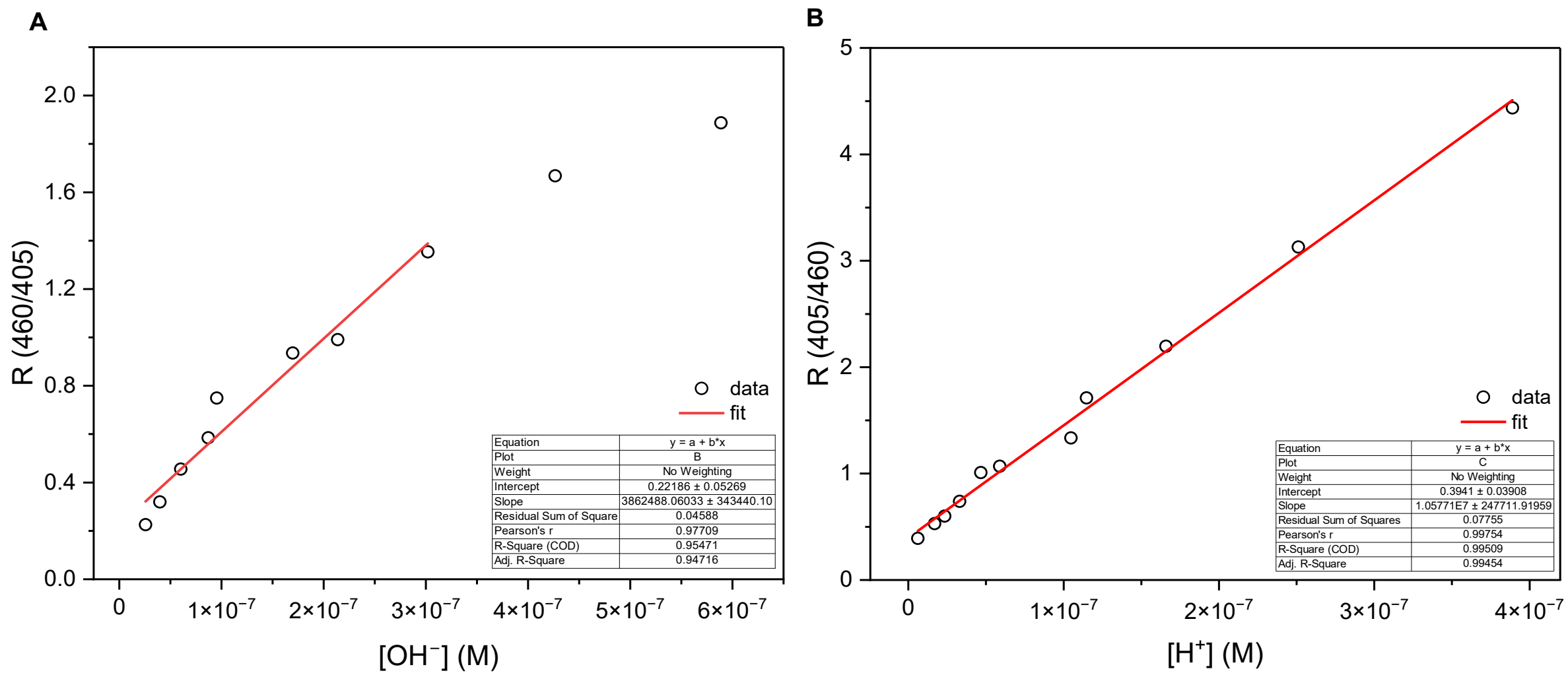


**Figure S29**. **A** Plot of the ratiometric intensity R (460/405) as a function of [OH⁻], fitted to a linear regression to obtain the $K_b$' of HPTS, here 2.01 x $10^{-7}$ M. **B** Plot of the inverse ratiometric intensity R (405/460) as a function of [H⁺], fitted to a linear regression to obtain the $K_a$' of HPTS, here 5.73 x $10^{-8}$ M. POPC LUVs (0.1 mM, 100 nm); Internal: 3 mM HPTS, 200 mM NaCl, 100 mM MOPS, 50 mM **1**, External: 200 mM NaCl, 100 mM MOPS, 50 mM **1**.

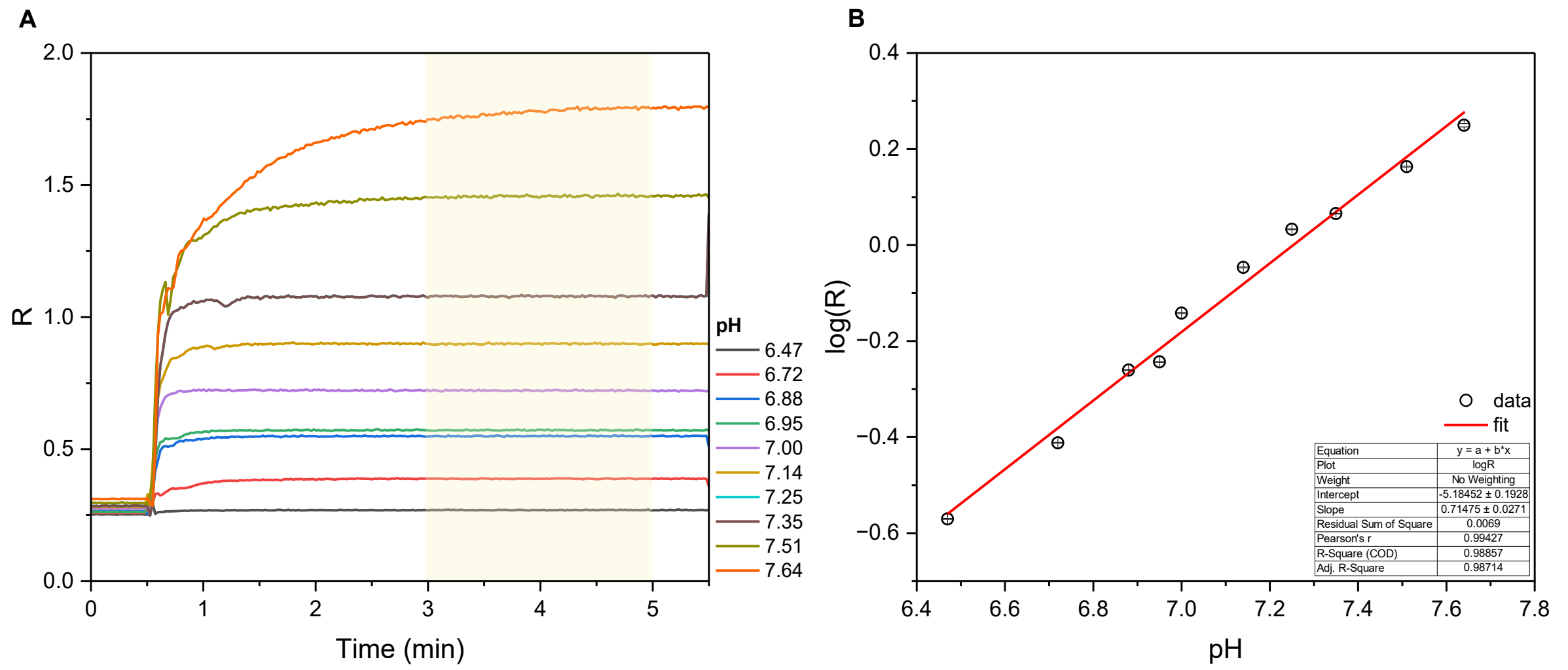


**Figure S30**. HPTS pH calibration. **A** Change in the relative fluorescence intensity (R) of HPTS over time ($\lambda_{em}$ = 510 nm; $\lambda_{ex1}$ = 405 nm, $\lambda_{ex2}$ = 460 nm) after adjusting the external pH. Prodigiosin (0.01 mol%, 10 nM final concentration) added at *t* = 30 s. POPC LUVs (0.1 mM, 100 nm); Internal: 3 mM HPTS, 200 mM NaCl, 100 mM MOPS, External: 200 mM NaCl, 100 mM MOPS. Measurements between 3 – 5 min (yellow) used for standard deviation. **B** Plot of the logarithm of ratiometric intensity R (460/405) as a function of pH and fitted to a linear regression to obtain the pKa' of HPTS, here 7.25 (diacid **1** not present). Error bars are SD from measurements between 3 – 5 min.

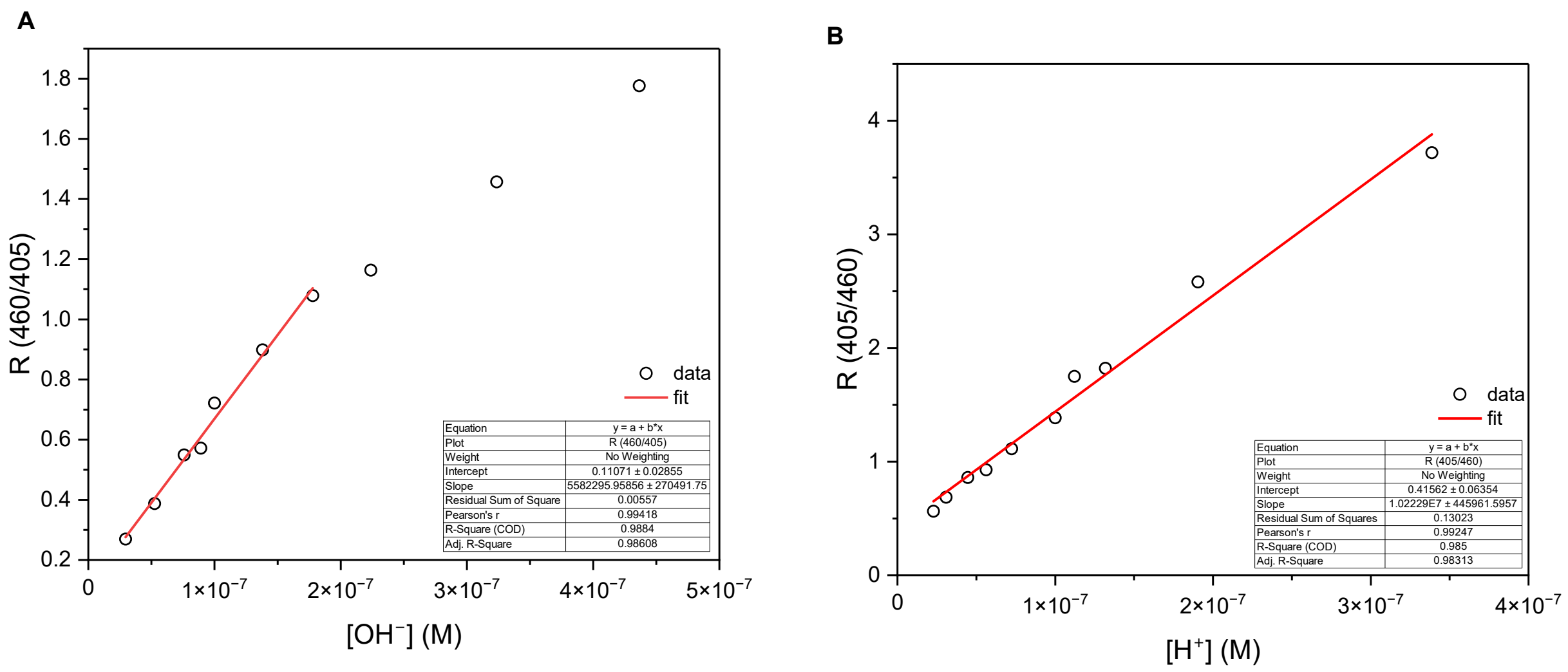


**Figure S31**. **A** Plot of the ratiometric intensity R (460/405) as a function of [OH⁻], fitted to a linear regression to obtain the $K_b$' of HPTS, here 1.48 x $10^{-7}$ M. **B** Plot of the inverse ratiometric intensity R (405/460) as a function of [H⁺], fitted to a linear regression to obtain the $K_a$' of HPTS, here 5.84 x $10^{-8}$ M. POPC LUVs (0.1 mM, 100 nm); Internal: 3 mM HPTS, 200 mM NaCl, 100 mM MOPS, External: 200 mM NaCl, 100 mM MOPS.

Overall, there is consistency between calculated pKa values with and without 50 mM diacid **1**. When plotting the ratiometric intensity R (460/405) as a function of [OH⁻] a linear dependence was observed over the working pH range. Deviations from linearity were observed at higher pH, consistent with reported inner filter and optical effects.[S6] Accordingly, only the linear working range was used for calibration and subsequent data analysis. When plotting the inverse ratiometric intensity R (405/460) as a function of [H⁺] concentration, the relationship was approximately linear over the same working range, supporting the validity of the calibration.

## S4.3. HPTS Active Transport Assays (EDC·MeI fuel)

### S4.3.1 $pH_{in} = pH_{out}$

Here, thin lipid films of POPC (266 µL, 25 mg/mL in chloroform) were used to prepare POPC LUVs (3.5 mL, 2.5 mM, 100 nm) with an internal solution of 200 mM NaCl, 100 mM MOPS and 3 mM HPTS, ±50 mM **1**, buffered to pH 6.5 and an external solution of 200 mM NaCl, ±50 mM **1** and 100 mM MOPS again buffered to pH 6.5, using the general vesicle synthesis procedure described above.

In the transport experiments, the POPC LUVs (100 µL) were suspended in the external buffer solution (2350 µL) then EDC·MeI fuel (50 µL, 10 mM final concentration) was added at $t$ = 1 min to initiate the experiment; giving a standard volume (2.5 mL) and a final lipid concentration of 0.1 mM. The change in fluorescence was monitored over time then Triton X-100 detergent (50 µL, 7:1 (*v*/*v*) $H_2O$/DMSO) was added to lyse the vesicles and terminate the assay.

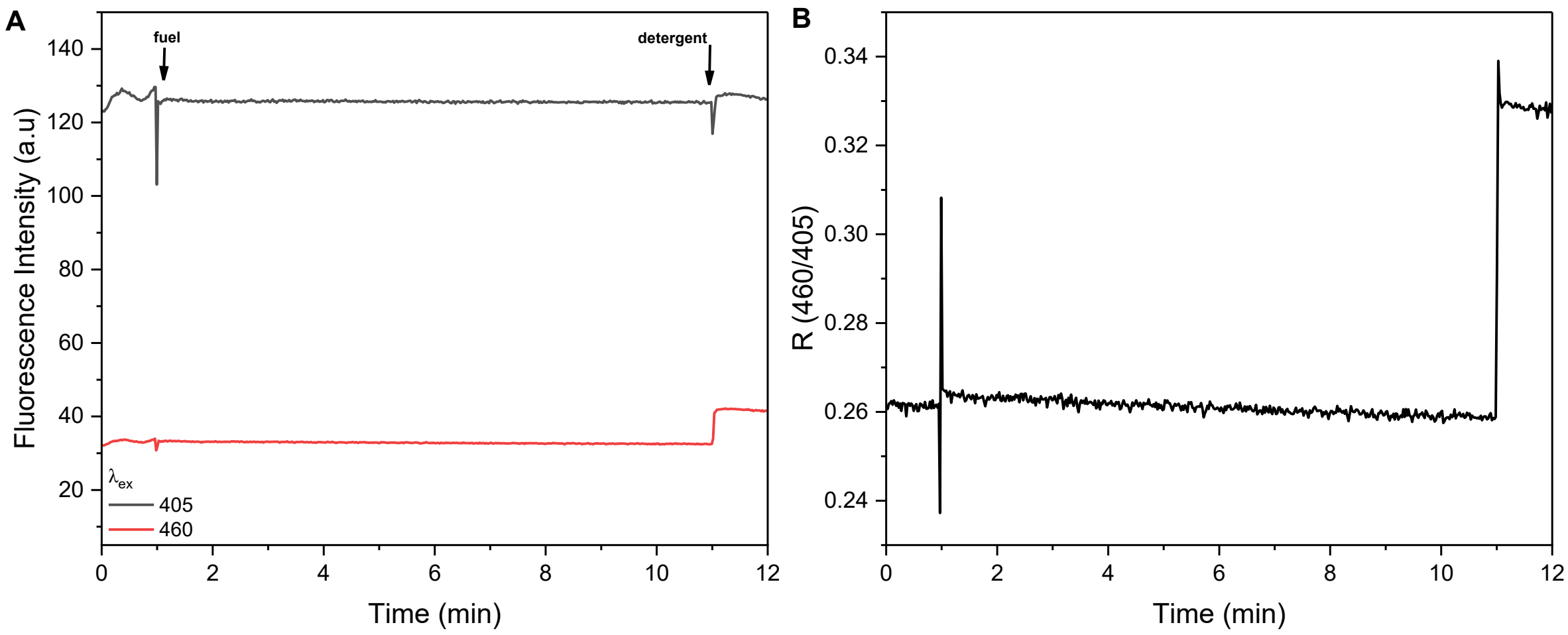


**Figure S32**. **A** Raw data showing the change in fluorescence over time ($\lambda_{em}$ = 510 nm; $\lambda_{ex1}$ = 405 nm, $\lambda_{ex2}$ = 460 nm) upon addition of EDC·MeI (50 µL, 10 mM final concentration) to POPC LUVs (0.1 mM, 100 nm). Internal: 200 mM NaCl, 100 mM MOPS and 3 mM HPTS, pH 6.5. External: 200 mM NaCl, and 100 mM MOPS pH 6.5. Vesicle lysis by Triton X-100 (50 µL). **B** Plot of the relative fluorescence intensity (R) over time.

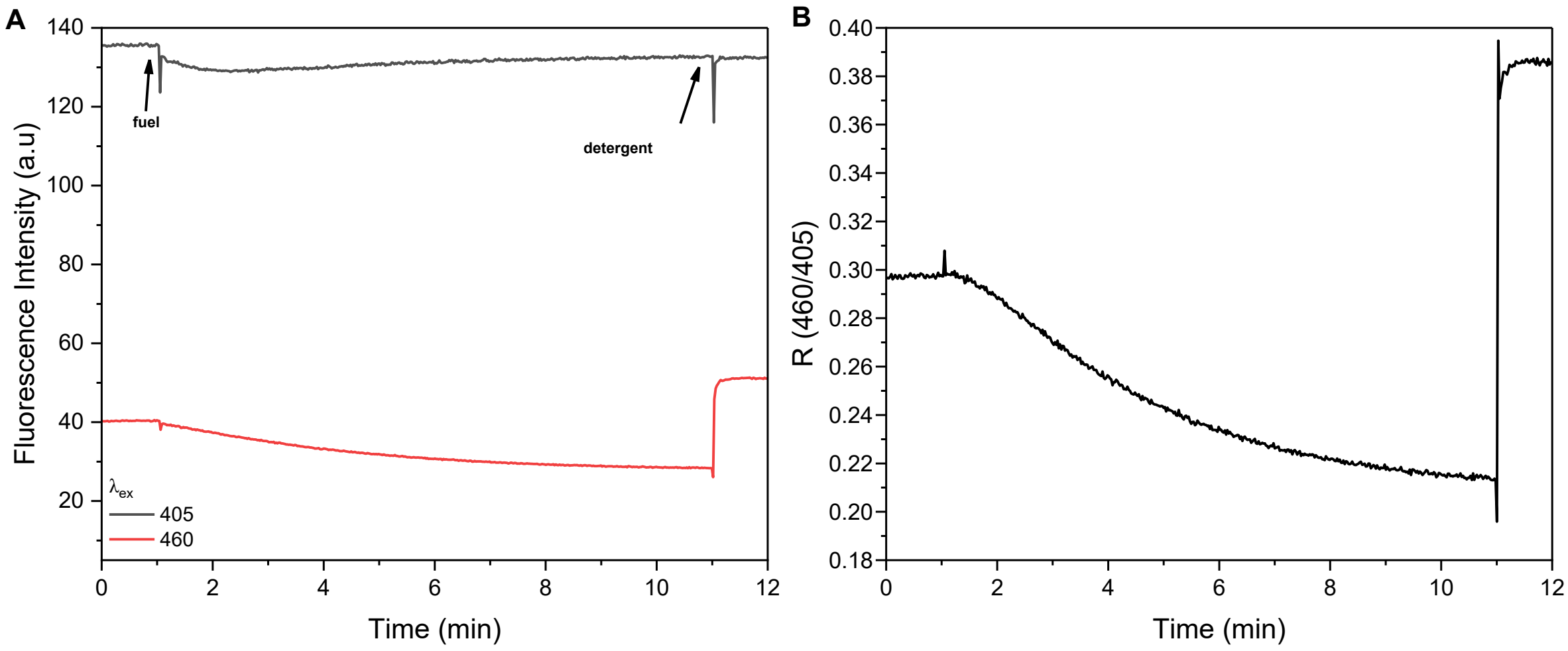


**Figure S33**. **A** Raw data showing the change in fluorescence over time ($\lambda_{em}$ = 510 nm; $\lambda_{ex1}$ = 405 nm, $\lambda_{ex2}$ = 460 nm) upon addition of EDC·MeI (50 µL, 10 mM final concentration) to POPC LUVs (0.1 mM, 100 nm) Internal: 200 mM NaCl, 50 mM **1**, 100 mM MOPS and 3 mM HPTS, pH 6.5. External: 200 mM NaCl, 50 mM **1** and 100 mM MOPS pH 6.5. Vesicle lysis by Triton X-100 (50 µL). **B** Plot of the relative fluorescence intensity (R) over time.

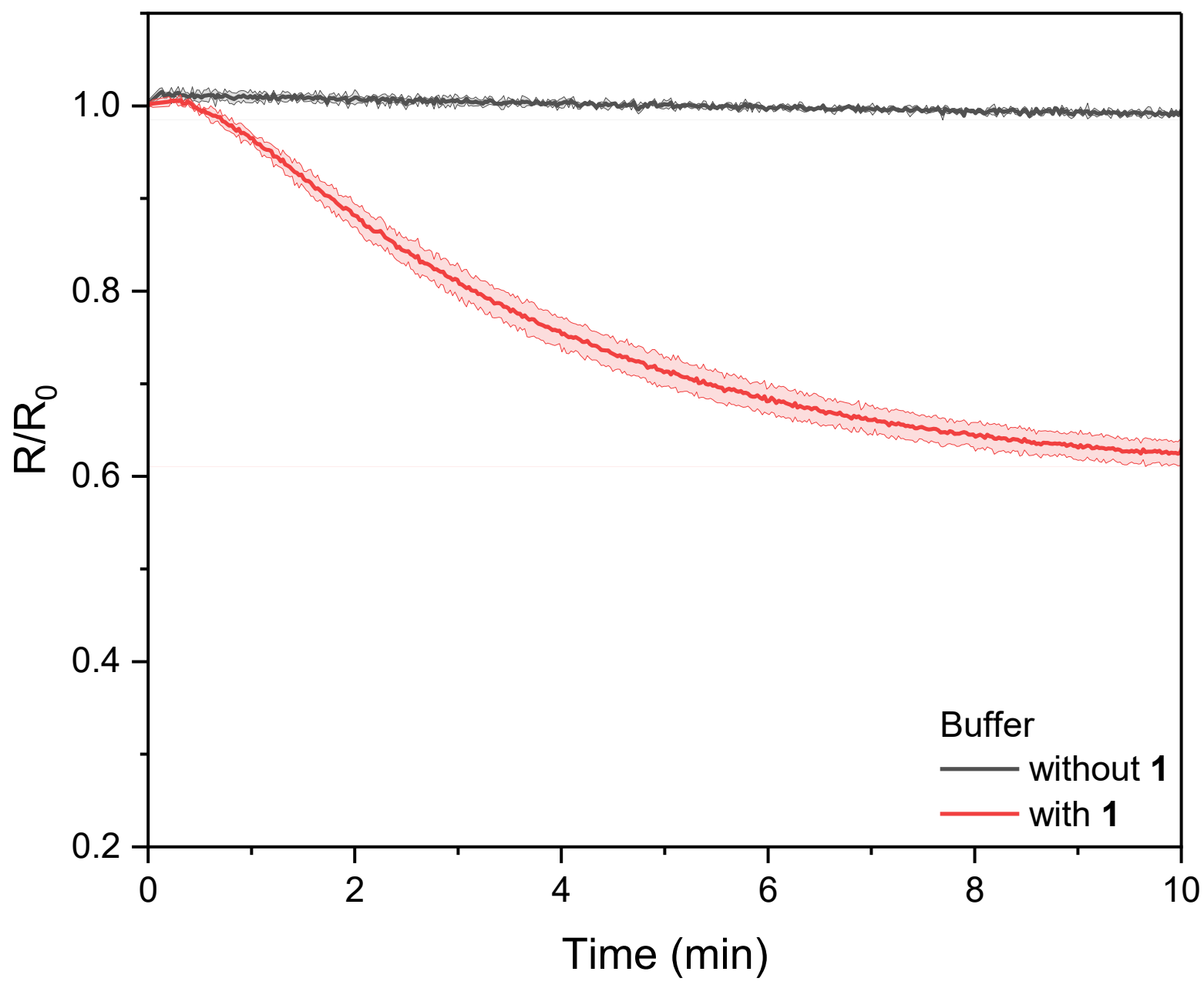


**Figure S34**. Active Transport of **1** across liposomes (0.1 mM vesicles, POPC). Internal: 200 mM NaCl, ±50 mM **1**, 100 mM MOPS and 3 mM HPTS, pH 6.5. External: 200 mM NaCl, ± 50 mM **1** and 100 mM MOPS pH 6.5. EDC·MeI (10 mM) was added at *t* = 0 and the change in fluorescence was followed for 10 mins.

To confirm the change in fluorescence was a result from active transport and not caused by EDU·MeI waste facilitating passive ion transport, EDC·MeI fuel was allowed to hydrolyse to EDU·MeI for 48 hrs and used in place of EDC·MeI fuel. No change in fluorescence was observed (Figure S35), indicating that the urea waste does not mediate active transport or ion transport and is thus not involved in lumen acidification. The reduced emission in the acidic form of HPTS at *t* = 0 (directly following addition) is observed with both EDC and EDU addition, and attempts to identify a possible ester formed upon reaction of HPTS and maleic anhydride

were unsuccessful. Repeating the assay with a lower intravesicular HPTS concentration afforded similar results.

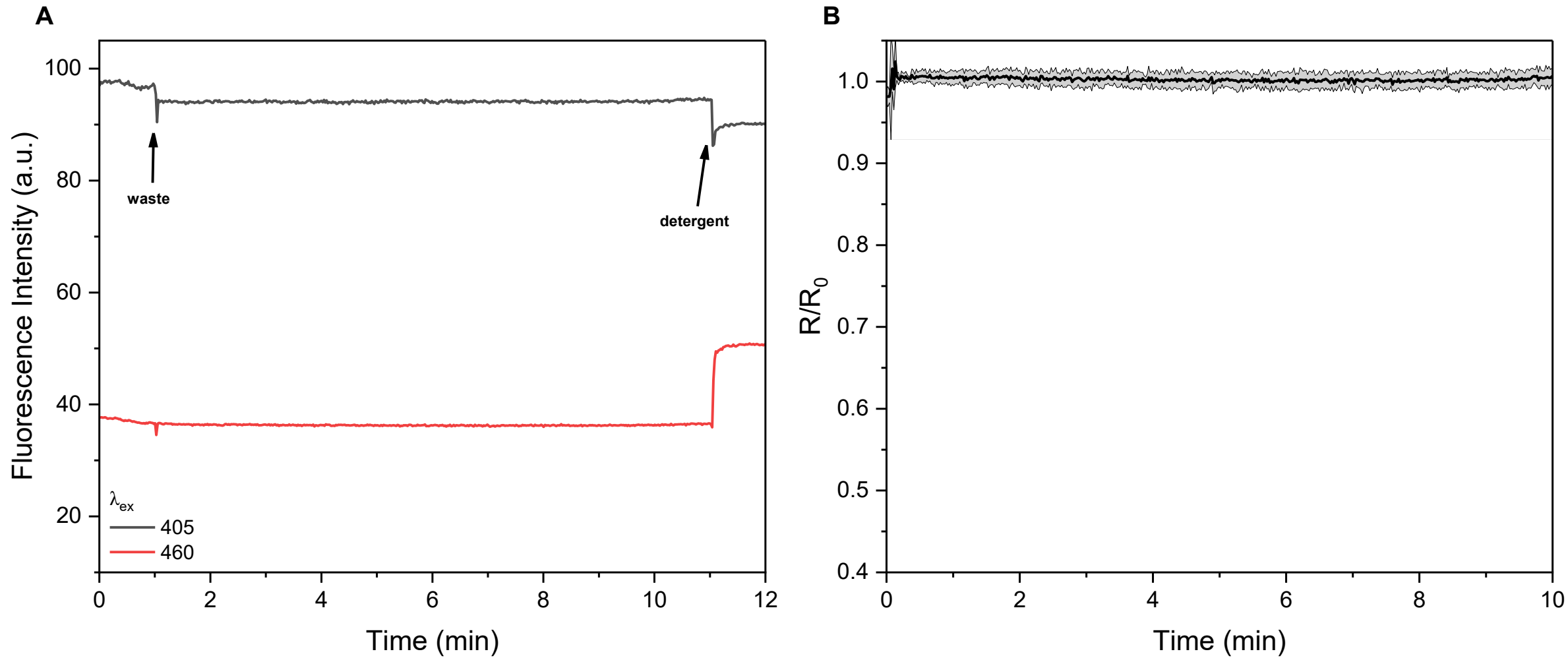


**Figure S35**. **A** Raw data showing the change in fluorescence over time ($\lambda_{em}$ = 510 nm; $\lambda_{ex1}$ = 405 nm, $\lambda_{ex2}$ = 460 nm) upon addition of EDU·MeI waste (50 µL, 10 mM final concentration) to POPC LUVs (0.1 mM, 100 nm). Internal: 200 mM NaCl, 50 mM **1**, 100 mM MOPS and 3 mM HPTS, pH 6.5. External: 200 mM NaCl, 50 mM **1** and 100 mM MOPS pH 6.5. Vesicle lysis by Triton X-100 (50 µL). **B** Plot of the normalised relative fluorescence intensity over time, SD from 3 repeats.

### S4.3.2 Different Concentrations of Fuel

Here, we investigated the role of changing the concentration of fuel used on the gradient generated.

Thin lipid films of POPC (266 µL, 25 mg/mL in chloroform) were used to prepare POPC LUVs (3.5 mL, 2.5 mM, 100 nm) with an internal solution of 200 mM NaCl, 100 mM MOPS and 3 mM HPTS, ± 50 mM **1**, buffered to pH 6.5 and an external solution of 200 mM NaCl, ± 50 mM **1** and 100 mM MOPS again buffered to pH 6.5, using the general vesicle synthesis procedure described above.

In the transport experiments, the POPC LUVs (100 µL) were suspended in the external buffer solution (2350 µL) then EDC·MeI fuel (50 µL, 0–20 mM final concentration) was added at $t$ = 0; giving a standard volume (2.5 mL) and a final lipid concentration of 0.1 mM. The change in fluorescence was monitored over time and Triton X-100 detergent (50 µL, 7:1 ($v/v$) $H_2O$-DMSO) was used to lyse the vesicles and terminate the assay.

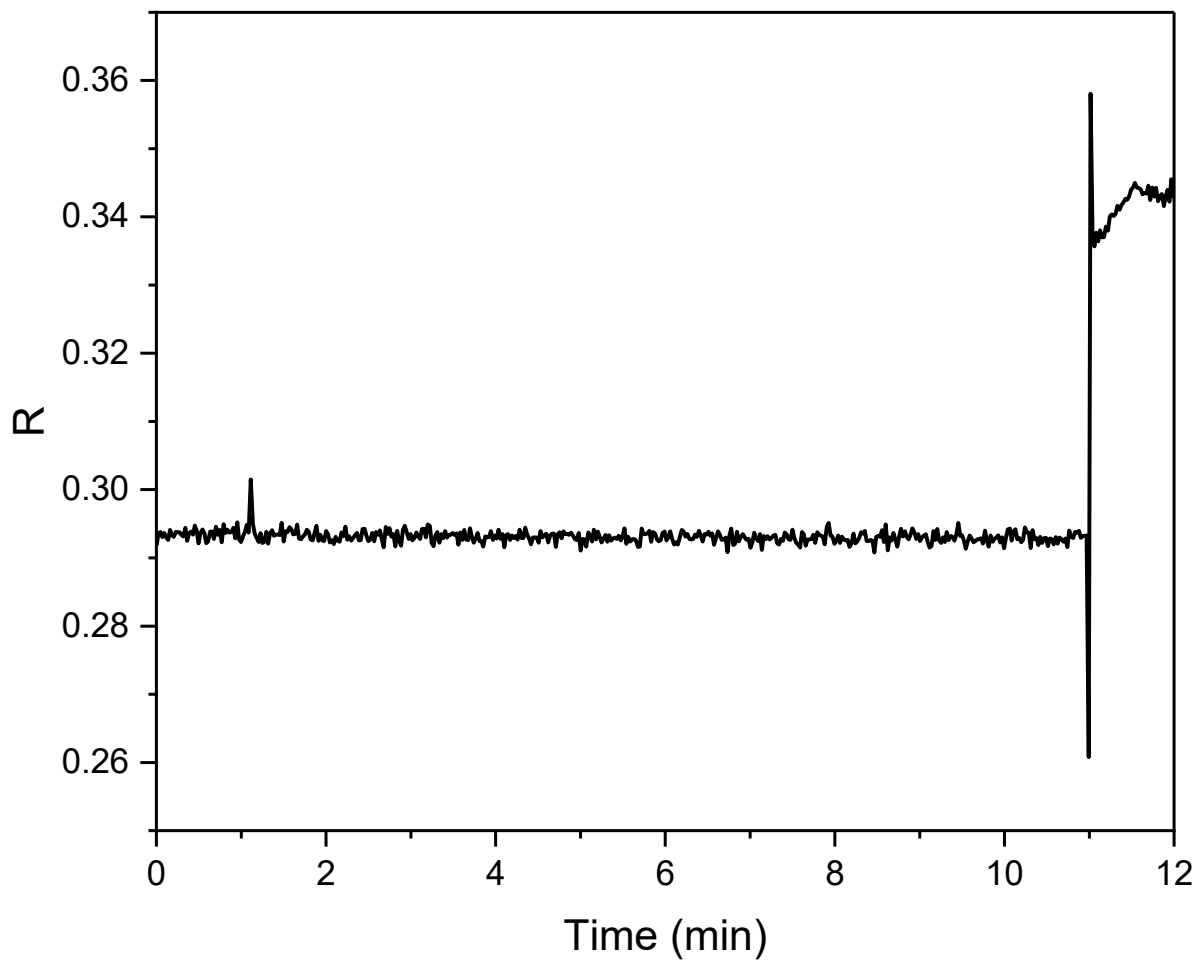


**Figure S36**. Change in the relative fluorescence intensity (R) of HPTS over time ($\lambda_{em}$ = 510 nm; $\lambda_{ex1}$ = 405 nm, $\lambda_{ex2}$ = 460 nm) upon addition of buffer only (50 µL, 200 mM NaCl, 100 mM MOPS pH 6.5) to POPC LUVs (0.1 mM, 100 nm). Vesicle lysis by Triton X-100. Experiments repeated in triplicate.

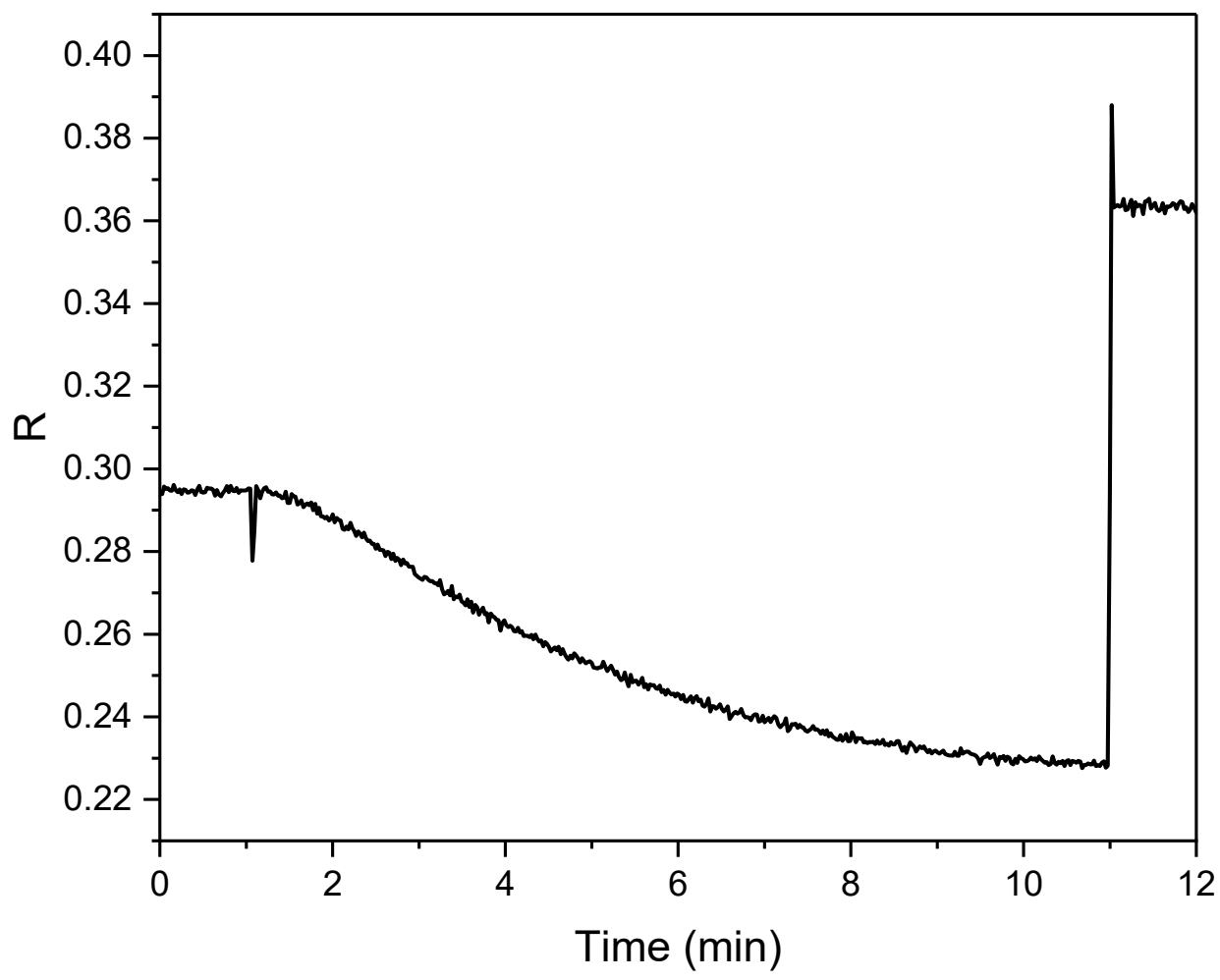


**Figure S37**. Change in the relative fluorescence intensity (R) of HPTS over time ($\lambda_{em}$ = 510 nm; $\lambda_{ex1}$ = 405 nm, $\lambda_{ex2}$ = 460 nm) upon addition of EDC·MeI fuel (50 µL, 5 mM final concentration) to POPC LUVs (0.1 mM, 100 nm). Vesicle lysis by Triton X-100. Experiments repeated in triplicate.

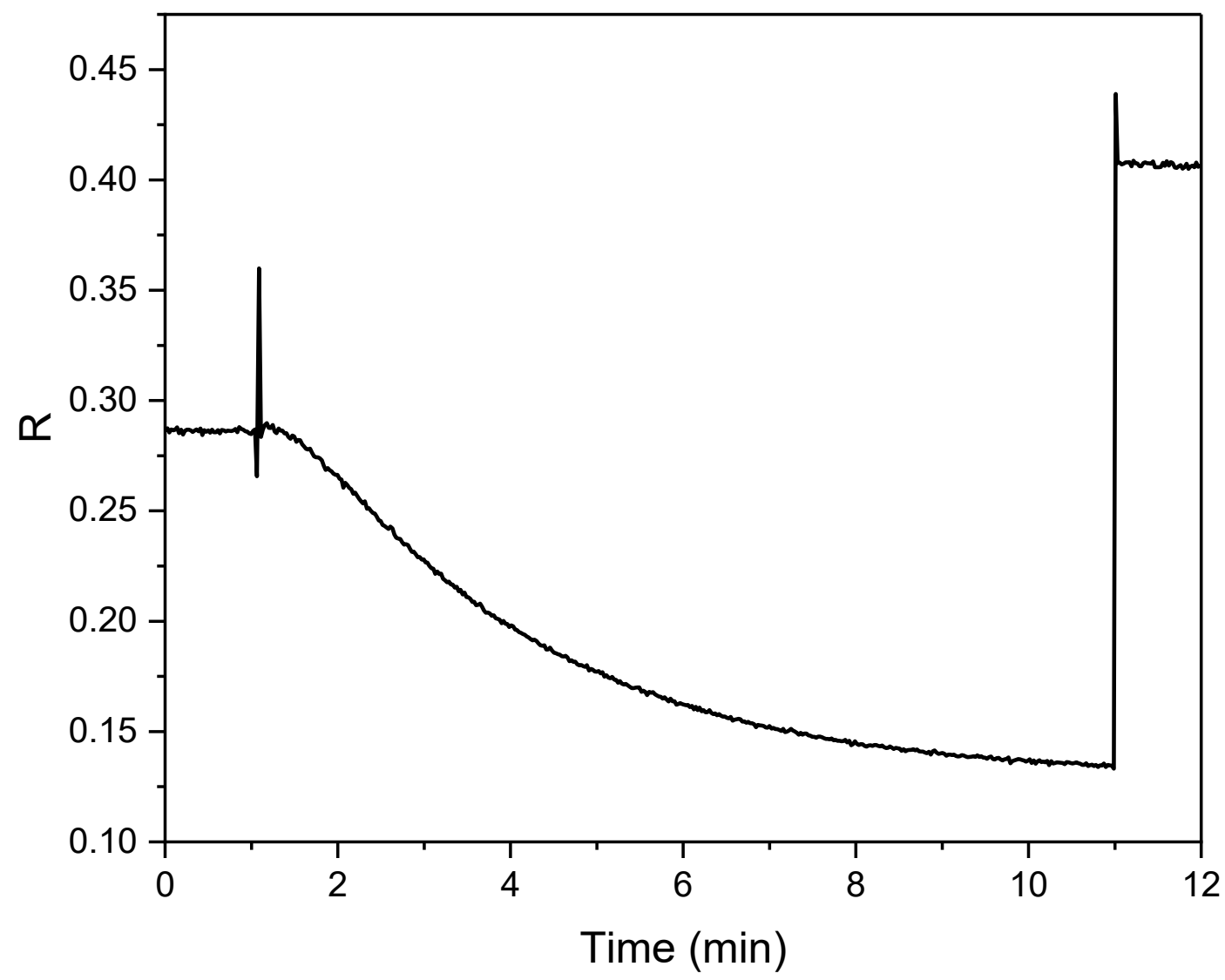


**Figure S38**. Change in the relative fluorescence intensity (R) of HPTS over time ($\lambda_{em}$ = 510 nm; $\lambda_{ex1}$ = 405 nm, $\lambda_{ex2}$ = 460 nm) upon addition of EDC·MeI fuel (50 µL, 20 mM final concentration) to POPC LUVs (0.1 mM, 100 nm). Vesicle lysis by Triton X-100. Experiments repeated in triplicate.

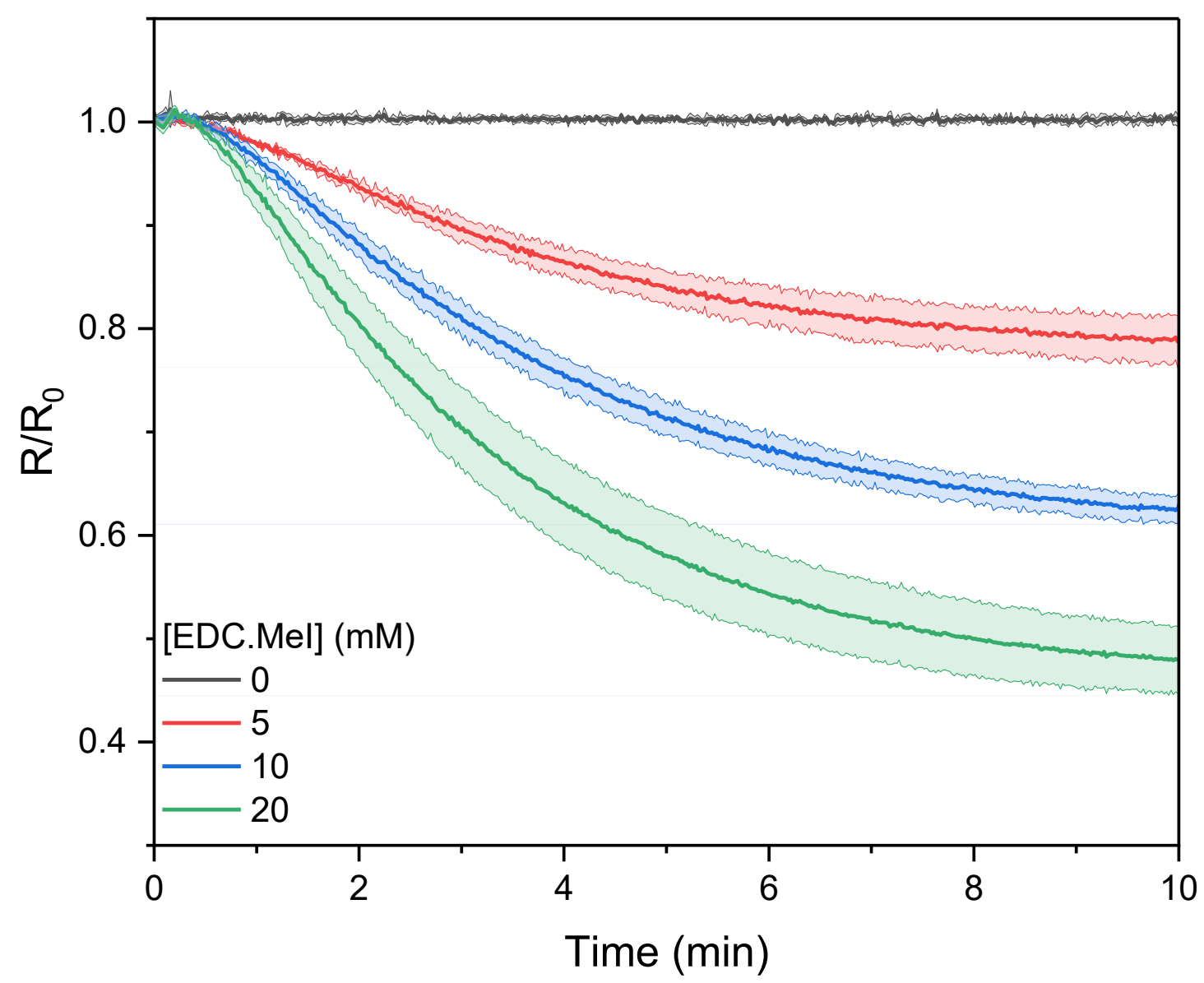


**Figure S39**. Active Transport of **1** across POPC LUVs (0.1 mM, 100 nm) upon addition of EDC·MeI fuel (50 µL, 0-20 mM final concentration) at *t* = 0. Internal: 200 mM NaCl, 50 mM **1**, 100 mM MOPS and 3 mM HPTS, pH 6.5. External: 200 mM NaCl, 50 mM **1** and 100 mM MOPS pH 6.5.

### S4.3.3 Dynamic Light Scattering (DLS)

A thin lipid film of POPC (266 µL, 25 mg/mL in chloroform) were used to prepare POPC LUVs (3.5 mL, 2.5 mM, 100 nm) with an internal solution of 200 mM NaCl, 100 mM MOPS and 3 mM HPTS, ± 50 mM **1**, buffered to pH 6.5 and an external solution of 200 mM NaCl, ± 50 mM **1** and 100 mM MOPS again buffered to pH 6.5, using the general vesicle synthesis procedure described above.

For the active transport experiment, the POPC LUVs (100 µL, 100 nm, 0.1 mM final concentration) were suspended in external buffer solution (2350 µL) prior to addition of EDC·MeI fuel (50 µL, 10 mM final concentration). To end the experiment Triton X-100 detergent (50 µL, 7:1 (*v*/*v*) $H_2O$/DMSO) was added to lyse the vesicles.

For DLS measurements, 100 µL aliquots were taken before fueling ($t$ = 0), after fueling ($t$ = 10 min), and once again after detergent. DLS samples were diluted to 1 mL in disposable PMMA cuvettes, allowed to equilibrate at 25 °C for 90 s, then data was collected with an average from 3 measurements.

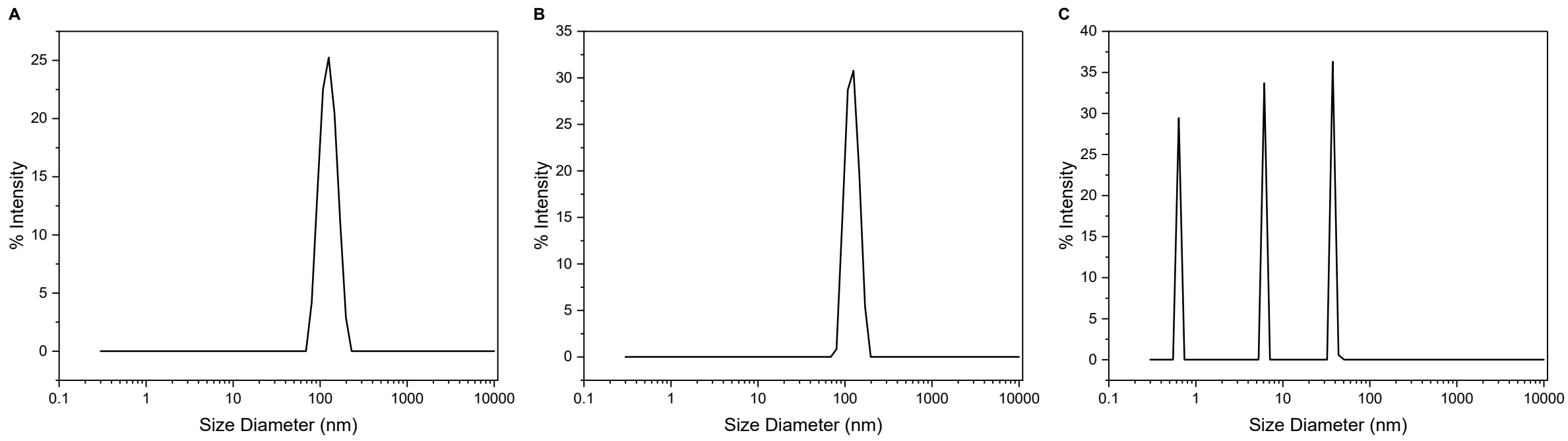


**Figure S40**. DLS measurement plots of POPC LUVs (0.1 mM, mean diameter 100 nm, Internal: 200 mM NaCl, ± 50 mM **1**, 100 mM MOPS and 3 mM HPTS, pH 6.5. External: 200 mM NaCl, ± 50 mM **1** and 100 mM MOPS pH 6.5). **A** before 10 mM EDC·MeI fuel ($t$ = 0), **B** after fueling ($t$ = 10 min), and **C** after detergent.

### S4.3.4 Active Transport of 1 vs 3 Across Lipid Bilayers

Here, HPTS transport assays were performed to compare the different proton gradients generated when transporting **1** or **3**

Thin lipid films of POPC (266 µL, 25 mg/mL in chloroform) were used to prepare POPC LUVs (3.5 mL, 2.5 mM, 100 nm) with an internal solution of 200 mM NaCl, 100 mM MOPS and 3 mM HPTS, 50 mM **1** or **3**, buffered to pH 6.5 and an external solution of 200 mM NaCl, 50 mM **1** or **3** and 100 mM MOPS again buffered to pH 6.5, using the general vesicle synthesis procedure described above.

In the transport experiments, the POPC LUVs (100 µL) were suspended in the external buffer solution (2350 µL) then EDC·MeI fuel (50 µL, 10 mM final concentration) was added at $t$ = 0; giving a standard volume (2.5 mL) and a final lipid concentration of 0.1 mM. The change in fluorescence was monitored over time for 20 min and Triton X-100 detergent (50 µL, 7:1 (*v*/*v*) $H_2O$/DMSO) was used to lyse the vesicles and terminate the assay.

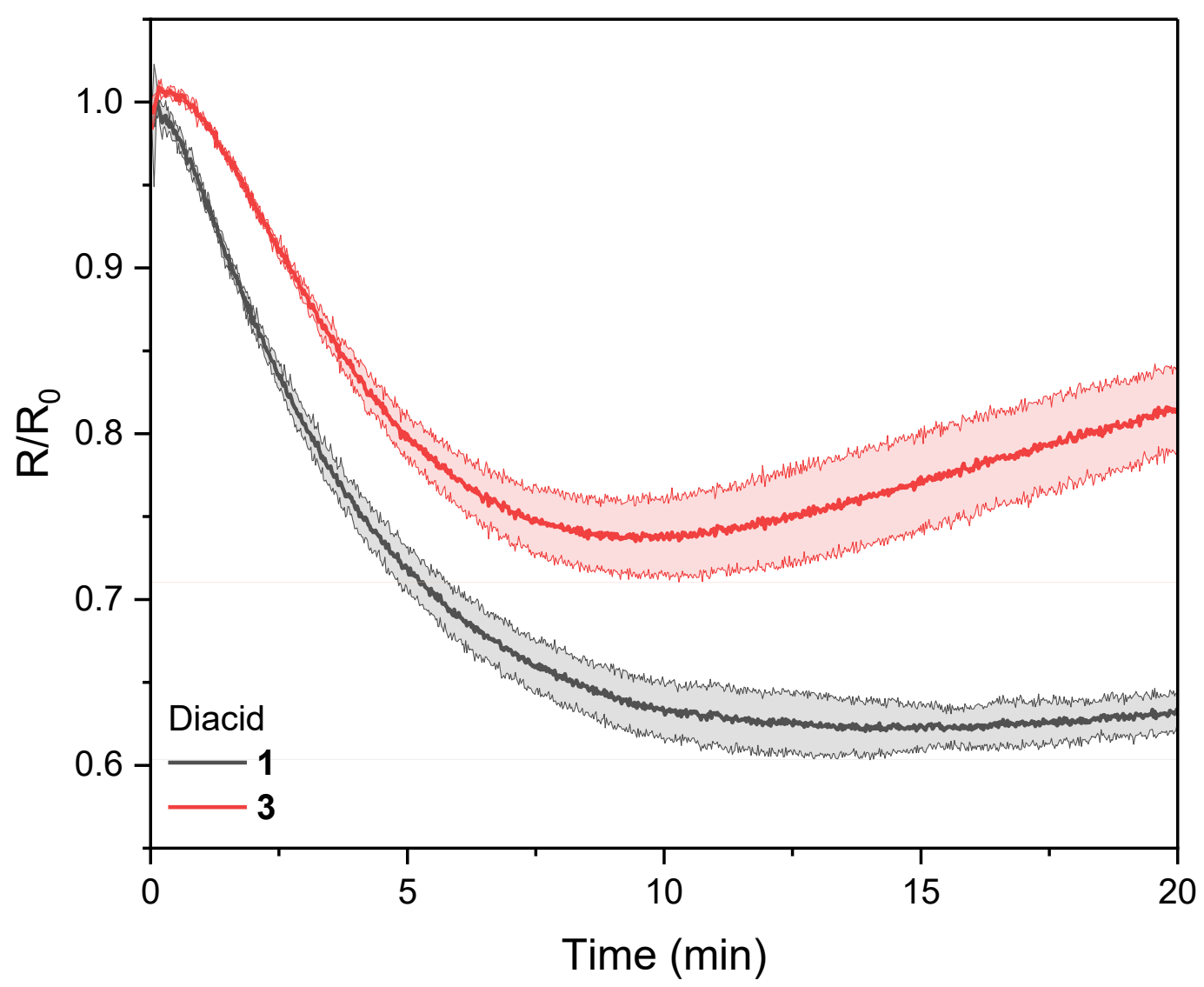


**Figure S41**. Active Transport of **1** or **3** across POPC LUVs (0.1 mM, 100 nm) upon addition of EDC·MeI fuel (50 μL, 10 mM final concentration) at *t* = 0. Internal: 200 mM NaCl, 50 mM **1** or **3**, 100 mM MOPS and 3 mM HPTS, pH 6.5. External: 200 mM NaCl, 50 mM **1** or **3** and 100 mM MOPS pH 6.5.

## S4.4. Calculations

### S4.4.1 Vesicle Volume Calculations

The encapsulated aqueous volume of 100 nm POPC large unilamellar vesicles (LUVs) was calculated assuming spherical, unilamellar geometry in the same manner as Section 3.6.1. Vesicles prepared by extrusion through 100 nm polycarbonate membranes were assigned an outer radius of $r_{ex}$ = 50.0 nm. A bilayer thickness of $\delta$ = 4.02 nm was used for POPC, consistent with reported structural data,[S7a–g] giving an interior (aqueous lumen) radius of $r_{in} = r_{ex} - \delta$ = 45.98 nm. The interior volume per vesicle is therefore:

$V_i = (4/3)\pi r_{in}^3 = 4.07 \times 10^{-13}$ µL (407,189 nm$^3$) (Equation S55)

The outer and inner leaflet surface areas are $A_o = 4\pi r_{ex}^2$ = 31,416 nm² and $A_i = 4\pi r_{in}^2$ = 26,567 nm², respectively. Using an area per POPC lipid of $a_L$ = 0.643 nm²,[S7a–g] the outer and inner leaflet lipid counts are $N_{outer}$ = 48,900 and $N_{inner}$ = 41,300, giving a total of $N_{lipid}$ = 90,200 lipids per vesicle.

At a POPC lipid concentration of 0.1 mM in a sample volume of 2.5 mL during a typical active transport experiment, the total moles of lipid are $n = 2.50 \times 10^{-7}$ mol, corresponding to $N_{ves} = nN_A / N_{lipid} = 1.67 \times 10^{12}$ vesicles. The total encapsulated aqueous volume is:

$V_{int} = N_{ves} \times V_i$ = 0.68 µL (Equation S56)

This corresponds to an interior volume fraction of $\varphi_{in} = V_{int} / V_{total} = 2.88 \times 10^{-4}$ (i.e., 0.027% of the 2.5 mL sample is encapsulated aqueous phase). The complementary extravesicular volume is $V_{ext}$ = 2499.32 µL. These volume assignments are used in all subsequent thermodynamic calculations

### S4.4.2 Transmembrane Potential Calculations

The transmembrane electrochemical H⁺ potential ($\Delta E_{H^+}$) generated through the active transport of diacid **1**, can be calculated using the Nernst equation:

$$\Delta E = \frac{RT}{zF} ln\left(\frac{[\mathrm{H}^+]_{t=0}}{[\mathrm{H}^+]_{t=10\,min}}\right) = \frac{RT}{F} ln(\Delta pH) \quad \text{(Equation S57)}$$

where $R$ = 8.314 J mol$^{-1}$ K$^{-1}$, $T$ = 298.15 K, $F$ = 96,485 C mol$^{-1}$, and ΔpH is evaluated at a given time point, typically $t$ = 0 (before fuel) and $t$ = 10 (after fuel and gradient generation).

Equation S57 reduces to ΔE / mV = 59.16 × ΔpH. A negative ΔpH (acidification of the interior) therefore corresponds to a negative ΔE. Hence, adding 20 mM EDC·MeI during active transport assays (Section S5.4) leads to a transmembrane pH gradient of ~0.32 corresponding to $\Delta E_{H^+}$ = −17.0 mV.

### S4.4.3 Quantification of pumped species

The observed pH at $t$ = 0 and $t$ = 10 min could be used further to calculate the concentration of maleic anhydride **2** pumped into the vesicle lumen accounting for the buffered solution of MOPS, HPTS and the pre-existing pool of **1**.

Upon hydrolysis, each mole of **2** formally converts to one mole of fully protonated **1** ($H_2A$), delivering two moles of titratable protons to the system. These protons are absorbed by the

conjugate bases present (deprotonated MOPS, HPTS, and the $HA^{-}/A^{2-}$ forms of **1**) until a new equilibrium is reached at $pH_{obs}$.

Conservation of total titratable protons ($[H^+]_{total}$) imposes $Pool_{(t\,=\,10\,min)} - Pool_{(t\,=\,0)} = [\mathbf{2}]_{(t\,=\,0)} \cdot [\mathbf{2}]_{pumped}$, where Pool = [MOPS-H] + [HPTS-H] + 2·$[H_2A]$ + $[HA^-]$. Each monoprotic buffer is distributed between its protonated and deprotonated forms according to the Henderson–Hasselbalch relation, and maleate is distributed over $H_2A$, $HA^-$ and $A^{2-}$ via its two acidity constants ($pK_{a1}$ = 1.94, $pK_{a2}$ = 6.22). Defining $\beta(pH) = (2h^2 + h{\cdot}K_{a1})/(h^2 + h{\cdot}K_{a1} + K_{a1}K_{a2})$, i.e. the average number of protons carried per mole of maleate at a given pH, and noting that the total maleate concentration after addition equals $[\mathbf{1}]_{(t\,=\,0)} + [\mathbf{2}]_{pumped}$, the mass balance can be solved in closed form:

$$[\mathbf{2}]_{pumped} = \frac{\{\Delta[\mathrm{MOPS{-}H}] + \Delta[\mathrm{HPTS{-}H}] + [\mathbf{1}]_{(t\,=\,0)} \cdot (\beta_{(t\,=\,10\,\mathrm{min})} - \beta_{(t\,=\,0)})\}}{2 - \beta_{(t\,=\,10\,\mathrm{min})}} \quad \text{(Equation S58)}$$

where $[\mathbf{1}]_{(t\,=\,0)}$ is the starting concentration of **1** (50 mM) when LUVs are synthesised, and the change (Δ) in HPTS-H and MOPS-H were calculated using the $pK_a$ values of MOPS (7.20), HPTS (7.30), the pH at $t$ = 0 and $t$ = 10 min along with their initial concentrations of 3 mM and 100 mM respectively.

Here, the pH decreases of ~0.32 after ~10 min with 20 mM EDC·MeI fuel is consistent with an increase in the intravesicular $[\mathbf{1}_{int}]$ of 12 mM. Factoring in the total intravesicular volume (0.72 μL) this equates to 8.8 nmol of **1** transported.

### S4.4.4 Stored Energy Calculations

Using the concentration of maleic anhydride **2** calculated above, the thermodynamic free energy stored in the non-equilibrium concentration distribution of protonatable species across the vesicle membrane was calculated according to an established method through the following equation:

$$G_{stored} = RT \cdot \frac{V_{int} \cdot \left([\mathbf{1}]_{int} \ln\frac{[\mathbf{1}]_{int}}{[\mathbf{1}]_{t\,=\,0}} - ([\mathbf{1}]_{int} + [\mathbf{1}]_{t\,=\,0})\right) + V_{ext} \cdot \left([\mathbf{1}]_{ext} \ln\frac{[\mathbf{1}]_{ext}}{[\mathbf{1}]_{t\,=\,0}} - ([\mathbf{1}]_{ext} + [\mathbf{1}]_{t\,=\,0})\right)}{V_{int} + V_{ext}} \quad \text{(Equation S59)}$$

Here, the stored free energy density (J $L^{-1}$) with respect to total volume is given considering the two compartments. Thus, $V$ represents the intra- and extravesicular volumes for 100 nm POPC LUVs calculated prior (Section S5.1.1, $V_{int} = 7.20 \times 10^{-7}$ L and $V_{ext} = 2.499 \times 10^{-3}$ L. $[\mathbf{1}]_{(t\,=\,0)}$ represents the concentration of diacid **1** at equilibrium while $[\mathbf{1}]_{int}$ and $[\mathbf{1}]_{ext}$ correspond to the concentration of diacid **1** in the intra- or extravesicular either before or after fueling ($t$ = 0 or $t$ = 10 min respectively).

For example, with 20 mM EDC·MeI fuel the maximum stored energy of the transmembrane Δ[**1**] gradient in this experiment is 3.4 J $L^{-1}$ (internal vesicle volume), or $9.8 \times 10^{-4}$ J $L^{-1}$ when expressed per total system volume (Equation S59)

These calculations are implemented in the supplemental spreadsheet.

## S4.5. Cascade Reactions

To demonstrate that the active transport of **1** had indeed generated a gradient of $H^+$ transporters, prodigiosin, monensin or CCCP, were added to dissipate said gradient.

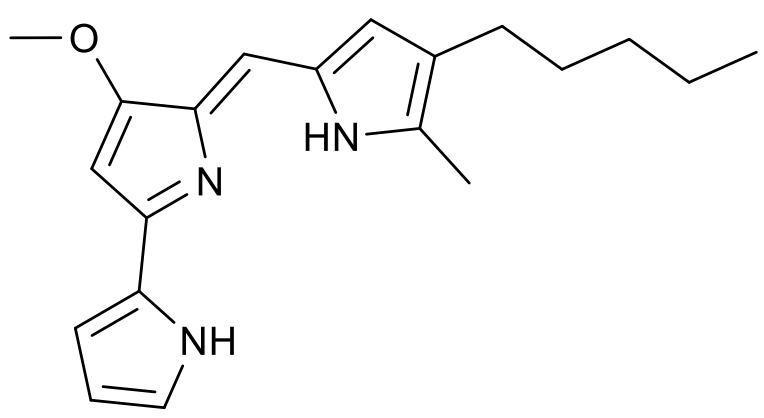

Prodigiosin

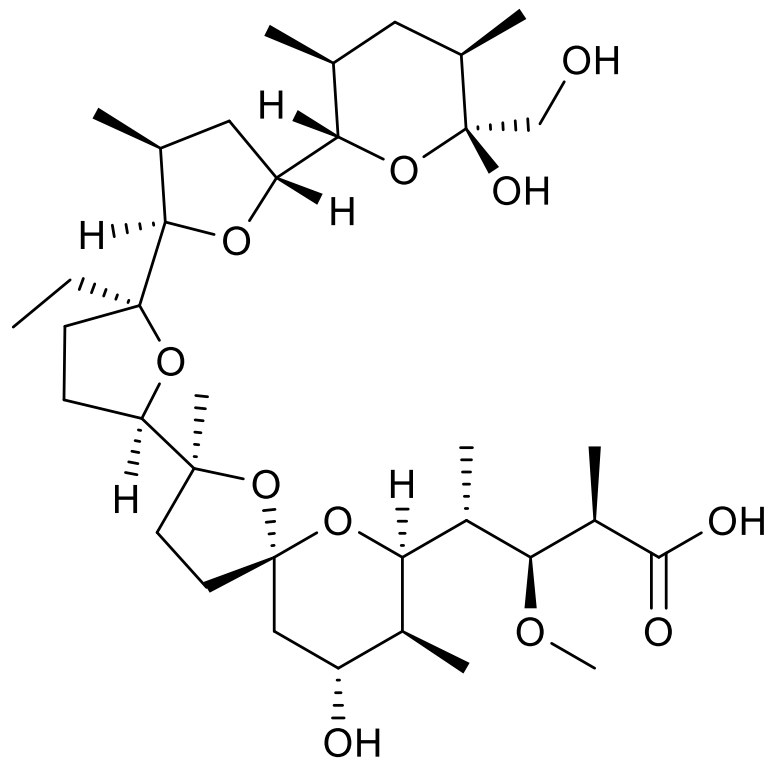

Monensin

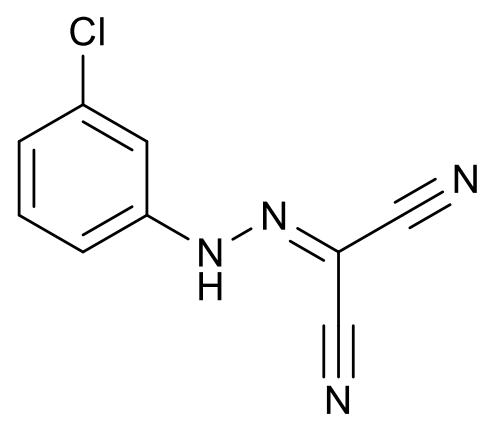

Carbonyl cyanyde m-chlorophenyl hydrazone (CCCP)

Thin lipid films of POPC (266 μL, 25 mg/mL in chloroform) were used to prepare POPC LUVs (3.5 mL, 2.5 mM, 100 nm) with an internal solution of 200 mM NaCl, 100 mM MOPS and 3 mM HPTS, ± 50 mM **1**, buffered to pH 6.5 and an external solution of 200 mM NaCl, ±50 mM **1** and 100 mM MOPS again buffered to pH 6.5, using the general vesicle synthesis procedure described above.

In the transport experiments, the POPC LUVs (100 μL) were suspended in the external buffer solution (2350 μL) then EDC·MeI fuel (50 μL, 10 mM final concentration) was added at *t* = 0; giving a standard volume (2.5 mL) and a final lipid concentration of 0.1 mM. At *t* = 10 mins, a transporter was added and the change in fluorescence was monitored for a further 10 mins. Triton X-100 detergent (50 μL, 7:1 (*v*/*v*) $H_2O$/DMSO) was added at *t* = 20 min to lyse the vesicles and terminate the assay. The original data is presented below, prior to calculation of the intravesicular pH, for completion.

### S4.5.1 $H^+/Cl^-$ Prodigiosin

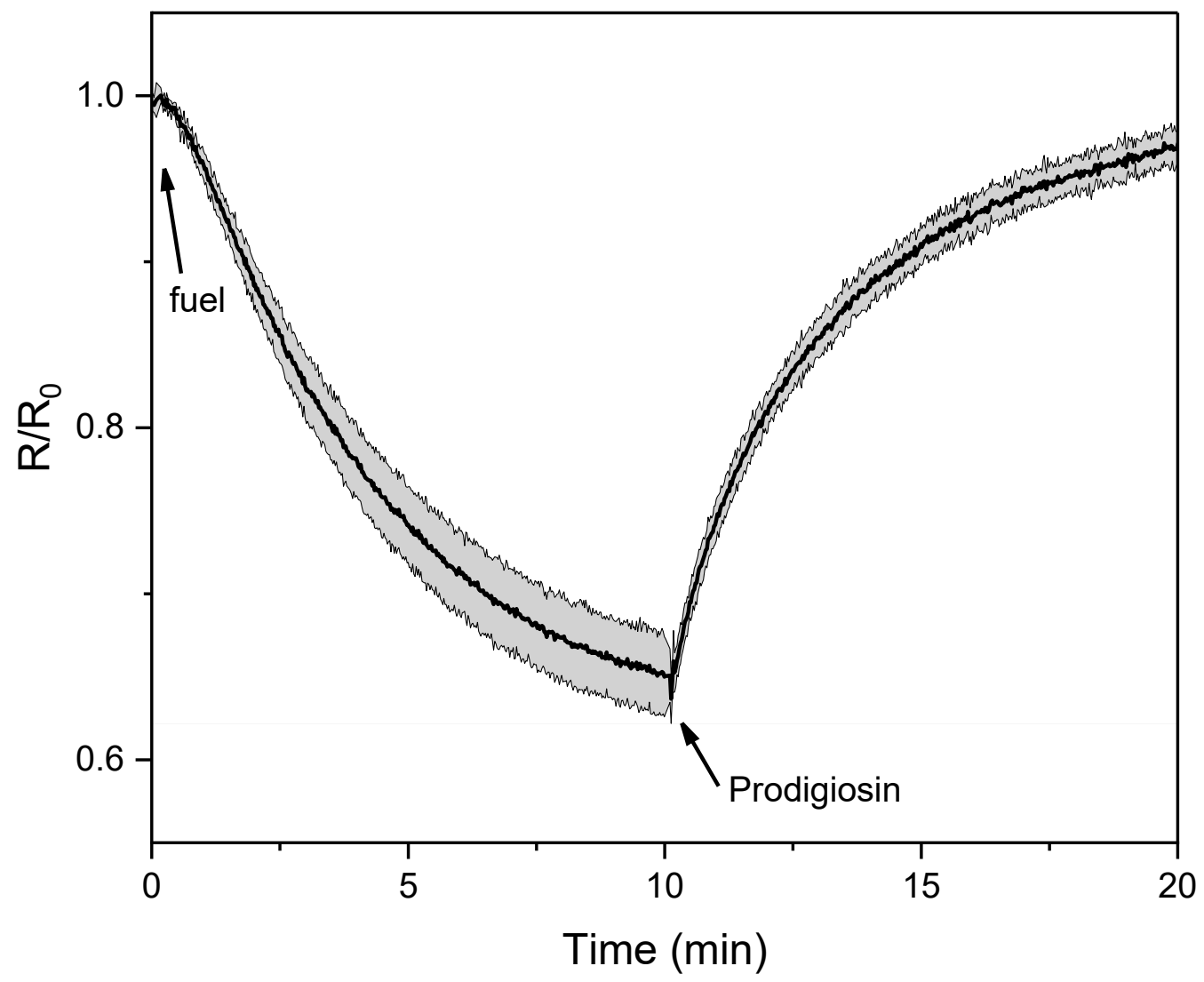


**Figure S42**. Change in fluorescence intensity of intravesicular HPTS over time ($\lambda_{em}$ = 510 nm; $\lambda_{ex1}$ = 405 nm, $\lambda_{ex2}$ = 460 nm) after addition of fuel ([EDC·MeI]$_0$ = 10 mM) at $t$ = 0, then addition of $H^+/Cl^-$ symporter prodigiosin at $t$ = 10 min (0.0001 mol% with respect to lipid). POPC LUVs (0.1 mM, 2.5 mL, 100 nm) were prepared with diacid **1** (internal: [HPTS] = 3 mM, [**1**]$_0$ = 50 mM, [NaCl] = 200 mM, [MOPS buffer] = 100 mM, ca. pH 6.5; extravesicular solution: [**1**]$_0$ = 50 mM, [NaCl] = 200 mM, [MOPS buffer] = 100 mM, ca. pH 6.5). Shaded area corresponds to the SD measured from 3 repeats.

### S4.5.2 $Na^+/H^+$ Antiporter Monensin

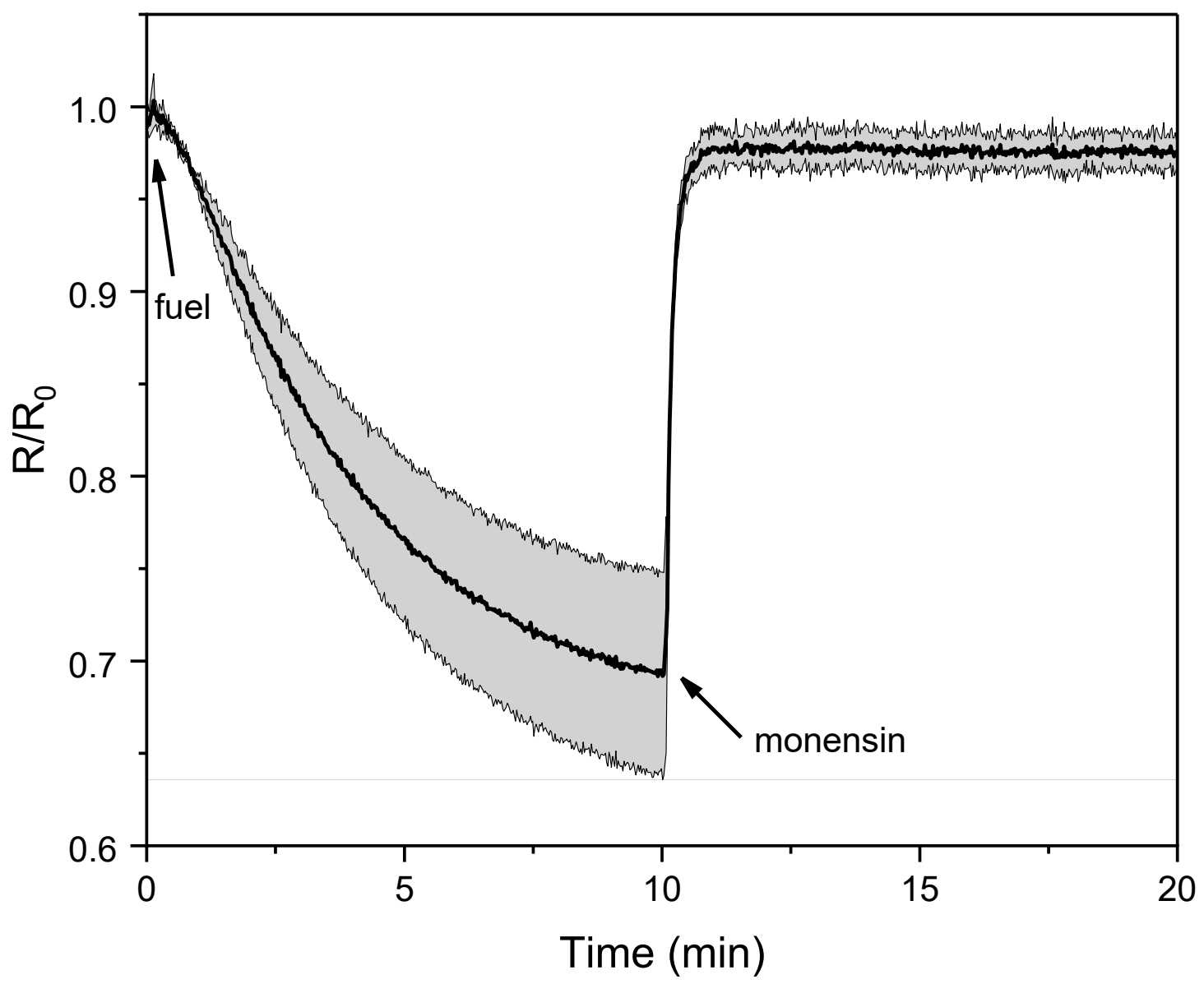


**Figure S43**. Change in fluorescence intensity of intravesicular HPTS over time ($\lambda_{em}$ = 510 nm; $\lambda_{ex1}$ = 405 nm, $\lambda_{ex2}$ = 460 nm) after addition of fuel ([EDC·MeI]$_0$ = 10 mM) at $t$ = 0, then addition of $H^+/Na^+$ antiporter monensin at $t$ = 10 min (0.01 mol% with respect to lipid). POPC LUVs (0.1 mM, 2.5 mL, 100 nm) were prepared with diacid **1** (internal: [HPTS] = 3 mM, [**1**]$_0$ = 50 mM, [NaCl] = 200 mM, [MOPS buffer] = 100 mM, ca. pH 6.5; extravesicular solution: [**1**]$_0$ = 50 mM, [NaCl] = 200 mM, [MOPS buffer] = 100 mM, ca. pH 6.5). Shaded area corresponds to the SD measured from 3 repeats.

### S4.5.3 Electrogenic $H^+$ Transporter CCCP

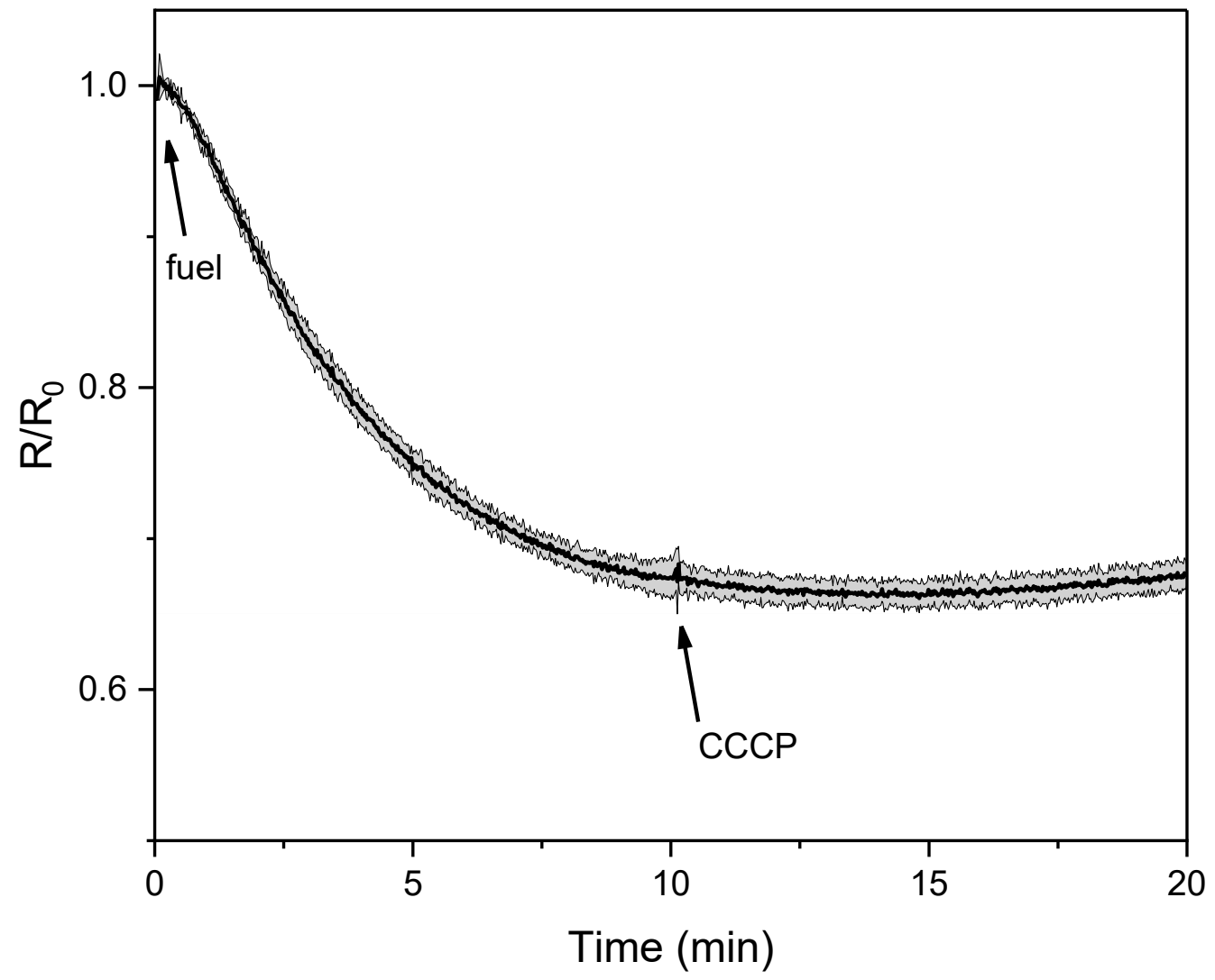


**Figure S44**. Change in fluorescence intensity of intravesicular HPTS over time ($\lambda_{em}$ = 510 nm; $\lambda_{ex1}$ = 405 nm, $\lambda_{ex2}$ = 460 nm) after addition of fuel ([EDC·MeI]$_0$ = 10 mM) at *t* = 0, then addition of $H^+$ transporter CCCP at *t* = 10 min (0.1 mol% with respect to lipid). POPC LUVs (0.1 mM, 2.5 mL, 100 nm) were prepared with diacid **1** (internal: [HPTS] = 3 mM, [**1**]$_0$ = 50 mM, [NaCl] = 200 mM, [MOPS buffer] = 100 mM, ca. pH 6.5; extravesicular solution: [**1**]$_0$ = 50 mM, [NaCl] = 200 mM, [MOPS buffer] = 100 mM, ca. pH 6.5). Shaded area corresponds to the SD measured from 3 repeats.

### S4.5.4 Quantification of Ion Gradient and Transduction Efficiency

According to prior procedures (Section S5.1.2, see supplemental spreadsheet) the transmembrane potentials, concentration of ions pumped and the stored free energy, at *t* = 0 (equilibrium), *t* = 10 (after fueling) and *t* = 20 min (secondary active transport) could be calculated.

**Table S7**. Measured pH gradients ($\Delta pH_{in}$), membrane potential ($\Delta E_{H^+}$), and internal ion concentrations under primary and secondary active transport conditions.

| | **Primary Active Transport**[a] | | | **Secondary Active Transport (Equilibration)**[b] | | | |
|---|---|---|---|---|---|---|---|
| | $\Delta pH_{in}$ | $\Delta E_{H^+}$ (mV) | $\Delta$[**1**]$_{int}$ (mM) | $\Delta pH_{in}$ | $\Delta E_{H^+}$ (mV) | $[Cl^-]_{int}$ (mM) | $[Na^+]_{int}$ (mM) |
| Prodigiosin ($H^+/Cl^-$)[c] | −0.185 | −11.0 | +7.07 | +0.173 | +11.9 | 188 | – |
| Monensin ($H^+/Na^+$)[d] | −0.154 | −9.11 | +5.86 | +0.148 | +10.0 | – | 210 |
| CCCP ($H^+$)[e] | −0.167 | −9.88 | +6.37 | – | – | – | – |

[a]EDC·MeI (10 mM) added at *t* = 0, values reported at *t* = 10 mins prior to transporter addition. [b]Transporters added at *t* = 0, values reported after 20 mins. [c]Prodigiosin (0.0001 mol%). [d]Monensin (0.01 mol%). [e]CCCP (0.1 mol%).

The energy stored at *t* = 20 min in the form of $\Delta[Cl^-]$ equals 0.91 J $L^{-1}$, while the energy stored as $[Na^+]$ was 0.64 J $L^{-1}$ (see supplemental spreadsheet).

The stored free energy after fueling (*t* = 10 min) and secondary active transport (*t* = 20 min) were used to calculate the transduction efficiency η (see supplemental spreadsheet):

$$\eta = \frac{\Delta G_{stored,t=20\ min}}{\Delta G_{stored,t=10}} \qquad \text{(Equation S60)}$$

## S4.6. Additional Control Experiments

### S4.6.1 Lipid Composition Variation

Here we explored the effect of changing the fluidity of the membrane, using DOPC vs. POPC with and without cholesterol. Vesicles were synthesised according to the general procedure with an internal solution of 200 mM NaCl, 100 mM MOPS and 3 mM HPTS, 50 mM **1**, buffered to pH 6.5 and an external solution of 200 mM NaCl, 50 mM **1** and 100 mM MOPS again buffered to pH 6.5.

DOPC: lipid film (275 µL, 25 mg/mL in chloroform) was used to prepare DOPC LUVs (3.5 mL, 2.5 mM, 100 nm).

DOPC with cholesterol: lipid film (193 µL, 25 mg/mL in chloroform) and cholesterol (150 µL of 25 mM stock in chloroform: methanol 7:3) was used to prepare DOPC/cholesterol 7:3 LUVs (3.5 mL, 2.5 mM, 100 nm).

POPC with cholesterol: lipid film (186 µL, 25 mg/mL in chloroform) and cholesterol (150 µL of 25 mM stock in chloroform: methanol 7:3) was used to prepare POPC/cholesterol 7:3 LUVs (3.5 mL, 2.5 mM, 100 nm).

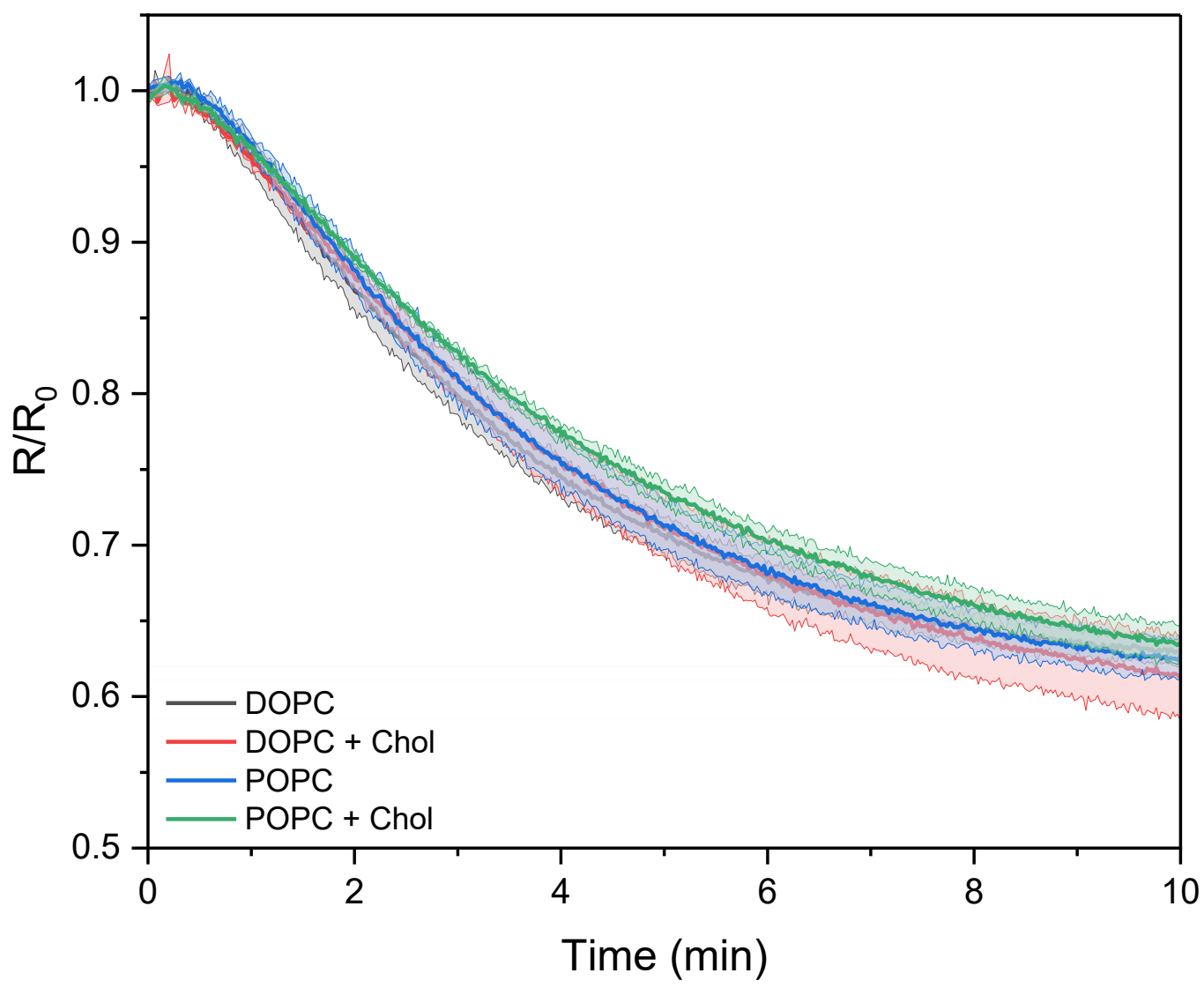


**Figure S45**. Active Transport of **1** across LUVs (0.1 mM, 100 nm) upon addition of EDC·MeI fuel (50 µL, 10 mM final concentration) at *t* = 0. Internal: 200 mM NaCl, 50 mM **1**, 100 mM MOPS and 3 mM HPTS, pH 6.5. External: 200 mM NaCl, 50 mM **1** and 100 mM MOPS pH 6.5.

### S4.6.2 Variation of Vesicle Size

For kinetic fluorescence spectroscopy experiments 100 nm LUVs were chosen rather than the 200 nm LUVs as the $^1$H NMR spectroscopy studies (Section S3). Comparative HPTS active transport assays were completed.

A thin lipid film of POPC (266 µL, 25 mg/mL in chloroform) were used to prepare POPC LUVs (3.5 mL, 2.5 mM, 100 nm or 200 nm) with an internal solution of 200 mM NaCl, 100 mM MOPS and 3 mM HPTS, ±50 mM **1**, buffered to pH 6.5 and an external solution of 200 mM NaCl, ±50 mM **1** and 100 mM MOPS again buffered to pH 6.5, using the general vesicle synthesis procedure described above. The POPC LUVs (100 µL, 100 or 200 nm, 0.1 mM final concentration) were suspended in external buffer solution (2350 µL) prior to addition of EDC·MeI fuel (50 µL, 10 mM final concentration) at *t* = 0. To end the experiment Triton X-100 detergent (50 µL, 7:1 (v/v) $H_2O$-DMSO) was added to lyse the vesicles.

As expected, active transport was slower in larger 200 nm vesicles.[S19] Since the lipid concentration is fixed, 100 nm LUVs encapsulate less aqueous volume, therefore, the same membrane flux acts on a smaller lumen volume, leading to a faster apparent HPTS response.

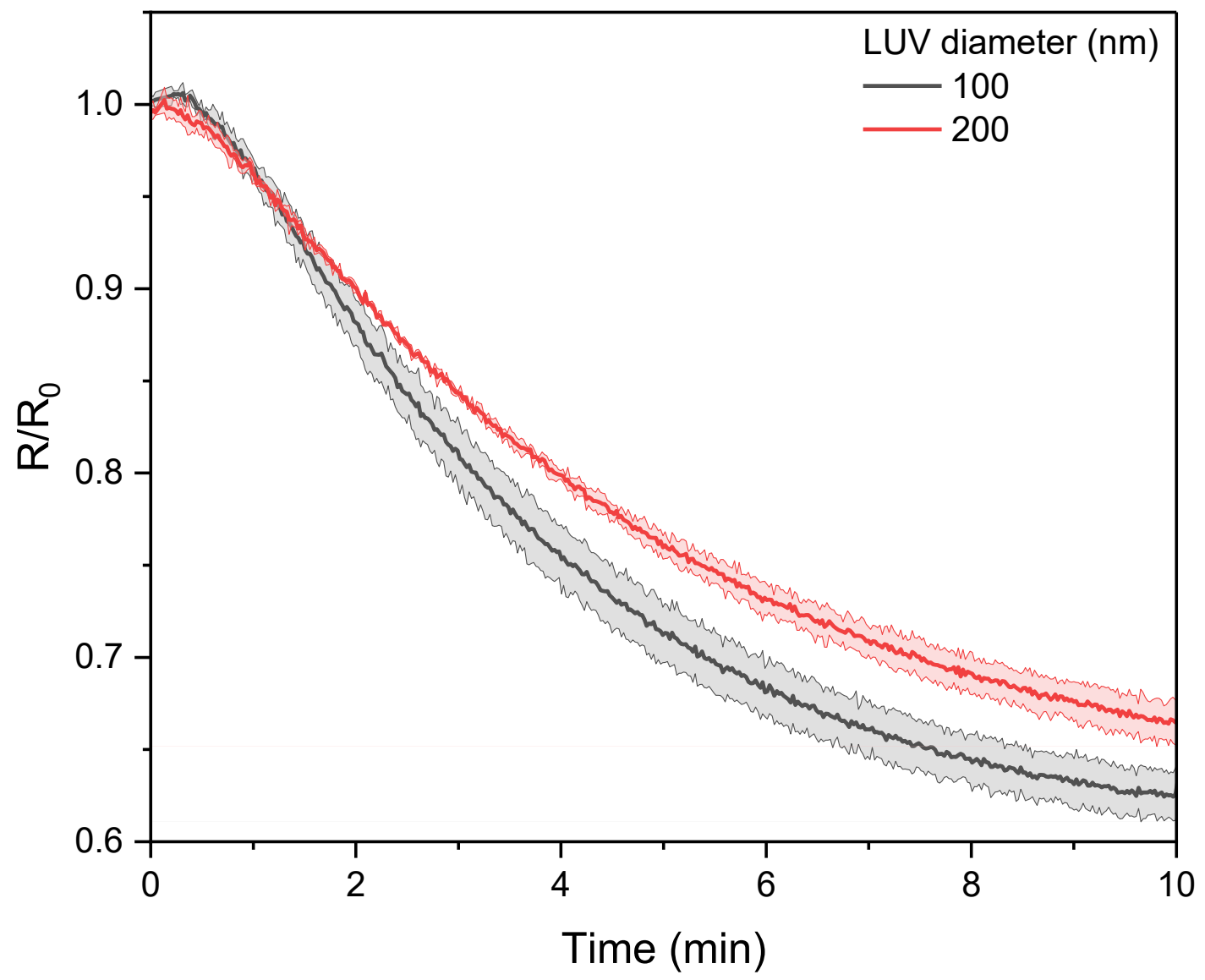


**Figure S46**. Active Transport of **1** across POPC LUVs (0.1 mM lipid, 100 nm or 200 nm) upon addition of EDC·MeI fuel (50 µL, 10 mM final concentration) at *t* = 0. Internal: 200 mM NaCl, 50 mM **1**, 100 mM MOPS and 3 mM HPTS, pH 6.5. External: 200 mM NaCl, 50 mM **1** and 100 mM MOPS pH 6.5.

## S4.7. HPTS Active Transport Assays (EDC·HCl fuel)

### S4.7.1 Starting $pH_{int} > pH_{ext}$

When investigating the flux of protons with fluorescence spectroscopy we also investigated conditions that were built upon the prior $^1$H NMR investigation (Section S3), hence using vesicles synthesised with an initial pH gradient across the bilayer.

For these studies to be compatible with use of the fluorescent pH probe 8-hydroxypyrene-1,3,6-trisulfonic acid trisodium salt (HPTS, pKa ≈ 7.3), vesicle studies had to be carried out with higher interior and exterior pH, hence MOPS buffer was used instead of MES buffer (buffering window 6.5-7.9 vs. 5.5-6.7 respectively).

A thin lipid film of POPC (186 μL, 25 mg/mL in chloroform) and cholesterol (150 μL of 25 mM stock in chloroform: methanol 7:3) was used to prepare POPC/cholesterol 7:3 LUVs (3.5 mL, 2.5 mM, 100 nm) with an internal solution of ±50 mM **1**, 200 mM NaCl, 100 mM MOPS and 3 mM HPTS, buffered to pH 7.5 and an external solution of ±50 mM **1**, 200 mM NaCl and 100 mM MOPS buffered to pH 6.5, using the general vesicle synthesis procedure described above.

In the transport experiments, the LUVs (100 μL) were suspended in the external buffer solution (2350 μL), then EDC·HCl fuel (50 μL, 10 mM final concentration) was added at *t* = 1 min to initiate the experiment; giving a standard volume (2.5 mL) and a final lipid concentration of 0.1 mM. The change in fluorescence was monitored over time and Triton X-100 detergent (50 μL, 7:1 (v/v) $H_2O$-DMSO) was added at *t* = 11 min to lyse the vesicles and terminate the assay.

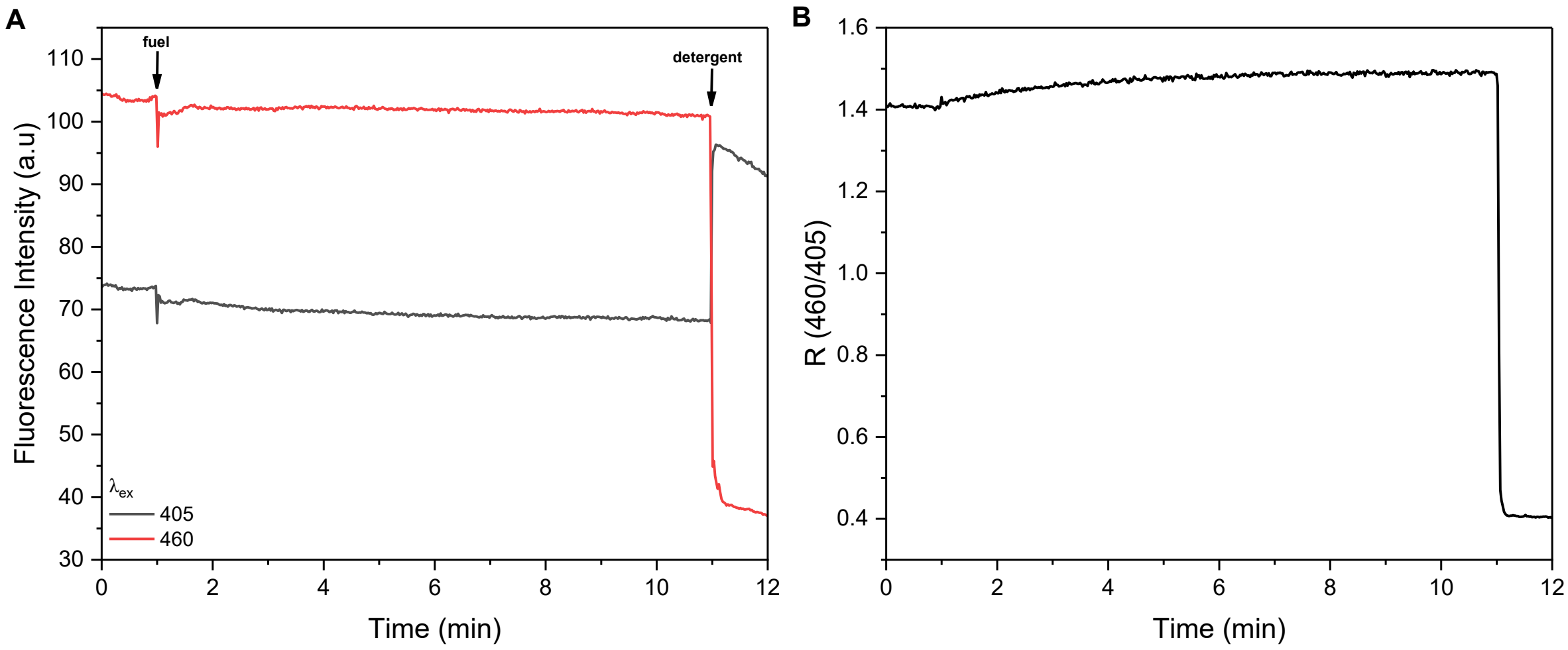


**Figure S47**. **A** Raw data showing the change in fluorescence over time ($\lambda_{em}$ = 510 nm; $\lambda_{ex1}$ = 405 nm, $\lambda_{ex2}$ = 460 nm) upon addition of EDC·HCl (50 μL, 10 mM final concentration) to POPC LUVs (0.1 mM, 100 nm, POPC:cholesterol 7:3). Internal: 200 mM NaCl, 100 mM MOPS and 3 mM HPTS, pH 7.5. External: 200 mM NaCl, and 100 mM MOPS pH 6.5. Vesicle lysis by Triton X-100 (50 μL). **B** Plot of the relative fluorescence intensity (R) over time.

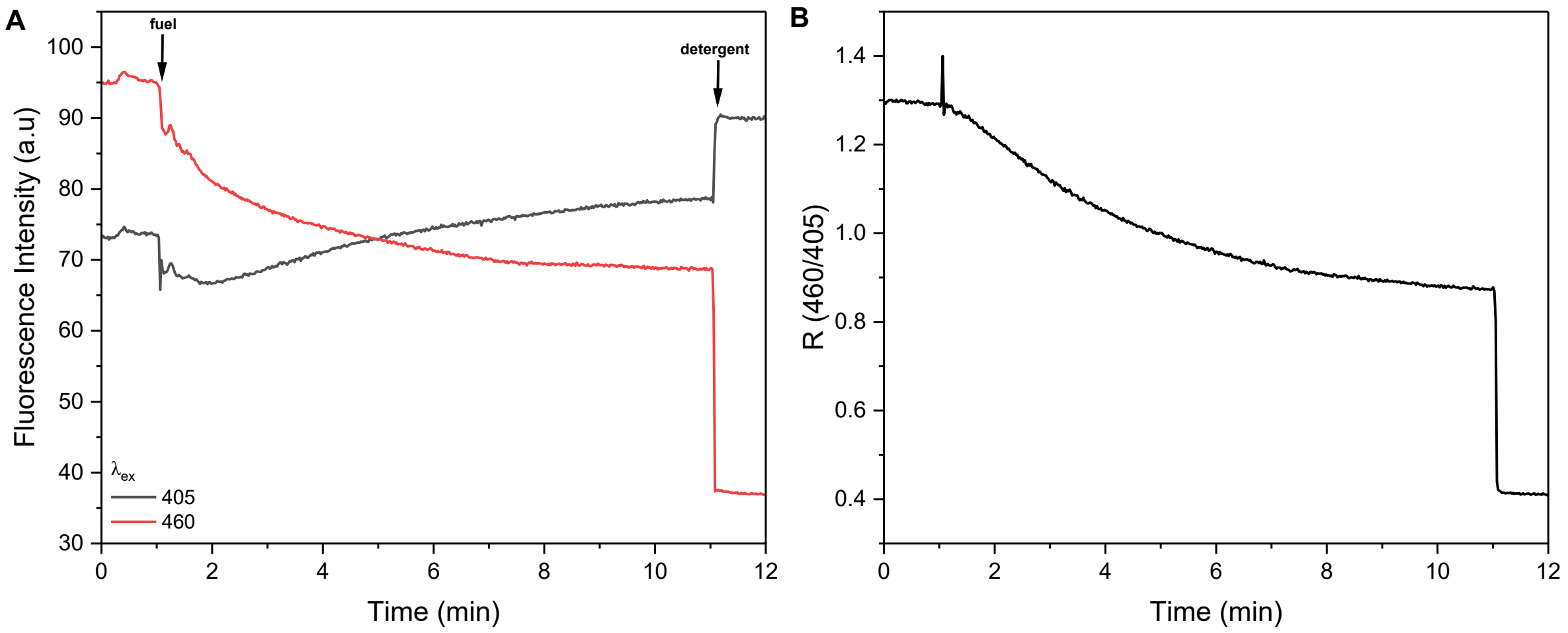


**Figure S48**. **A** Raw data showing the change in fluorescence over time ($\lambda_{em}$ = 510 nm; $\lambda_{ex1}$ = 405 nm, $\lambda_{ex2}$ = 460 nm) upon addition of EDC·HCl (50 µL, 10 mM final concentration) to POPC LUVs (0.1 mM, 100 nm, POPC:cholesterol 7:3). Internal: 200 mM NaCl, 50 mM **1**, 100 mM MOPS and 3 mM HPTS, pH 7.5. External: 200 mM NaCl, 50 mM **1** and 100 mM MOPS pH 6.5. Vesicle lysis by Triton X-100 (50 µL). **B** Plot of the relative fluorescence intensity (R) over time.

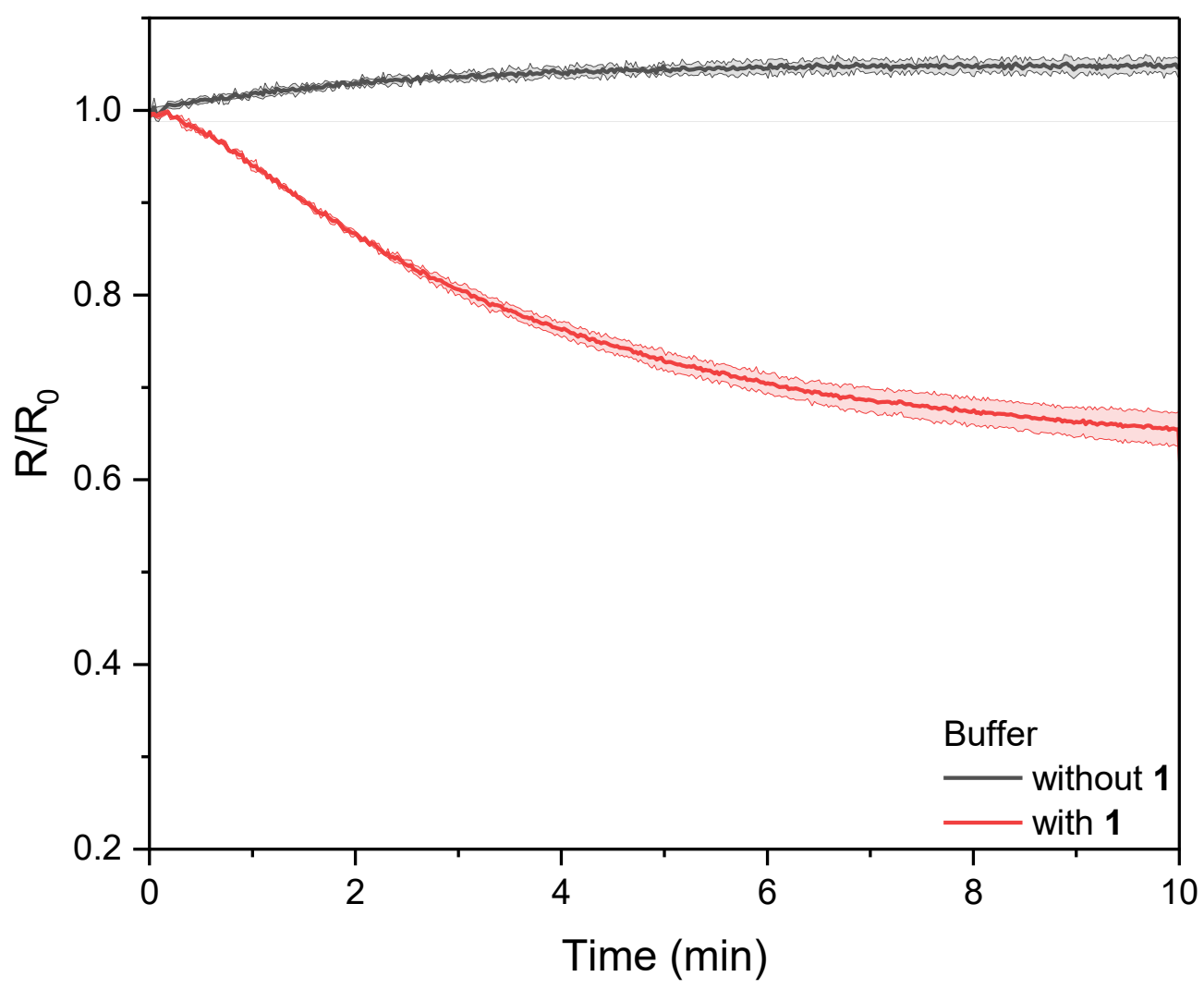


**Figure S49**. Active Transport across liposomes (0.1 mM vesicles, POPC:cholesterol 7:3) upon addition of EDC·HCl (50 µL, 10 mM final concentration) at $t$ = 0, with and without the presence of **1**. Internal: 200 mM NaCl, ± 50 mM **1**, 100 mM MOPS and 3 mM HPTS, pH 7.5. External: 200 mM NaCl, ± 50 mM **1** and 100 mM MOPS pH 6.5.

### S4.7.2 Starting $pH_{int} = pH_{ext}$

To investigate the dependence of active transport on the initial pH gradient, LUVs were synthesised with the same starting pH either side of the bilayer.

A thin lipid film of POPC (186 µL, 25 mg/mL in chloroform) and cholesterol (150 µL of 25 mM stock in chloroform/methanol 7:3, *v/v*) was used to prepare POPC/cholesterol 7:3 LUVs (3.5 mL, 2.5 mM, 100 nm) with an internal solution of ± 50 mM **1**, 200 mM NaCl, 100 mM MOPS and 3 mM HPTS, buffered to pH 6.5 and an external solution of ±50 mM **1**, 200 mM NaCl and 100 mM MOPS buffered to pH 6.5, using the general vesicle synthesis procedure described above.

In the transport experiments, the LUVs (100 µL) were suspended in the external buffer solution (2350 µL) then EDC·HCl fuel (50 µL, 10 mM final concentration) was added at 60 s to initiate the experiment; giving a standard volume (2.5 mL) and a final lipid concentration of 0.1 mM. The change in fluorescence was monitored over time then Triton X-100 detergent (50 µL, 7:1 (v/v) $H_2O$/DMSO) was used to lyse the vesicles and terminate the assay.

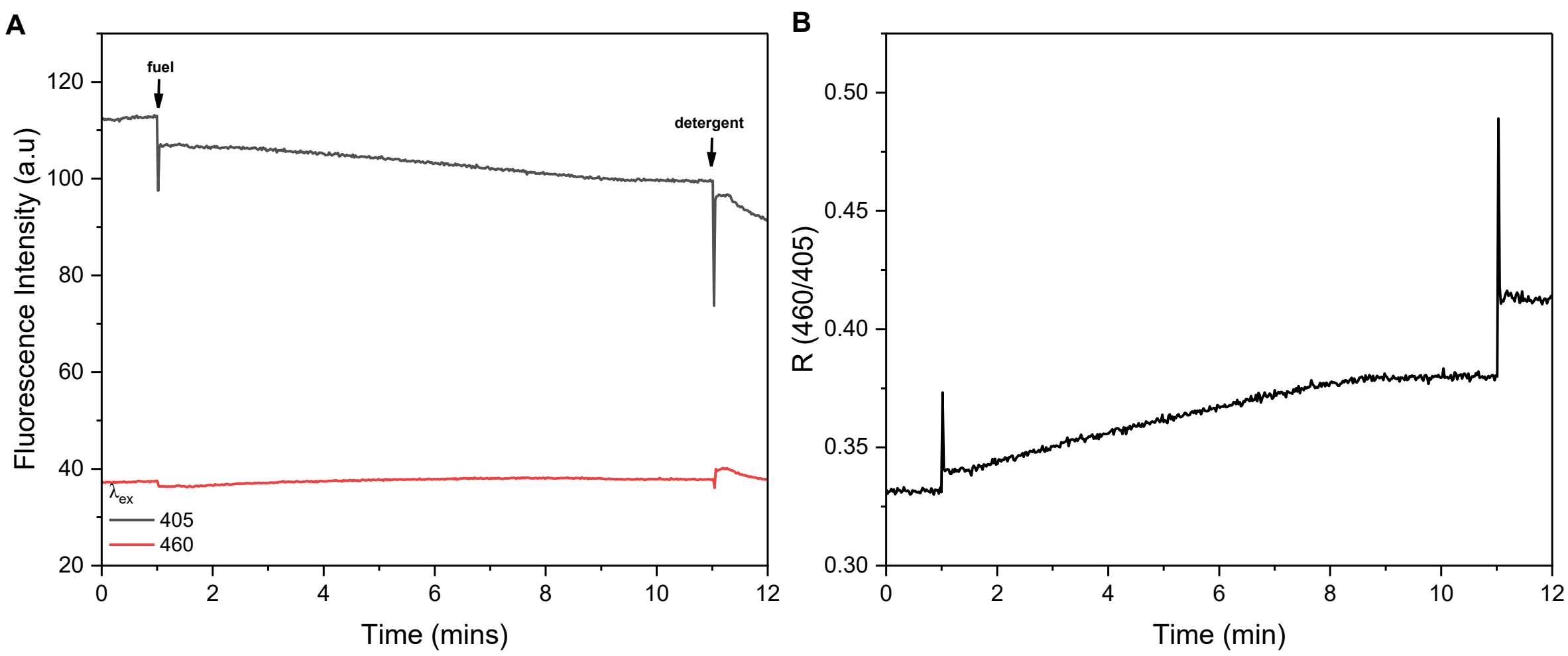


**Figure S50**. **A** Raw data showing the change in fluorescence over time ($\lambda_{em}$ = 510 nm; $\lambda_{ex1}$ = 405 nm, $\lambda_{ex2}$ = 460 nm) upon addition of EDC·HCl (50 µL, 10 mM final concentration) to LUVs (0.1 mM, 100 nm, POPC:cholesterol 7:3). Internal: 200 mM NaCl, 100 mM MOPS and 3 mM HPTS, pH 6.5. External: 200 mM NaCl, and 100 mM MOPS pH 6.5. Vesicle lysis by Triton X-100 (50 µL). **B** Plot of the relative fluorescence intensity (R) over time.

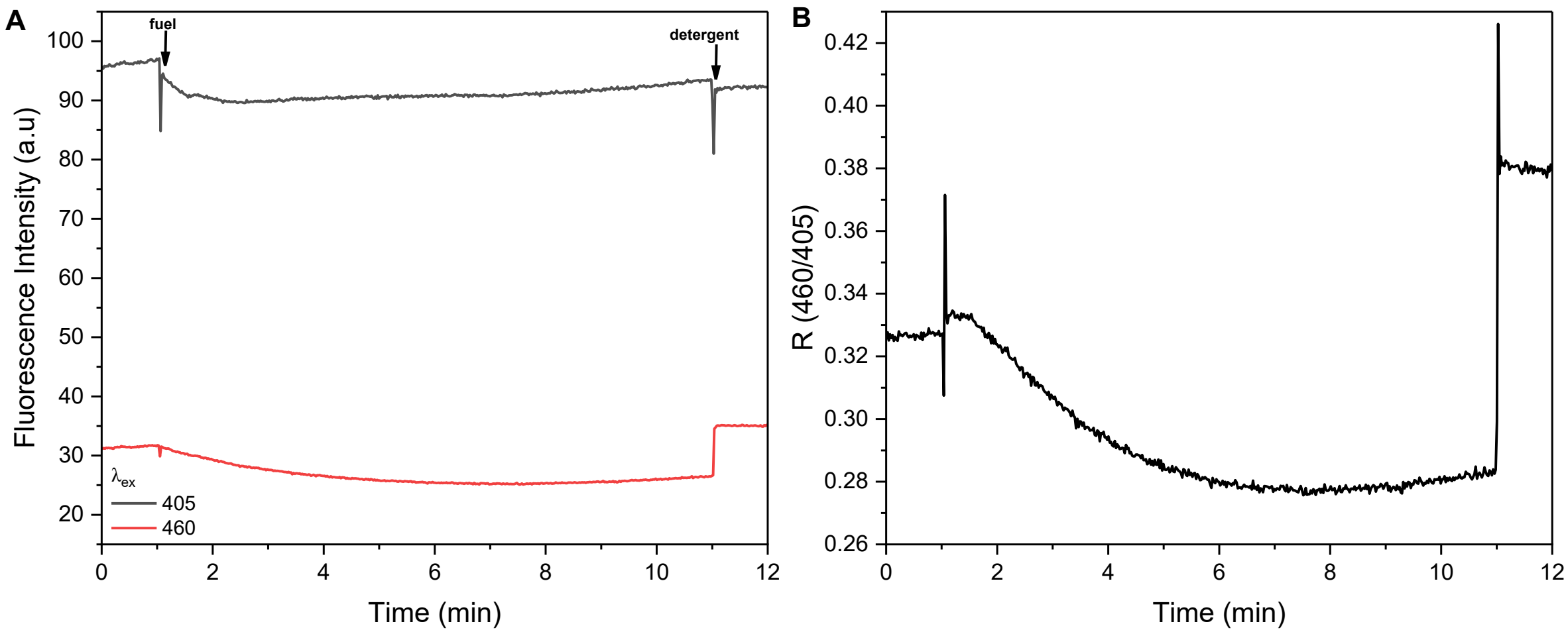


**Figure S51**. **A** Raw data showing the change in fluorescence over time ($\lambda_{em}$ = 510 nm; $\lambda_{ex1}$ = 405 nm, $\lambda_{ex2}$ = 460 nm) upon addition of EDC·HCl (50 µL, 10 mM final concentration) to POPC LUVs (0.1 mM, 100 nm, POPC:cholesterol 7:3). Internal: 200 mM NaCl, 50 mM **1**, 100 mM MOPS and 3 mM HPTS, pH 6.5. External: 200 mM NaCl, 50 mM **1** and 100 mM MOPS pH 6.5. Vesicle lysis by Triton X-100 (50 µL). **B** Plot of the relative fluorescence intensity (R) over time.

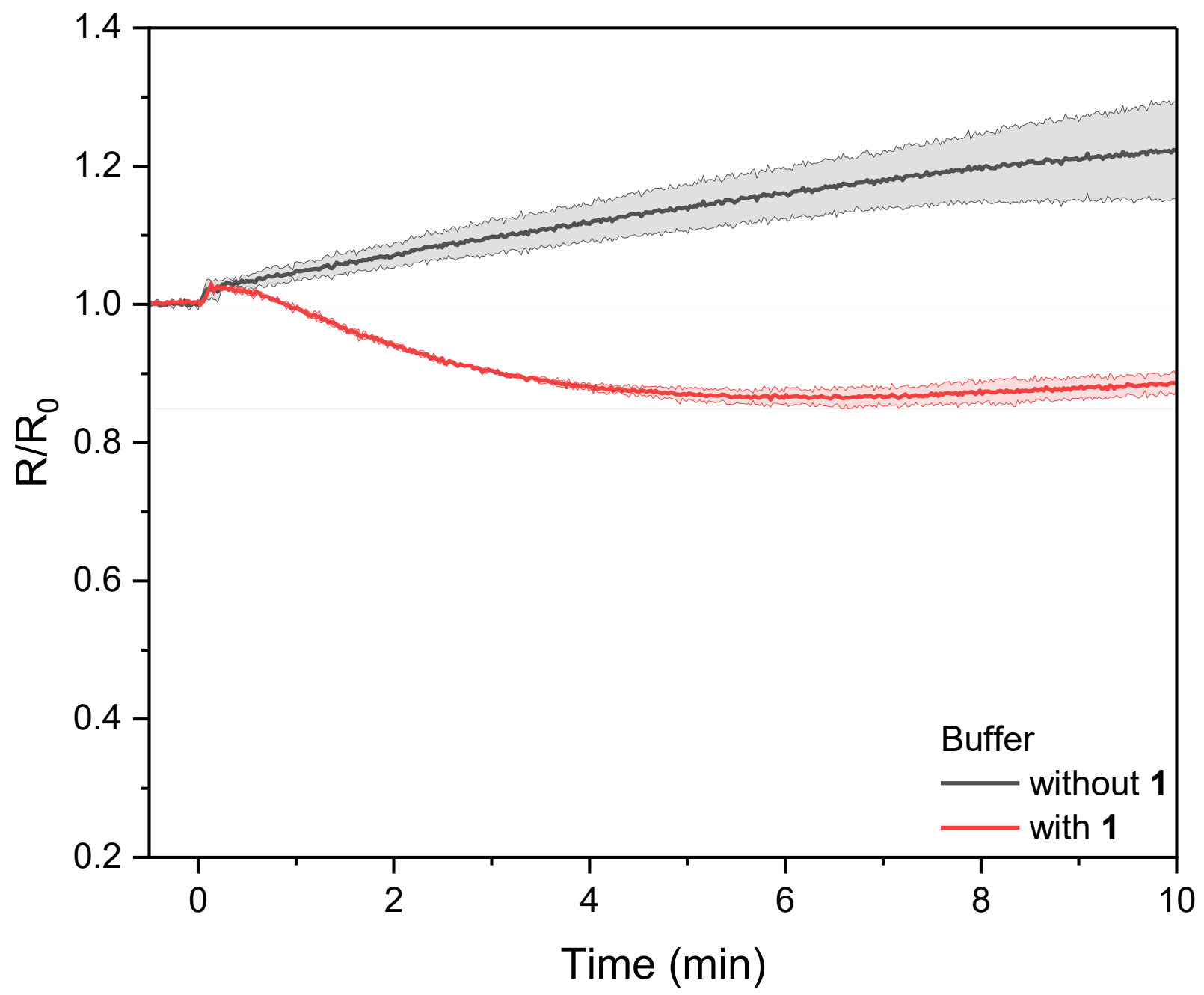


**Figure S52**. Active Transport across liposomes (0.1 mM vesicles, POPC:cholesterol 7:3) upon addition of EDC·HCl (50 µL, 10 mM final concentration) at *t* = 0, with and without the presence of **1**. Internal: 200 mM NaCl, ±50 mM **1**, 100 mM MOPS and 3 mM HPTS, pH 6.5. External: 200 mM NaCl, ±50 mM **1** and 100 mM MOPS pH 6.5.

Large drift in fluorescence intensity when EDC·HCl fuel was added to LUVs made without **1** at this pH. $^{1}$H NMR spectroscopy experiments (Section S3) experiments showed leakage of the fuel across the bilayer hence all investigations use EDC·MeI for fueling. Additionally, after fueling when using LUVs with **1** present, the gradient generated slowly started to dissipate after 6 min.

## S5. References


S1. Assmus, F., Ross, A., Fisher, H., Seelig, J., and Seelig, A. (2017). $^{31}P$ and $^{1}H$ NMR Studies of the Molecular Organization of Lipids in the Parallel Artificial Membrane Permeability Assay. Mol. Pharmaceutics 14, 284-295. DOI: 10.1021/acs.molpharmaceut.6b00889.

S2. Parallel Artificial Membrane Permeability Assay-BBB Kit (PMBBB-096) https://bioassaysys.com/parallel-artificial-membrane-permeability-assay-bbb-kit/

S3. Liang, K., Nicoli, F., Al Shehimy, S., Penocchio, E., Di Noja, S., Li, Y., Bonfio, C., Borsley, S., and Ragazzon, G. (2025). Catalysis-driven active transport across a liquid membrane. Angew. Chem. Int. Ed. 64, e202421234. DOI: 10.1002/anie.202421234.

S4. Sangchai, T., Al Shehimy, S., Penocchio, E., and Ragazzon, G. (2023). Artificial molecular ratchets: tools enabling endergonic processes. Angew. Chem. Int. Ed. 62, e202309501. DOI: 10.1002/anie.202309501.

S5. Aguilar, J. A., and Kenwright, S. J. (2016). Robust NMR water signal suppression for demanding analytical applications. Analyst 141, 236–242. DOI: 10.1039/C5AN02121A.

S6. Howe, E. N. W., and Gale, P. A. (2019). Fatty Acid Fueled Transmembrane Chloride Transport. J. Am. Chem. Soc. 141, 10654-10660. DOI: 10.1021/jacs.9b02116.

S7. Lipid structural parameters: (a) Heftberger, P., Kollmitzer, B., Heberle, F. A., Pan, J., Rappolt, M., Amenitsch, H., Kucerka, N., Katsaras, J., and Pabst, G. (2014). Global Small-Angle X-Ray Scattering Data Analysis for Multilamellar Vesicles: the Evolution of the Scattering Density Profile Model. J. Appl. Crystallogr. 47, 173–180. DOI: 10.1107/S1600576713029798. (b) Kučerka, N., Tristram-Nagle, S., and Nagle, J. F. (2006) Structure of Fully Hydrated Fluid Phase Lipid Bilayers with Monounsaturated Chains. J. Membrane Biol. 208, 193–202. DOI: 10.1007/s00232-005-7006-8. (c) Leftin, A., Molugu, Trivikram R., Job, C., Beyer, K., and Brown, M. F. (2014). Area per Lipid and Cholesterol Interactions in Membranes from Separated Local-Field $^{13}C$ NMR Spectroscopy. Biophys. J. 107, 2274–2286. DOI: 10.1016/j.bpj.2014.07.044. (d) Poger, D., and Mark, A. E. (2010). On the Validation of Molecular Dynamics Simulations of Saturated and cis-Monounsaturated Phosphatidylcholine Lipid Bilayers: A Comparison with Experiment. J. Chem. Theory Comput. 6, 325–336. DOI: 10.1021/ct900487a. (e) Smaby, J. M., Momsen, M. M., Brockman, H. L., and Brown, R. E. (1997). Phosphatidylcholine Acyl Unsaturation Modulates the Decrease in Interfacial Elasticity Induced by Cholesterol. Biophys. J. 73, 1492–1505. DOI: 10.1016/S0006-3495(97)78181-5. (f) Kukol, A. (2009). Lipid Models for United-Atom Molecular Dynamics Simulations of Proteins. J. Chem. Theory Comput. 5, 615–626. DOI: 10.1021/ct8003468. (g) Heller, H., Schaefer, M., and Schulten, K. (1993). Molecular Dynamics Simulation of a Bilayer of 200 Lipids in the Gel and in the Liquid Crystal Phase. J. Phys. Chem. 97, 8343–8360. DOI: 10.1021/j100133a034. (h) Pan, J., Tristram-Nagle, S., Kučerka, N., and Nagle, J.F. (2008). Temperature Dependence of Structure, Bending Rigidity, and Bilayer Interactions of Dioleophosphatidylcholine Bilayers. Biophys. J. 94, 117–124. DOI: 10.1529/biophysj.107.115691.

S8. Luzzati, V., and Husson, F. (1962). The Structure of the Liquid-Crystalline Phases of Lipid-Water Systems. J. Cell Biol. 12, 207-219. DOI: 10.1083/jcb.12.2.207.

S9. King, E. L., and Altman, C. (1956). A schematic method of deriving the rate laws for enzyme-catalyzed reactions. J. Phys. Chem. 60, 1375-1378. DOI: 10.1021/j150544a010.

S10. Hill, T. L. (1966). Studies in irreversible thermodynamics. IV. Diagrammatic representation of steady state fluxes for unimolecular systems. J. Theor. Biol. 10, 442–459. DOI: 10.1016/0022-5193(66)90137-8.

S11. Nam, K. M., Martinez-Corral, R., and Gunawardena, J. (2022). The linear framework: Using graph theory to reveal the algebra and thermodynamics of biomolecular systems. Interface Focus 12, 20220013. DOI: 10.1098/rsfs.2022.0013.

S12. Hill, T. L. (1989). Free Energy Transduction and Biochemical Cycle Kinetics; Springer: New York.

S13. Astumian, R. D., and Robertson, B. (1993). Imposed oscillations of kinetic barriers can cause an enzyme to drive a chemical reaction away from equilibrium. J. Am. Chem. Soc. 115, 11063–11068. DOI: 10.1021/ja00077a001.

S14. Astumian, R. D. (2018). Trajectory and Cycle-Based Thermodynamics and Kinetics of Molecular Machines: The Importance of Microscopic Reversibility. Acc. Chem. Res. 51, 2653–2661. DOI: 10.1021/acs.accounts.8b00253.

S15. Penocchio, E., Bachir, A., Credi, A., Astumian, R. D., and Ragazzon, G. (2024). Analysis of kinetic asymmetry in a multi-cycle reaction network establishes the principles for autonomous compartmentalized molecular ratchets. Chem 10, 3644–3655. DOI: 10.1016/j.chempr.2024.07.038.

S16. Baixeras Buye, J., Gallagher, J. M., and Leigh, D. A. (2026). Structure-performance relationships for catalysis-driven molecular machinery. Chem 12, 102742. DOI: 10.1016/j.chempr.2025.102742.

S17. Falasco, G., Rao, R., and Esposito, M. (2018). Information Thermodynamics of Turing Patterns. Phys. Rev. Lett. 121, 108301. DOI: 10.1103/PhysRevLett.121.108301.

S18. Penocchio, E., Rao, R. and Esposito, M. (2019). Thermodynamic efficiency in dissipative chemistry. Nat. Commun. 10, 3865. DOI: 10.1038/s41467-019-11676-x.

S19. Valkenier, H., López Mora, N., Kros, A., and Davis., A. P. (2014). Visualization and Quantification of Transmembrane Ion Transport into Giant Unilamellar Vesicles, Angew. Chem. Int. Ed. Engl. 30, 2137–2141. DOI: 10.1002/anie.201410200.
